# Virtual Tumors Enable Prediction of Personalized Therapeutic Combinations for Non-Small Cell Lung Cancer

**DOI:** 10.1158/0008-5472.CAN-25-2476

**Published:** 2026-09-15

**Authors:** Matthew A. Clarke, Charlie George Barker, Ashley Nicholls, Matt P. Handler, Lisa Pickard, Amna Z. Shah, David Walter, Etienne De Braekeleer, Udai Banerji, Jyoti S. Choudhary, Saif S. Ahmad, Ultan McDermott, Gregory J. Hannon, Jasmin Fisher

**Affiliations:** 1 UCL Cancer Institute, University College London, 72 Huntley Street, London, WC1E 6DD, UK; 2 Cancer Research UK Cambridge Institute, University of Cambridge, Cambridge, CB2 0RE, UK; 3 The Institute of Cancer Research, 123 Old Brompton Road, London, SW7 3RP, UK; 4 AstraZeneca, Oncology R&D, 1 Francis Crick Avenue, Cambridge, CB2 0AA, UK; 5 Cancer Research Horizons, Cambridge, CB2 0RE, UK; 6 AstraZeneca, Discovery Science R&D, 1 Francis Crick Avenue, Cambridge, CB2 0AA, UK

## Abstract

The disease burden from non-small cell lung cancer (NSCLC) adenocarcinoma is substantial, with a million new cases diagnosed globally each year and a 5-year survival rate of less than 20%. The lack of therapeutic options personalized to individual patients leads to high variation in survival. The combination of patient stratification with personalized treatment has the potential to improve outcomes; however, the variation in mutations found in NSCLC adenocarcinoma patients makes experimentally determining treatment combinations time-consuming and expensive. Here, we developed an interpretable mechanistic model to decipher complex signaling interplay and guide personalized therapy in NSCLC adenocarcinoma. This ‘virtual tumor’ model encompassed key tumor intrinsic oncogenic signaling pathways, for efficiently predicting rational drug-drug and drug-radiotherapy combination therapies in NSCLC. Diverse genetic profiles were simulated for testing over 10,000 therapeutic strategies to identify optimal approaches to overcome resistance mechanisms specific to genetic profiles and p53 status. The virtual tumor model reproduced drug additivity screens, predicted radio-sensitizing genes validated in a CRISPR screen, and identified 53BP1 as a potential drug target that improved the therapeutic window during radiotherapy. A 19-gene signature derived from the virtual tumor framework stratified patients most likely to benefit from radiotherapy, which was validated using TCGA data. These results demonstrate the utility of virtual tumors to predict effective therapeutic combinations and present a computational resource for large-scale screening of personalized therapies to guide clinical decision-making in NSCLC patients.

## Introduction

Lung cancer is the leading cause of cancer-related deaths worldwide, resulting in an estimated 1.8 million deaths annually. Non-small cell lung cancer (NSCLC) adenocarcinoma accounts for ~85% of lung cancer cases, with 5-year survival rates of below 10% in the majority of cases (metastatic), though rising to around 65% if treated early when the cancer is localized^1,2^ (SEER21, see Methods). Treatment options have advanced substantially over the last 10 years, with new surgical approaches, chemotherapeutic drugs and better targeted radiotherapy, with patients now often treated with several of these in combination^1^. However, overall survival remains low^1,2^, in part because current approaches do not account for the diversity of oncogenic mutations driving NSCLC^3^. Furthermore, in cases where targeted therapy is applicable, resistance is inevitable, with the majority of patients treated with first-generation Epidermal Growth Factor Receptor (EGFR) inhibitors developing resistance after 6-9 months of treatment^1,4^. A lack of understanding of how tumor genetics affect response to therapy, and the mechanisms underlying resistance, limit our ability to rationally design treatments that pre-empt and overcome resistance, and personalize therapeutics to maximize individual patient outcomes^5^.

Radiotherapy delivers a high dose of radiation to cancer cells to cause the rapid accumulation of double-stranded breaks (DSBs)^6^. If unrepaired, these DSBs trigger cell death^7^, while healthy cells are better able to repair these breaks and survive. Radiotherapy has a significantly lower treatment-associated mortality than surgery^8^, although the efficacy of surgery is higher than that of radiotherapy in terms of overall survival^9^, owing in part to adaptation of the tumor and evolution of radio resistance-associated mutations^10^. Extending the benefits of radiotherapy to more patients and improving efficacy to reduce the chance of resistance emerging requires better understanding of how variation in treatment response is driven by differences in driver mutations. This will allow tailoring of radiotherapy to patient biology by combining it with radio-sensitizing treatments.

**Figure 1:**
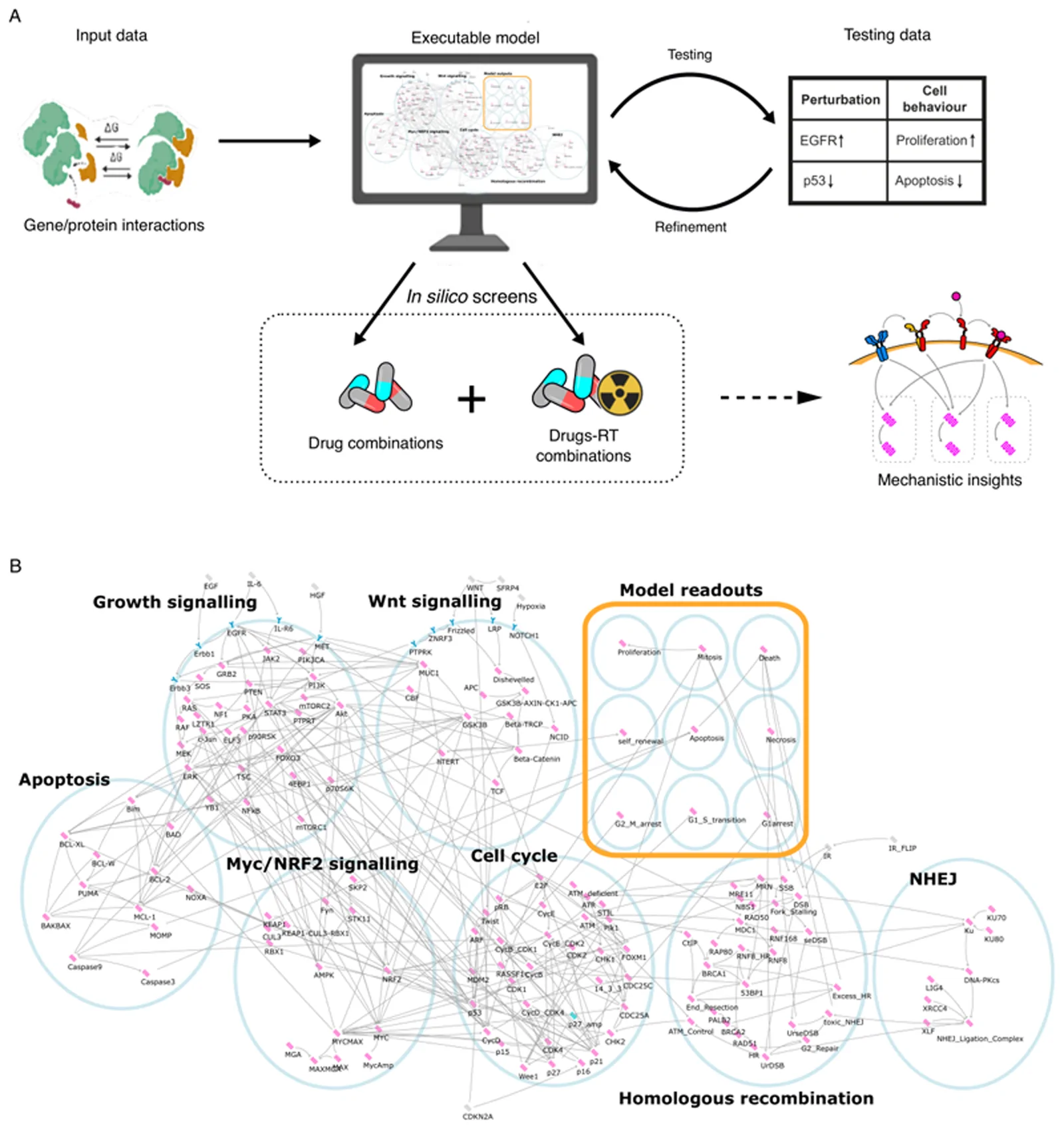
Outline of the workflow and executable NSCLC adenocarcinoma model. **A)** Schematic showing the workflow used to construct, test, and analyze the model (See Methods). **B)** A network visualizing the architecture of the executable model as seen in the BMA tool. Subnetworks are enclosed by a blue circle, model inputs are colored grey, receptors blue and cytosolic proteins pink. Edges represent regulatory interactions between proteins: activating edges (→) and inhibiting (⊣). Phenotypic readouts are enclosed in a yellow box. Created in BioRender. Fisher, J. (2026) https://BioRender.com/40aw1et.

Recent advances in modelling patient biology as executable computer programs are now beginning to uncover the principles underlying how the genetics of a cancer contributes to its behavior and the potential mechanisms that give rise to phenotypes such as therapeutic resistance^11–18^. Here, we present a bespoke executable model of NSCLC adenocarcinoma oncogenic signaling that covers key pathways including the cell cycle, apoptosis cascade and DNA damage response (DDR) (Figure 1). This ‘virtual tumor’ is the first mechanistic network model allowing the *in silico* prediction of radiotherapy response in NSCLC, providing a computational framework to decode the influence of tumor genomics on treatment sensitivity, and to facilitate the rational design of personalized therapeutic combinations that can improve patient outcomes. This model mechanistically link oncogenic genetics and signaling, by representing biological processes as nodes within a qualitative network, with functions determining the activation of biological components^19^, allowing understanding of how therapy interacts with oncogenic mutations to understand how EGFR and TP53 mutations determine response to combination treatment by MAPK and AKT inhibitors.

Here we use this virtual tumor model to explore 136 possible radio-sensitizing treatments across a panel of 7 cancer cell lines and explore the effect of specific mutations on treatment sensitivity. We use the model to screen ~66,000 combinations to find the optimal therapeutic strategy for a given genotype, with these showing significant correlation with an experimental drug screen. We exploit the interpretable nature of our *in silico* model to understand why these sensitivities occur and how specific mutations determine the NSCLC adenocarcinoma responses to therapy. We then perform *in silico* screens for perturbations that can sensitize cancer cells to radiotherapy while protecting healthy cells, as well as overcome p53-driven radiotherapy resistance, and validate these results with CRISPR screening. Overall, our platform is a powerful tool for understanding therapeutic responses to treatment in NSCLC. To our knowledge, this represents the first general-purpose, virtual cancer model capable of predicting both targeted therapy drug combinations and their interactions with radiotherapy. Harnessing the power of executable models will provide a more complete understanding of how genetics contributes to oncogenic signaling and enable therapy to be tailored to the genetics underlying the cancer, improving treatment outcomes for patients.

## Material and Methods

### Qualitative networks

We represent the NSCLC adenocarcinoma signaling network as a discrete qualitative network^19^ (Figure 1). This represents each gene, protein, or process as a node with multiple finite values of the range (0-3), where 0 represents very low activity e.g., due to a loss-of-function mutation, 1 represents normal behavior, 2 elevated activity and 3 represents very high activity e.g., due to a gain-of-function mutation. For example, this allows us to capture normal rates of cell turnover by a level 1 of the Cell Death node, which reduces to 0 in some cancers, rises to 2 in normal tissue under maximal radiation dose, and allows detection of unwanted radio-sensitization of these cells, when this rises to 3. This was built, tested, and analyzed using the open source webtool, BioModelAnalyzer (BMA, https://biomodelanalyzer.org) and associated command line tools (https://github.com/hallba/BioModelAnalyzer). The model and code for analysis are available at https://github.com/jfisher-lab/NSCLC and https://codeocean.com/capsule/0298466/tree/v1 Nodes in the network represent biological factors such as proteins or subunits of proteins. Input nodes represent growth factors, or in some cases amplification as with ATM. Output nodes are representing phenotypes such as death and proliferation. Regulatory interactions (both activating (→) and inhibitory (⊣)) are represented by edges between nodes (Supplementary Table S1). The level of activity of a node responds to the upstream levels of regulators of that node. This is determined by a mathematical function, unique to each node that integrates the levels of the upstream regulators to determine the target level of the target node at the next time-step, with the activity of each node changing by at most 1 unit per time step. This function is termed the target function. The default target function is the average of the activating upstream regulators, negated by the average of the inhibiting regulators (*avg(pos)-avg(neg)*). More complex functions are used as needed to recapitulate specific experimental behaviors (see below). These target functions along with corresponding references are described in Supplementary Table S2. A summary of the nodes included, and their HUGO gene symbol are summarized in Supplementary Table S3 and how these are mutated in cell lines in Supplementary Table S4 and Supplementary Figure S1A-B.

For a specified starting state of all nodes, the model will update node values synchronously, such that there will be one deterministic stable attractor of the network. This attractor can be a fixed-point attractor with one end state, or it can be a loop of states that the model cycles through, called a cyclic attractor. We test the model from all possible initial states, which allows for multiple attractors to be reachable, termed a bifurcation. In the case of a loop or bifurcation, the upper and lower bounds across all attractors are returned. This is done using the algorithm described in Cook *et al*.^20^. The algorithm for network simulation is found in Schaub *et al*.^19^ and the bounds and reported midpoint are given in the Supplementary Data S1. If there is no single attractor, we use the midpoint of the upper and lower bound as an approximation of the overall level of the node.

### Computational model construction and testing

**Figure 2:**
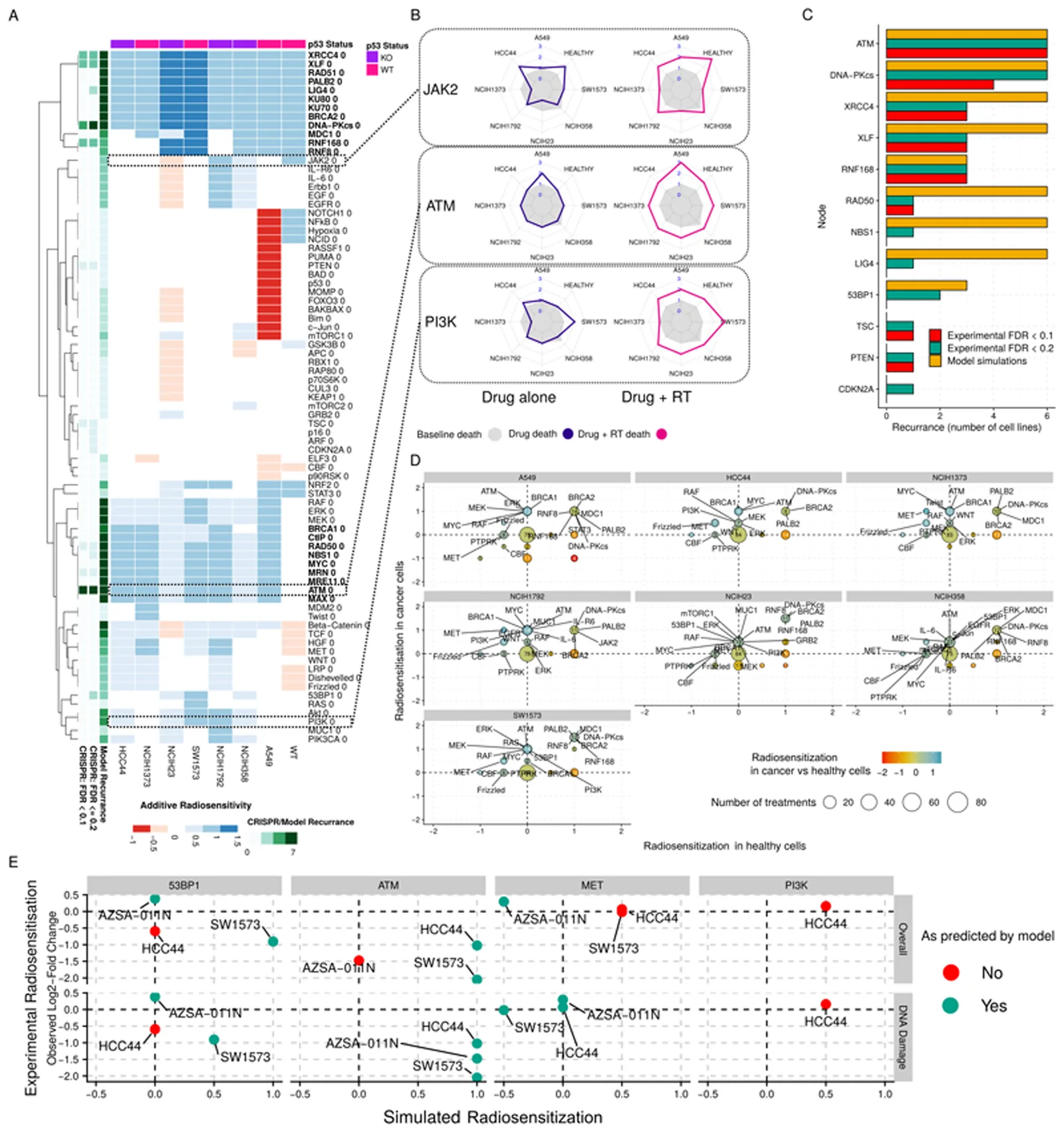
Systematic screen of perturbations on the *in silico* model reveals inhibition of ATM and 53BP1 as radio-sensitizing agents. **A)** Heatmap showing predicted radio-sensitizing effect *in silico* knockouts (rows, node value set to 0), on NSCLC adenocarcinoma cell lines (columns). Additive radio-sensitivity (see Methods) is increase (blue) or decrease (red) in cell death in the combination of radiotherapy and knock-out compared to the most effective of radiotherapy or knock-out applied alone. Side panels (green) show the number of cell lines where treatment was radio-sensitizing in the model or CRISPR screen (at FDR ≤ 0.2 and < 0.1). We count a recurrence if radiosensitivity is observed at this threshold for any timepoint in the screen. Broadly radio-sensitizing knockouts in bold. **B)** Radar plots of levels of death after drug treatments (for ATM, 53BP1 and JAK2) (dark blue), after drug treatment and irradiation (magenta) and baseline (grey). Specific broadly radio-sensitizing drug combinations outlined in black in panel A. **C)** Number of cell lines in which knockouts were predicted to be effective in network model (yellow) and CRISPR screen with FDR ≤ 0.2 (green) or FDR < 0.1 (red). Note: p16 and p14ARF (controlled by CDKN2A) knockouts were both effective and are grouped under this gene. **D)** X-axis change in healthy cell radio-sensitivity. Y-axis relative change in cancer cell radio-sensitivity. Blue is more radio-sensitization of cancer than healthy; red vice-versa. Point size is number of knockouts that have this effect. **E)** Observed Log2-Fold-Change (y) vs predicted radio-sensitization for genes in focused CRISPR screen (x). Values shown are for maximum time point, for others see Supplementary Data S3. Blue for validation (FDR<0.1 for cases where model predicted sensitization), red otherwise. AZSA-011N cells are healthy cell organoids. Genes for which knockout was essential at all timepoints removed. Top row is predicted radio-sensitization measured by change on overall cell death (measured by node ‘Death’), bottom row is predicted radio-sensitization measured by DNA-Damage specific cell death (measured by node ‘Necrosis’).

**Figure 3:**
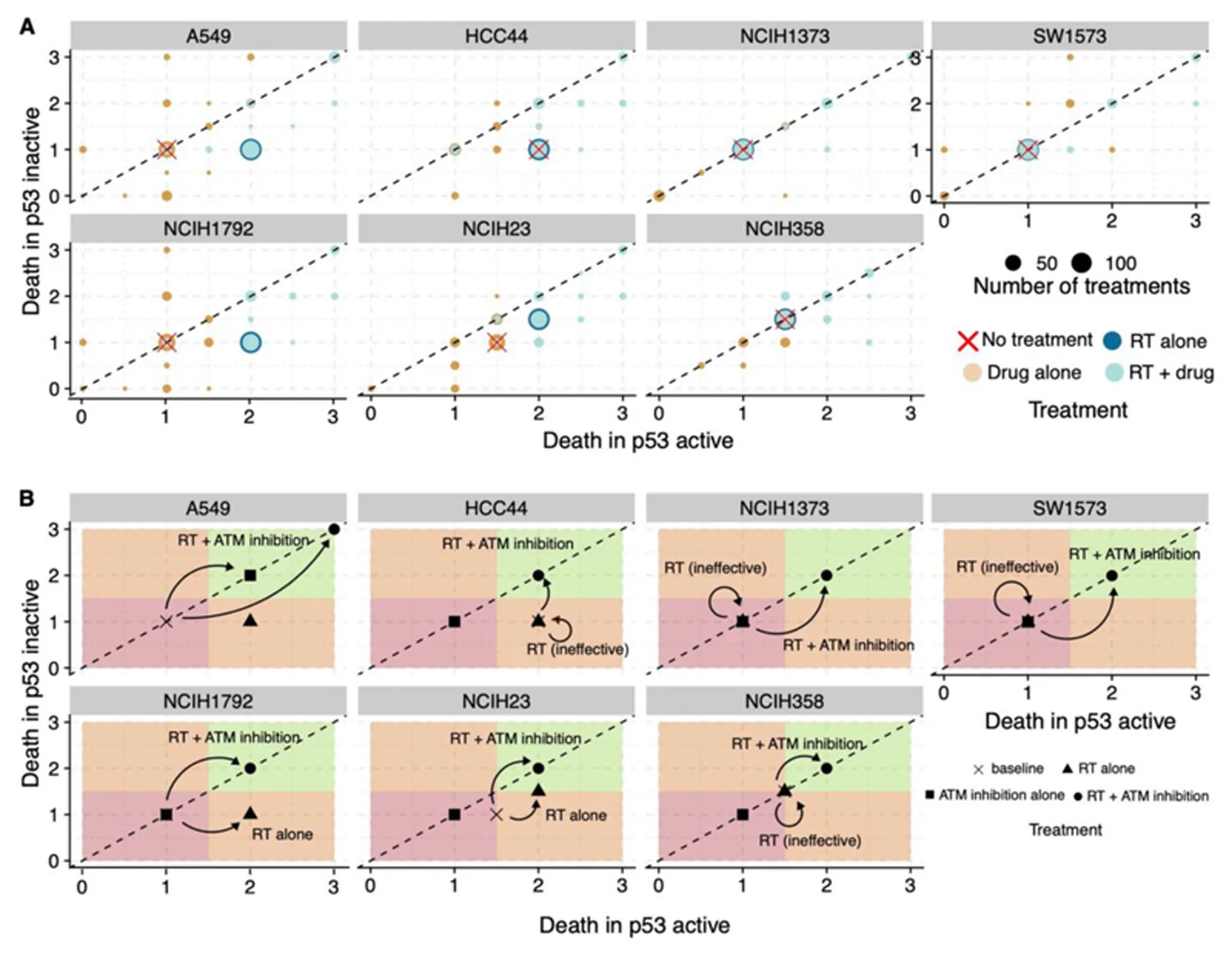
Radio-sensitizing knock-outs increase death in a manner that is agnostic to TP53 status. **A)** Levels of death predicted in cell lines modified to have TP53 active (x-axis) and inactive (y-axis). Colors denote type of treatment (dark blue: drug treatment alone, light blue: radiotherapy (RT) alone, orange: radiotherapy and drug treatment combined) Size denotes frequency of treatments in that position. Knock-outs below dotted line are less effective in TP53 inactive cases then in TP53 active, knock-outs above are more effective. **B)** Death in TP53 deficient (y-axis) and proficient(x-axis) cell lines showing the differences of radiotherapy alone vs with ATM knock-out. Quadrants colored to denote desirable locations for therapeutic outcomes. Green: high cancer death regardless of TP53 mutational status, red: low death regardless of TP53 mutational status and orange: knock-out efficacy is dependent on TP53 mutational status. Arrows show how ATM knockout moves radiotherapy-induced cell death from below the dotted line (less effective with inactive TP53) to the dotted line, indicating that radiotherapy + ATM inhibition overcomes TP53 inactivation.

**Figure 4:**
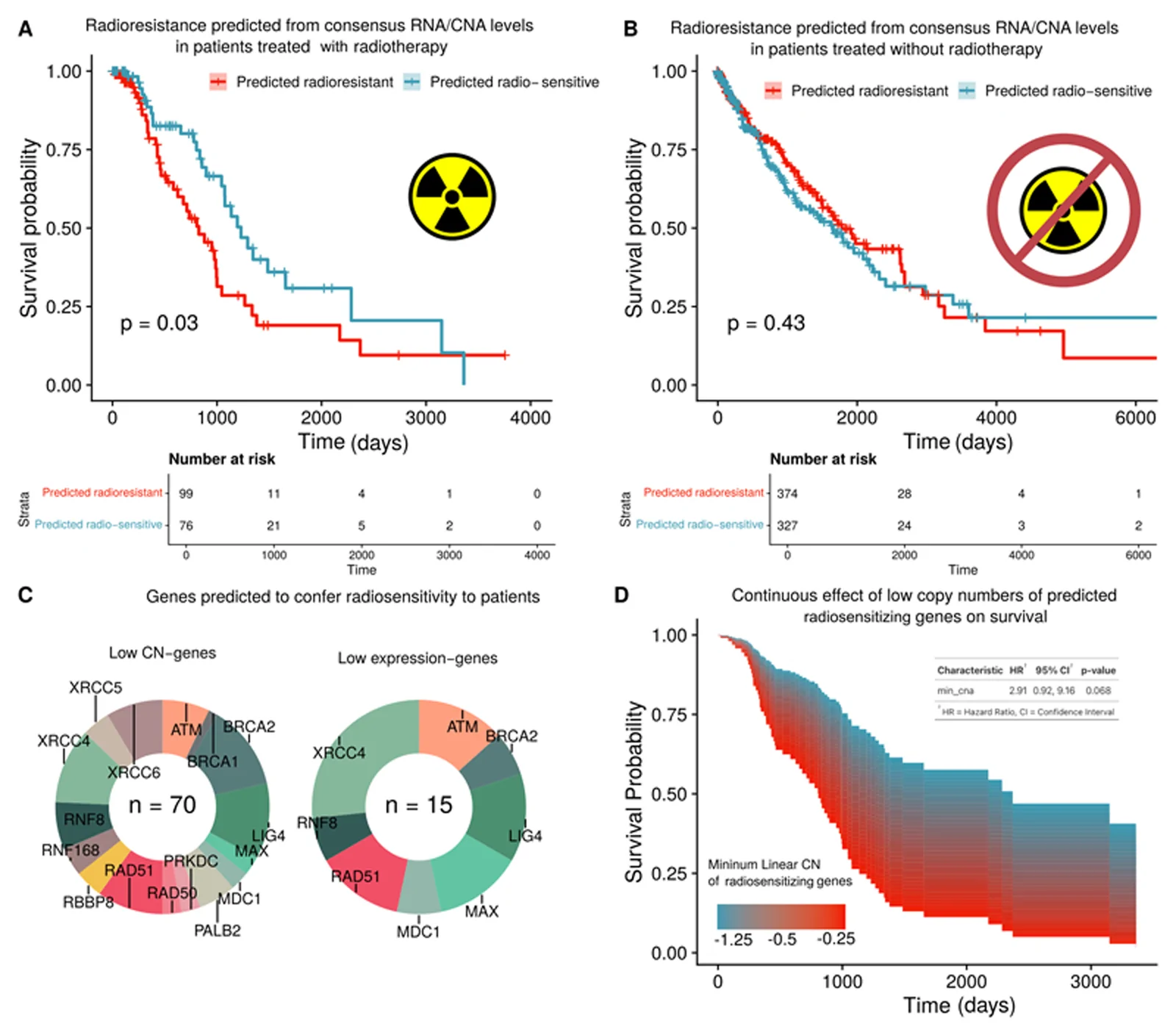
*In silico* predictions stratify radiation-treated patients into radiosensitive and radioresistant strata. **A, B)** Kaplan-Meier survival curves showing differences in survival between predicted radioresistant patients (red) and radiosensitive patients (blue) for patients treated with radiation (B) and without (C). X-axis indicates the time (days) since diagnosis. Y-axis indicates survival probability. Beneath are tables showing the number of patients surviving at each time point. **C)** Pie-charts showing genes with the lowest copy number (left) or expression (right) for each patient predicted to be radiosensitive. **D)** Continuously colored survival area plots showing the effect of the minimum copy number of radio-sensitizing genes on survival. X-axis indicates time (days) since diagnosis, y-axis indicates the survival probability, color indicates minimum copy number of genes within our predicted set, (blue low copy number, red high).

Experimental evidence for each model edge is described in Supplementary Table S1. These interactions were manually curated as pathway databases were not sufficiently accurate^21,22^. We focused on pathways connecting the known mutations in the cell lines we selected (see below). We further manually curated a separate set of 26 papers describing the behavior of different NSCLC adenocarcinoma cell lines under various perturbations and conditions. From these papers 69 separate experimental conditions formed a specification that was used to verify the performance of the network model. This is described in Supplementary Table S5. Comparison to the specification is used to iteratively improve the model through the addition of interactions (edges) and updates to the target functions. We aimed to find a minimal network that could reproduce the majority of the specification, to keep the model at a size that allowed mechanistic interpretability, without omission of known interactions between nodes in the model. Target functions were based on biological understanding from the literature, with reasoning and citations provided in Supplementary Table S2. When information on the strength of an interaction was not known, we chose those that provided the best fit to the specification. Accuracy calculated in two ways. First, we calculated accuracy where an experiment was considered recapitulated if the predicted direction of change of the model is the same as that of the experiment (Supplementary Table S5). Here the network model achieved an accuracy of 91% Second, we used Quadratic Weighted Kappa (QWK), a measure that can be used to compare two sets of categorical data with inherent order (ordinal data), as we have due to the discrete nature of the network predictions. This statistic measures if the prediction of the model and the true experimental value are different, weighted by the size of disagreement, with the weights growing quadratically as the ratings become more different. It provides a score between -1 and 1, with 1 being perfect agreement, 0 being no better than random, and -1 being perfect inverse agreement. The model achieved a QWK of 0.81 (Supplementary Figure S1C). These data consist of our training data, with validation being on the unseen experimental results from the CRISPR screen and TCGA data (Figures 2-4).

### Mutational profiles for all cell lines were drawn from Cell Model Passports

To model cell lines we used mutational data from Cell Model Passports (RRID: RRID:SCR_027682) as of 04/01/2023. Cell lines were chosen to cover a range of expected radio-sensitivities for RAS-driven NSCLC lung adenocarcinoma^23–27^. Mutations were modelled if they were listed as a driver or a deletion for a node in the model. If annotated as a gain of function the respective node was set to the maximum value, if a loss of function or a deletion the node was set to zero, except in the case of ATM. After the results of the first CRISPR screen, we observed that ATM knock-out was still effective in NCIH1373 (RRID:CVCL_1465) and NCIH23 (RRID:CVCL_1547) cells despite an ATM mutation observed in both, therefore we model this as a deficiency but not complete homozygous knockout of ATM. We focused on annotated driver genes and complete deletions so as not to over-specify the model, and to focus on those changes that were likely to be consistent within the population of cell lines^28^.

### Comparison with experimental dependency screens

Genomic data were obtained from the DepMap (RRID:SCR_017655) 24Q4 public release. EGFR amplification was identified using the *Omics Absolute Copy Number Gene Public 24Q4* dataset, with cell lines showing absolute EGFR copy number >10 classified as *EGFR*-amplified. Somatic mutation data were extracted from DepMap’s extended mutation calls and mutations were annotated by functional consequence (loss- or gain-of-function) based on the *mutation_effect* field. For each cell line, a consensus mutation profile was derived across *TP53, PIK3CA, KRAS*, and *EGFR (Erbb1)* and cells were simulated based on this profile in the qualitative model.

Experimental gene dependency data were obtained from DepMap’s CRISPR (Chronos) and RNAi (DEMETER2) datasets. These dependency scores were compared with the models predicted effect of single-gene perturbations classified as *Sensitive* (predicted ΔProliferation ≤ –1) or *Insensitive* (ΔProliferation > –1). For each gene–cell line pair, dependency score distributions were compared between *Sensitive* and *Insensitive* groups using two-sided Wilcoxon rank-sum tests.

Drug response data were obtained from the DepMap PRISM Repurposing 24Q2 dataset and matched to model perturbations based on annotated compound targets using the method described above. For each cell line, predicted perturbation effects were mapped to corresponding drug targets and compared against PRISM viability scores using the same binary sensitivity classification and Wilcoxon testing framework. Ordinal relationships between discretized predicted effect strength and drug response were evaluated using Kendall’s τ.

### Mutational profiling of cell lines used in radiotherapy CRISPR screens

Mutational data was derived using publicly available datasets: DepMap (RRID:SCR_017655) and Cell Model Passports (RRID: SCR_027682) and corroborated using western blotting (e.g., TP53 status). Radiation sensitivity was assessed using colony forming assay, and longer-term cell viability assays (e.g., cell-titre glo >7 day timepoint) and assessed against published literature (Supplementary Table S5).

### *In silico* modelling of combination therapies and monotherapy

To find effective treatments, we inactivate (set target function to a minimum) or activate (set target function to maximum) all nodes representing druggable nodes in the network either singly or in a pair-wise combination, using the BMA Command Line tool BioCheckConsole. For each node in the network, we curated a list of drugs that could target nodes within the network, finding 95 candidate drugs using the druggable proteome in The Human Protein Atlas (RRID:SCR_006710; Accessed 04/08/2023). We evaluate these perturbations by identifying the stable state of the network for death and proliferation as outlined in the ‘Qualitative Networks’ section above. To have a single value to predict cell growth we take the difference between Proliferation and Death.

For combination therapy we simulated these drugs together and studied their interaction using a value we describe as additivity. This is analogous to the experimentally determined Higher than Single Agent effect (HSA) and describes the growth of cells after combined drug treatment relative to the most effective (minimum viability) of the individual drugs in the combination. This is distinct from synergy, which requires a continuous dose measurement, and is therefore not possible to estimate from a discretized model.

Combination treatments were clustered using the k-means algorithm (using the base R package ‘stats’, RRID:SCR_025968) to find groups of therapies with similar levels of additivity across cell lines.

### *In silico* modelling of radiotherapy

To model the effects of radiotherapy, we increase the `Radiotherapy` node to maximum. This leads to increases in the single and double strand break nodes. To model the ability of the cell to repair these insults, we allow these nodes to activate repair mechanisms, while also activating `UrDSB` (Unrepaired DSB). If the repair mechanisms are active, as in healthy cells, this means that UrDSB is inhibited by the activity of those mechanisms to represent the initial insult due to radiotherapy being resolved without causing death, while any damage to the repair pathways will let DSB activate UrDSB and in turn leads to death. Unrepaired SSB are modelled as leading to single-ended or blunt ended DSB (seDSB and DSB) respectively but not leading to death by themselves. We focused on effects of maximum tolerated dose and so expect the model to be most accurate when the Radiotherapy node is either at 0 or at 3.

We model radio-sensitization as the increase in death, decrease in proliferation, or decrease in overall survival (proliferation - death) in the combination vs the most effective of the two possible monotherapies (knock-out alone or radiotherapy alone).

### CRISPR knockout of radioprotective and radio-sensitizing genes

A CRISPR screen was performed using a pooled library across the 6 NSCLC, Cas9-expressing, lines using 1Gy given twice over 24 hours (i.e., 2Gy in 2 doses over 2 days). Cells were irradiated using an IBL 637 Irradiator (CS 137 gamma rays below sample). Cells were harvested from day 7 to day 31 (up until cells reached 10-12 doublings) and DNA was prepared for next generation sequencing. Hits were identified using MAGeCK RRA (version 0.5.8, RRID:SCR_025016). Cut-offs of FDR<0.2 and FDR<0.1 were used to define significant depleted genes.

Due to the *in vitro* CRISPR screen considering all 20,000 human genes, when a knock-out was observed to have an effect in only a subset of the cell lines it was not possible to conclude whether this was due to a vulnerability contingent on a specific mutational profile, or the power of the screen. For this reason, we measure and compare the number of cell lines in which a knock-out was radio-sensitizing in the CRISPR experiments vs the *in silico* model (i.e., recurrence) for the 12 genes that led to radio-sensitization that are represented in the network model. We do not consider cases where the model predicts radio-sensitization that were not observed in the CRISPR screen, due to false-negative rates in large CRISPR screens^29^.

A second screen was conducted with a focused panel, across the SW1573 (RRID:CVCL_1720), SW1573 *TP53*^-/-^, A549 (RRID:CVCL_0023) and A549 *TP53*^-/-^ cell lines and AZSA-011N organoids. After 72 hours, we irradiated cell cultures with a single fraction of 4Gy. Post-irradiation, precisely after 1 hour, 24 hours, and 48 hours, irradiated and non-irradiated plates were fixed for immunofluorescence.

### Generation of normal tissue organoids

Patient derived lung organoids (PDO) were isolated from either from adjacent normal tissues from consented NSCLC patients at Royal Papworth Hospital via the Royal Papworth Hospital Tissue Bank application ID T02612 / East of England - Cambridge East Research Ethics Committee reference 18/EE/0269 (model AZSA-011N) or were purchased from HUB Organoids (Merck KGaA) under license and approved by Institute Review Board (ref AO/mk/22/500793) following the ethical guidelines set out in the World Medical Association Declaration of Helsinki. All patients provided the appropriate informed written consent. All samples were additionally approved for use by AstraZeneca's internal Human Biological Samples governance team.

### Cell culture

HCC-44-Cas9 (RRID:CVCL_2060), SW1573-Cas9 (RRID:CVCL_1720), H1792-Cas9 (RRID:CVCL_1495), H1373-Cas9 (RRID:CVCL_1465), H358-Cas9 (RRID:CVCL_1559) and H23-Cas9 (RRID:CVCL_1547) cell lines were maintained in RPMI 1640 (Gibco) supplemented with 10% FBS (Biowest), 1% GlutaMax (Gibco) and 1% pen/strep (Gibco). Cell lines were passaged every 3/4 days by dissociating to single cell using Accutase (Biowest). All Cas9 cell lines were banked within first 10-15 passages and all experiments would have been completed within 10-15 passages, with the majority completed within 10. All cell lines were acquired from the AstraZeneca Global Cell Bank^30,31^. Cell line authentication was performed using STR and an IDEXX reference panel. Mycoplasma testing was performed using a quantitative PCR method on DNA extracted from cells using PhoenixDx^®^ Mycoplasma Mix. Mycoplasma testing was performed last on 1 July 2024 as part of routine screening.

### Genome-wide pooled CRISPR/Cas9 radiation screens in 2D human cancer cell lines

HCC-44-Cas9, SW1573-Cas9, H1792-Cas9, H1373-Cas9, H358-Cas9 and H23-Cas9 cell lines were transduced with Yusa_v3 gRNA library in the presence of 8µg/ml polybrene to achieve a transduction rate of ~30%. Three days post transduction, the percentage of BFP+ cells was checked by flow cytometry (Melody). Puromycin selection was started and maintained until %BFP was >90%. A baseline pellet was collected from all cell lines at this point, and cells were then seeded for irradiation and non-irradiation controls into 2-layer cellStacks with two technical replicates each. The cells were treated with a fractionated total dose of 2 Gy (1 Gy daily over 2 consecutive days) using an IBL 637 Irradiator (CS 137 gamma rays below sample), to achieve a growth inhibition of ~20%. Post-irradiation, treated and untreated controls were passaged into 5-layer cellStacks and maintained in such for the remainder of the screen. During the screen, cells were passaged every 3/4 days. Pellets were collected on Days 7, 14, 21, etc until the untreated cells reached 10-12 cell doublings.

### Custom CRISPR/Cas9 radiation screens in 3D human normal lung organoid lines

AZSA-011N-Cas9 and HUB-07-A2-051-Cas9 normal lung 3D human organoid lines were transduced with the bespoke 1242 sgRNA / 197 gene library in the presence of 8µg/ml polybrene to achieve a transduction rate of ~30%. Ten days post transduction, the percentage of BFP+ cells was checked by flow cytometry. Organoids were expanded for a further 14 days before single cell sorting for a pure population of +BFP cells using BD FACSMelody and FACSymphony™ S6 Cell Sorters. A baseline pellet was collected from both cell lines at this point, and cells were then seeded for irradiation and non-irradiation controls into 6-well tissue culture plates with two technical replicates each. The organoids were treated with a fractionated total dose of 2 Gy (1 Gy each day for 2 consecutive days) using the RadSource RS1800Q (at 25% power and dose rate of 0.85 Gy/min) to achieve a growth inhibition of ~30%. Treated and untreated controls did not require passaging and were harvested every 7 days. Pellets were collected on Days 7, 14, 21, and 28, with the non-irradiated control reaching ~3-5 cell doublings. Organoids lines were maintained in 80% BME throughout.

### Genomic DNA isolation and Next Generation Sequencing (NGS)

Genomic DNA (gDNA) was isolated using the QIAamp DNA Blood Midi or Maxi Kit (Qiagen) according to the manufacturer’s instructions. NGS libraries were prepared in a two-step process: the integrated lentiviral cassette containing the gRNA was amplified from gDNA. Forward primer 5’ ACACTCTTTCCCTACACGACGCTCTTCCGATCTCTTGTGGAAAGGACGAAACA 3’ with reverse primer 5’ TGACTGGAGTTCAGACGTGTGCTCTTCCGATCTACCCAGACTGCTCATCGTC 3’ was used for this. PCR reactions with 5µg gDNA per well were set up using the Q5 Hot Start High-Fidelity 2× Master Mix (NEB #M0494) in a total volume of 50µl. PCR reactions were scaled accordingly to amplify the gRNAs at a coverage of at least 200-fold. The PCR products were then purified using QIAquick PCR Purification Kit (Qiagen #28106). Final NGS libraries were generated using 2.5ng of the purified 1st PCR product using the dual-indexing Illumina-compatible DNA HT Dual Index kit (Takara #R400661). 2nd PCR products were purified with AMPure XP beads (Beckman Coulter #A63881) at a 0.7 ratio. Purified 2nd step libraries were quantified using the Qubit dsDNA Quantification Assay Kit (ThermoFisher) and sequenced on NovaSeq6000 by PE50bp with a 30% PhiX spike-in.

### Genetic modification of p53 status of NSCLC cell lines

The SW1573 line underwent TP53 knockout using a standard CRISPR-based knockout approach to generate a polyclonal TP53 knockout population. Loss of TP53 was confirmed using PCR and western blotting.

### Stratification of patient data using results from model

Clinical and genomic data for lung adenocarcinoma (LUAD) and lung squamous cell carcinoma (LUSC) were obtained from The Cancer Genome Atlas (TCGA) Research Network (https://www.cancer.gov/tcga, RRID:SCR_003193) and the cBioPortal platform (RRID:SCR_014555). Lung cancer survival statistics were obtained from SEER21 (RRID:SCR_006902). Clinical data was categorized based on whether patients received radiotherapy or not, using the patients not receiving radiotherapy as a positive control. Molecular data included RNA expression and Capped relative linear copy-number (CN) values for each gene (from Affymetrix SNP6) and was extracted via the TCGAretriever R package. To identify patients with low expression or alterations in the genes of interest, the CNA and RNA datasets were combined for both LUAD and LUSC. We identified patients as radiosensitive if they had gene expression or CN values below specific quantile thresholds (5th percentile for CNA, 10th percentile for RNA) for at least one gene in our model-defined set of radio-sensitizing genes. To associate patient strata with clinical outcomes we used the Cox proportional hazards regression model in R.

### Analyzing interaction of MYC/ATR activity in patient data

Full RNA-seq counts for TCGA LUAD and LUSC samples were obtained from Xena (RRID:SCR_018938). Gene sets of interest, including ATR- and MYC-associated pathways, G2M Checkpoint, DNA repair and NHEJ were retrieved from MSigDB (C2 and H collections, RRID:SCR_016863). Gene set activity scores were computed using a univariate linear model using the run_ulm function of the R package decoupleR (RRID:SCR_027127). Sample type was annotated as either Tumor or Healthy based on the TCGA barcode. To assess the relationship between MYC and ATR signaling on downstream signatures, linear models were fit for each signature using the formula HALLMARK_SIGNATURE ~ MYC_SIGNATURE x ATM_SIGNATURE + SAMPLE_TYPE, where HALLMARMK_SIGNATURE is either G2M progression or NHEJ and sample type is whether or not the sample is derived from tumor or healthy tissue.

### Statistical analysis of combination drug sensitivity

For drug combination we use the dataset available from Nair *et al*. (2022)^32^ and take all the drug inhibitors they used and compare them to our predictions. Nair *et al*. use HSA and so we compared our value for additivity (see above). For statistical analysis, we visualize t-test between HSAs of different treatments for different values of additivity that the model predicts (-3 through 1) and compare this to treatments predicted to be not additive. Also, to factor in the ordinal nature of the model’s prediction, we calculate overall correlation using Kendall’s tau test and bin experimentally measured HSA into 4 quartiles. Both t-tests and Kendall’s tau test were conducted in R (stats package, RRID:SCR_025968). Survival differences were evaluated using a Cox proportional hazards model. The relatively small number of patients in some stratified subgroups limits statistical power, which may explain the lack of significance observed in these analyses.

### Linear regression to identify genetic determinants of response

We separate the drug treatments into the clusters outlined above and train a linear model to relate drug response (measured by additivity) with the mutational profile of the cell lines. P values were adjusted for multiple hypothesis correction between each drug cluster using the method devised by Benjamini & Hochberg (1995)^33^.

## Results

### An executable network map of KRAS-, EGFR- and MET- driven NSCLC adenocarcinoma

To understand the contributions of oncogenic mutations in NSCLC adenocarcinoma to drug and radiotherapy response, we built an executable qualitative network model using the BioModelAnalyzer (BMA) tool (https://biomodelanalyzer.org, see Methods) following the workflow outlined in Figure 1A. The model describes the core pathways covering many common driver mutations in NSCLC patients^34^, including EGFR, AKT, JAK/STAT and WNT pathways, as well as commonly overexpressed genes such as MYC and NRF2. We modelled how these mutations drive changes in cell cycle and apoptosis. To model radiotherapy responses, we include the DNA damage repair (DDR) pathways, homologous recombination (HR) and Non-Homologous End Joining (NHEJ).

An extensive literature survey of 171 research papers identified interactions between genes and proteins in NSCLC adenocarcinoma resulting in a model with 160 nodes and 360 edges describing activating regulatory relationships or inhibiting relationships between biological components (Supplementary Table S1). To provide context to these interactions, each node has a corresponding target function defining its activity (Shown in Figure 1B, Supplementary Table S1, S2 and S3). The value describing the activity of nodes is discrete and ranges between 4 levels (0 to 3). A level of 0 indicates loss-of-function and 3 indicates gain-of-function.

### The NSCLC adenocarcinoma network model reproduces experimental data

To train the model, we collated a list of 26 publications reporting the behavior of NSCLC adenocarcinoma cell lines under different experimental conditions, separate from and not used to build the network structure of the model (Supplementary Table S4, S5). These publications describe the behaviors (e.g., growth rates, levels of apoptosis, protein levels) of different cell lines under perturbation by drugs and/or radiation. Cell lines were modelled by setting the nodes that represent genes with known driver mutations to the appropriate constant value (0 for loss-of-function and 3 for gain-of-function). Cell line mutation data were drawn from Cell Model Passports (RRID: RRID:SCR_027682) and our own experiments (see Methods, Supplementary Table S4 and Supplementary Figure S1). We then used the model to find the stable states of the system, which we compared to the phenotypes measured in the experiments (Supplementary Figure S1C). Comparison of the simulated behaviors with the published observations was used to iteratively improve the target functions of the nodes within the model until the model recapitulates the behaviors observed in the published experiments (see Methods, Supplementary Table S5). We also modelled the sensitivity of six cell lines to single drug treatment with and without radiotherapy (HCC44, NCIH1373, NCIH23, NCIH358, NCIH1792, SW1573) and compared to the published literature, to test the DNA-damage repair section of the model (Supplementary Figure S2, S3 S4). We examined common targeted therapies including EGFR^35^, MEK, MET,^36^ and DNA-PK inhibitors^37^ and further verified that the model reproduced the effects of knock-out experiments on key proteins in oncogenic processes, including KRAS^38^, MUC1^39^, NRF2^40^, JAK2^41^, RAD51^42^, ATM^43^, RNF8^44^, NOTCH1^45^ and TP53^46^. To identify treatments that maximize the difference, for the same dose, between the effect on cancer and healthy cells (maximize the therapeutic window) we also simulated healthy lung cells. We assume that the signaling processes in these cells are the same as in lung cancer except without driving oncogenic mutations. We tested this assumption by comparing to published experiments on short term normal tissue toxicity conducted using the immortalized epithelial lung cell lines HBEC3 and BEAS-2B^46,47^ (Supplementary Table S5).

Overall, when comparing the predictions of our model to a specification composed of the above published data (Supplementary Table S5) our model achieved a Quadratic Weighted Kappa (see Methods) of 0.81, where 1 is perfect agreement, indicating very accurate recapitulation when accounting for the ordinal proximity of the results (Supplementary Figure S1C). In addition to this comparison of the numerical outputs of the model, when more broadly verifying whether the model predicts whether a treatment will have a positive or negative effect on a cell behavior compared to the published experimental outcomes, we observe a 91% agreement in the direction and effect of treatment responses, (Supplementary Table S5; see Methods).Having reproduced existing experimental data, we next used the model to systematically predict the effect of unseen combinations of knock-outs in our panel of NSCLC adenocarcinoma cell lines.

### Targeted monotherapies inhibiting EGFR, RAS and STAT3 have an effective but narrow therapeutic window

To simulate personalized therapies with single targeted drugs, we systematically set the value of 95 druggable (see Methods, Supplementary Table S6 and Supplementary Figure S5) nodes in the model to 0 in six NSCLC adenocarcinoma cell lines (SW1573, NCIH1792, HCC827, PC9, A549 and NCIH1993) chosen for a range of driver mutations to reflect the genetic heterogeneity of NSCLC (See Supplementary Figure S1). By setting the value of nodes to a constant value of 0, we mimic the effect of inhibitors that could be used as targeted therapy. Overall, we observed that cancer cells are more resistant to targeted therapy than healthy cells when considering both cell proliferation and death levels (Supplementary Figure S2, Supplementary Data S2). Perturbations that are effective at killing cancer cell lines are in many cases also lethal to healthy cells (e.g., EGFR in HCC827, PC9 or KRAS in NCIH1993, NCIH1792 and A549) (Supplementary Figure S2). Inhibition of STAT3 kills cells in the A549 and NCIH1993 cell lines (Supplementary Figure S2), a pattern observed with all other potential treatments demonstrating how small differences in mutational profile can lead to total resistance to therapy.

When identifying potential cytostatic treatments, we identify more potential drug targets. This includes inhibiting MYC, which limits oncogenic growth to the basal growth rate of healthy cells while also reducing apoptotic signaling in all cell lines profiled apart from A549. These simulation results are further supported by our validation analyses using experimental dependency data from DepMap (Supplementary Figures S3-4). Across both the cell lines used for model specification and those not previously seen by the model, we observed significant associations between predicted sensitivity and experimental essentiality scores, consistent across both CRISPR and RNAi datasets. Generally, in our simulations, we see that healthy cell lines are more susceptible to treatment via the inhibition of single proteins than cancer cells (Supplementary Figure S2). This underlines the importance of using combination therapies to maximize the perturbation space to find cancer-specific treatment options with a good therapeutic window.

### NRF2 inhibition is predicted to protect healthy cells while 53BP1 inhibition sensitizes cancer cells to radiotherapy

We next sought to assess combining targeted therapy with radiotherapy to see whether this would improve predicted treatment efficacy. We therefore used the model to assess the radio-sensitizing effect of gene knockouts and compared the predictions to an *in vitro* CRISPR screen of radiation sensitive and resistant lung adenocarcinoma cell lines (HCC44, NCIH1373, NCIH23, NCIH358, NCIH1792, SW1573). We set one node at a time to a constant value of 0 to mimic a knock-out, in addition to cell line-specific mutations (Supplementary Table S4), and compared this effect with or without irradiation (IR), modelled through the value of the ‘IR’ node that activates nodes representing single (SSB) and double (DSB) strand breaks (Supplementary Figure S5). The effect of radiotherapy is modelled through the induction of single-strand breaks (SSB), double-strand breaks, and replication-induced or single-ended double strand breaks (seDSB)^48^. We modelled the impact of SSB on fork stalling and collapse, and the subsequent generation of seDSB^49^ as well as the downstream repair of DSB and seDSB by homologous recombination and non-homologous end-joining^50^, with unrepaired breaks and toxic chromosome fusions leading to increased cell death. We define an effective radio-sensitizing knockout as one that leads to more cell death as part of a combination compared to either the gene knockout or radiotherapy alone. We term the additive difference between cell death in the combination treatment and the most effective of these two monotherapies as ‘radio-sensitization’ (see Figure 2A).

The model predicts broad classes of radio-sensitizing knockouts. Some knockouts are radio-sensitizing in both healthy and cancer cells; primarily inhibition of the NHEJ pathway, as expected^51^. Other known treatments such as ATM inhibition^52^ are radio-sensitizing across all cancer cell lines, but we also see cell line-specific radio-sensitizing knockouts. For example, 53BP1 inhibition is particularly additive in SW1573 (Figure 2A-B). Such cell line specific behavior demonstrates the difficulty in maintaining an effective therapeutic window across diverse patient mutations and underlines the requirement for tailored therapeutic approaches to diverse mutational profiles.

We compared the model results to an *in vitro* CRISPR screen in the six NSCLC adenocarcinoma lines treated with radiotherapy (HCC44, NCIH1373, NCIH23, NCIH358, NCIH1792, SW1573) (Figure 2C). The model correctly predicts those knockouts that led to radio-sensitization in the *in vitro* CRISPR screen (9 out of the 12 cases) (Figure 2C).

The model suggests that the radio-sensitizing effects of knockouts outside of the NHEJ pathway are highly specific to mutational context, as we saw for many monotherapies (Figure 2A). In addition, when radiotherapy is applied, the model predicts similar levels of death in NSCLC adenocarcinoma and healthy cells (Supplementary Figure S5, S6). However, the degree to which a drug radio-sensitizes a cell (the difference between the combination of radiotherapy and knock-out compared to the most effective of either radiotherapy or knock-out applied alone) is often higher in the cancer cells than the healthy cells. There is a subset of cases where all the death in healthy cells is driven by radiotherapy, but most death in the cancer cells is driven by the combined effects therapy and radiotherapy. This suggests that by reducing the radiotherapy dose we could reduce the death in the healthy cells more than in the cancer cells and widen the therapeutic window. Combined with the personalized nature of radio-sensitization, this prompted us to query the model for knockouts that lead to the largest change in radio-sensitivity.

Figure 2D shows the predicted additive radio-sensitization (see Methods) of cancer cells versus healthy cells. Many treatments are predicted to radio-sensitize both cancer and healthy cells, for example those involved in DDR pathways. In the case of radio-sensitization of cancer cells specifically, we predict that inhibition of MYC is particularly effective and specific. This is partly due to the essential role of MYC in proliferation^53^, but also because of the regulation of NBS1 by MYC, which, as part of the MRN complex (MRE11, RAD50, NBS1), plays a key role in coordinating the response to DNA damage.

Conversely, we predict that loss of members of the PTP family (e.g., PTPRT), leads to reduction in radio-sensitivity in healthy cells, protecting them from toxicity, while having no effect on many of the cancer cell lines. This is due to an increase in JAK/STAT signaling, with a concomitant increase in MCL-W that suppresses apoptosis, and NRF2, which aids in repair of radiation induced DNA damage. This has less effect in cancer cells as they have already up-regulated NRF2. We examined death from DNA damage (through the node ‘Necrosis’) specifically as this may play a greater role in response to different radiotherapy doses than apoptotic death (Supplementary Figure S6). We predict that inhibition of NRF2 is particularly sensitizing to DNA-damage associated death in cancer cells, due to reduction in DNA damage repair^54^. Equally, suppression of the KEAP1/CUL3/RBX1 complex leads to an increase in NRF2 in healthy cells, protecting them, without aiding cancer cells, as these over-express NRF2 via mutant KRAS.

To test the ability of our model to predict radio-sensitizing targets, we assess a selection of these genes, MYC, 53BP1, MET, PI3K and ATM in a focused CRISPR screen in cancer cell lines (SW1573, A549, HCC44) and healthy cell organoids (AZSA-011N). Of these, MYC was unable to be tested as it proved essential in all cell lines. 53BP1 sensitized cancer cells and not healthy cells, in line with the model’s predictions, although it sensitized both SW1573 and A549 relative to healthy cells, where we predicted it would be effective only in A549. ATM sensitized all cancer cell lines, as predicted, as well as healthy cells, which the model did not predict. MET did not show the expected sensitization of the cancer cells (Figure 2E (top)), nor the protection of healthy cells (AZSA-011N, Supplementary Data S3). However, in our model we treat Apoptosis and DNA-damage associated death (Necrosis) separately. When examining the latter, we find that the model’s predictions align well with observation in all genes and cell lines, other than for HCC44 in the case of 53BP1 and PI3K (Figure 2E (bottom)), although 53BP1 may be essential for HCC44 (essentiality was detected at early time points but not later timepoints, see Supplementary Data S3).

### ATM inhibition overcomes radio-resistance conferred by TP53 deficiency

As a key regulator of DNA damage response which is often mutated in cancer, we wanted to explore the association between TP53 mutational status and radiotherapy response in cancer cell lines. Accordingly, we simulated the cell lines from our radio-sensitization screen with TP53 activated in cell lines where it has a loss-of-function mutation and inactivated in cell lines where it is competent (Supplementary Table S4). The model predicts that TP53 deficiency reduces death from radiation alone in most cell lines (Figure 3A, Supplementary Figure S7). However, when combined with a radio-sensitizing knock-out, there are many potential combinations that are predicted to induce death in cancer cell lines regardless of TP53 activation, overcoming the radio-resistance due to TP53 loss-of-function (orange points on dotted line, Figure 3A).

ATM knock-out is an example of a radio-sensitizing perturbation that is effective across all cancer cell lines, irrespective of TP53 mutation status (Figure 3B). Therefore, the model predicts that there is benefit in using radio-sensitizing agents targeting those proteins identified in our *in silico* knock-outs to improve the effect of radiotherapy for patients with a TP53 mutation. We experimentally validated these results by modifying SW1573 and NCIH1373 cell lines *in vitro* to flip the mutational status of TP53 (Figure 3 and Supplementary Figure S8). This is consistent with previous results across multiple tissues in mice^55^ and other NSCLC cell lines^26^. Indeed, ATM sensitizes cancer cells regardless of p53 status in all cancer cell lines for which this was tested in the focused CRISPR screen (A549 and SW1573), as predicted (Supplementary Data S3).

### Predicted radio-sensitizing targets stratify patient response to radiotherapy

Following our systematic *in silico* screen of radio-sensitizing knockouts, we observed that there was a subset of genes that affect radiation sensitivity agnostic to mutational background. These 19 genes are marked in bold on Figure 2 and include members of the DNA damage repair pathways (ATM, MDC1, XRCC6, XRCC4 etc.) as well as transcriptional regulators (MYC and MAX). We hypothesized that these candidate genes could be used to stratify patients as either radioresistant or radiosensitive. To explore this, we used the TCGA database (https://www.cancer.gov/tcga) of clinical data to see whether patients with low levels of gene expression (as measured by RNA-seq) or copy number variation (CNV) for our predicted radiosensitive genes survive longer after treatment with radiotherapy. We considered either of these properties to be analogous to the activity of the node within our network model, as both would affect the expression of these genes in response to IR and thus the efficacy of DDR. Patients with both low copy numbers (bottom 5%, n= 76) and of low RNA expression (bottom 10%, n=15) for at least one radio-sensitizing gene, compared to all patients, were determined to be radiosensitive (Supplementary Figure S9A, n=175).

When instead considering RNA expression and CNV separately, we found that with either definition of activity of a gene we see a lower hazard ratio (longer survival) for patients predicted to be radiosensitive after receiving radiotherapy (Supplementary Figure S9B-C, Cox proportional hazards regression; HR= 0.4299, P=0.06 for expression and HR= 0.6421, P=0.08 for CNV). When using both CNV and RNA to determine a consensus subset (see Methods) of radiosensitive patients we also see a significant difference in survival (Figure 4A, HR= 0.5842, P=0.03), suggesting that patients with a naturally lower activity of these genes have increased survival. When looking at patients that have not received radiotherapy (n=705, Supplementary Figure S10A) we see no significance (Supplementary Figure S10B-D), indicating that these genes are stratifying patients only in the presence of radiation (Figure 4B), consistent with our model’s prediction of these targets as potential radio-sensitizing agents. This association was consistently observed across both RNA expression and CNV data, strengthening the association of our signature with radio-resistance and demonstrating the robustness of our model across multiple molecular modalities.

Lastly, we investigated whether there was a continuous relationship between the lowest level of a radio-sensitizing gene in a patient and their survival following treatment with radiotherapy. This assumes that the level of radio-sensitization in a patient is rate-limited by each of our model-predicted radio-sensitizing agents. For each patient, we calculated the lowest linear copy number of each of the 19 genes (Figure 4C) and then used this continuous variable to predict survival in patients exposed to radiotherapy. The survival area plot for this analysis is shown in Figure 4D, showing significant association between the minimum CNV level of the radio-sensitizing genes and survival (Cox proportional hazards regression; HR=2.91, P=0.07). This association does not exist for survival among patients not treated with radiotherapy (HR=1.19, P=0.6), indicating this relationship only exists in the presence of radiation. Collectively, these findings demonstrate that low CNA and low RNA expression of our predicted radio-sensitizing genes are significant predictors of improved survival in patients receiving radiotherapy. However, our modelling and statistical analysis of clinical outcomes also shows that many patients are radioresistant and so this necessitates broader choices of therapeutic options.

### *In silico* screens identify MYC and ATR inhibitor as a cancer-specific treatment combination

**Figure 5:**
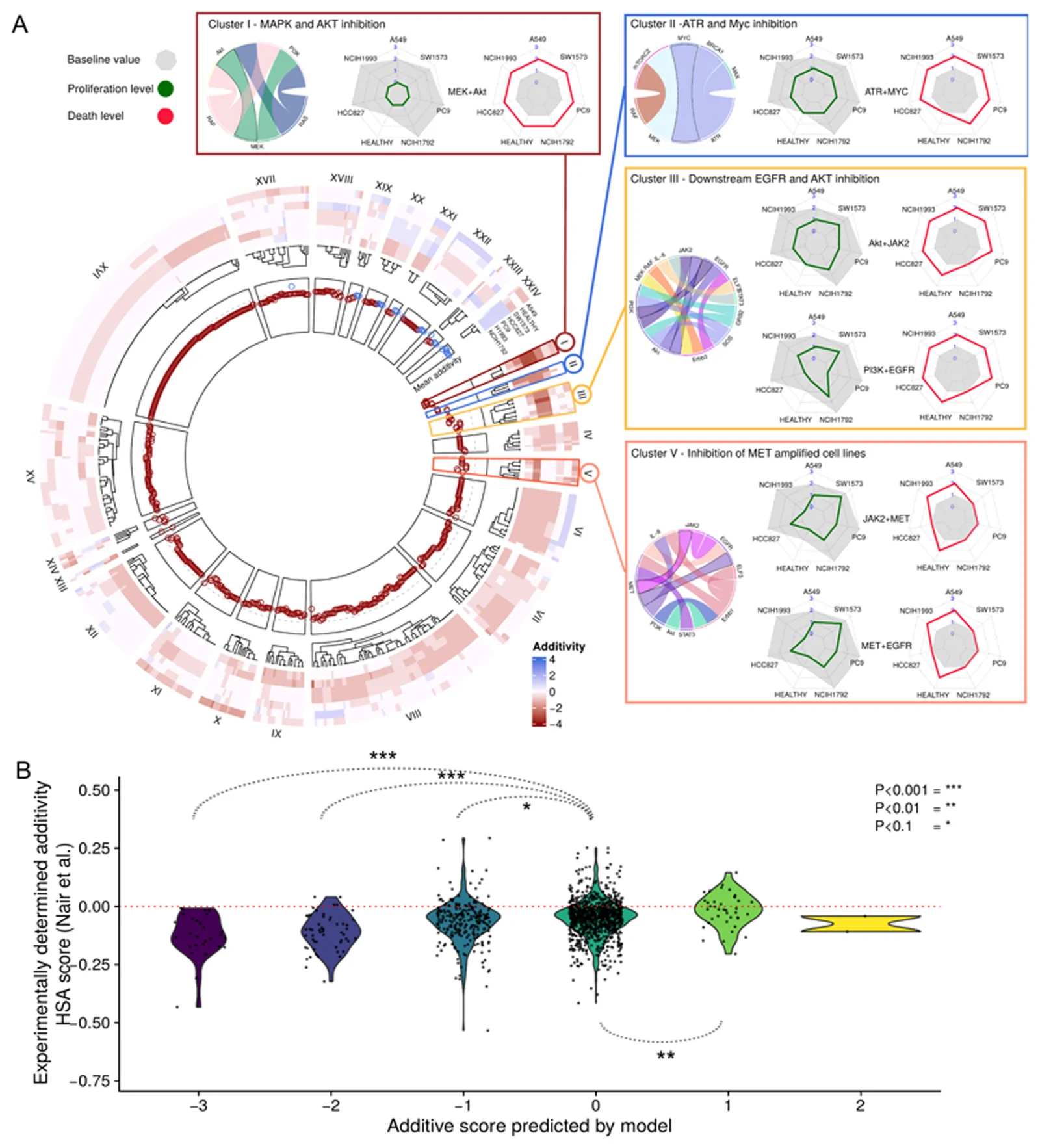
Systematic *in silico* combination screen on NSCLC adenocarcinoma network model. **A)** Circular heat map showing the additive effect (Methods) of drug combinations on selected NSCLC adenocarcinoma cell lines (full results in Supplementary Data S1 and S2). Columns (spokes of the wheel) represent drug combinations and are clustered into groups (I-XXIV) of drugs that have similar additive effect. Red: combinations that have a greater additive effect, blue: drugs with antagonistic effects (cancel one another out). Inner wheel of circle shows mean additive effect of a drug across all cell lines, red for drug combinations with overall additive effect, blue for antagonistic combinations. Clusters I, II, III and V, and the most effective combinations within them are highlighted. Chord diagrams illustrate the various drug combinations within a cluster. Radar plots show absolute levels of proliferation (green) and death (red) after drug treatment compared to baseline (grey) for specific drug combinations outlined in black in chord diagrams. **B**) Violin plot showing additive score as predicted by the model (x-axis, see Methods) versus HSA measured experimentally by Nair et al. (2022)^32^ (y-axis). Significance (Welch Two Sample t-test) illustrated through dotted lines from drug combinations that are not predicted to be additive.

To find further treatment combinations that are effective specifically on cancer cells we looked at different combinations of targeted therapies that could increase the difference in death between healthy and cancer cells. Combination treatments are also likely to reduce resistance compared to monotherapy^56^. We performed *in silico* screens of pairwise combinations of 95 druggable targets in the model for SW1573, NCIH1792, HCC827, PC9, A549 and NCIH1993 cell lines (see Methods). We isolated the treatment combinations that were predicted to have an additive effect on overall survival (proliferation - death) when compared to the most effective single treatment (see Methods and Supplementary Figure S11). This was measured for survival rather than death because combination therapy seeks to limit oncogenic growth as well as induce apoptosis, as opposed to radiotherapy which has the primary aim of causing apoptosis or DNA-damage associated death^57^. We then clustered these treatments into those that have a similar effect on the cell lines to highlight patterns of response and profiles of sensitivity. This produced 24 different subclusters, labelled *I - XXIV*, in order of their efficacy vs the most effective single treatment (see Methods, full summary of these clusters in Supplementary Data S2). We see that clusters *I, II* and *IV* are the only broad-spectrum treatments that have a wide, additive effect across all cell lines tested (Figure 5, Methods).

We next compared our simulated results to experimental data generated by Nair and colleagues measuring the additive score of various drug combinations in SW1573, NCIH1792, HCC827, PC9, A549 and NCIH1993 (Supplementary Figure S12, Supplementary Table S7)^32^. We see that the data shows significant concordance with our predictions across all cell lines tested. Comparing Higher than Single Agent effect (HSA) to our predictions of additivity, we see moderate, but significant correlation (Kendall Tau = 0.16; p-value = 8.176e-11) across all drug combination predictions. Given the large proportion of intermediate or no additivity predicted by our model and measured by Nair and colleagues, we also looked at correlation of purely additive combinations as predicted by our model (those shown in Figure 2). In these groups we see stronger correlation (Kendall Tau = 0.27; p-value = 1.7e-07). Correlations of the most additive treatments in Clusters *I - V* show stronger correlation still (Kendall Tau = 0.37; p-value = 2.3e-07). For reference, the average Pearson’s correlation across experimental replicates in GDSC^58^, CTD^59^ and PRISM^60^ drug screening platforms is ~0.30^61^. Although correlation coefficients derived from distinct statistical tests are not directly comparable in magnitude, the observed concordance and statistical significance indicate that our qualitative model captures trends of comparable reproducibility to those observed between independent experimental datasets. We also find that the model’s predictions can recapitulate the sensitivities of different cell lines to specific drugs (See Supplementary Figure S12, S13). These results indicate that our predictions correlate with published data and correlation improves when discriminating between more additive therapies. According to the simulations, the most effective group of drug combinations by average additivity across cell lines are those targeting AKT and MAPK pathways (Cluster I). These have a uniform effect across cancer cell lines, increasing death from the untreated (baseline) level while also causing growth arrest (Figure 5). This combination does not discriminate between oncogenic or healthy cells, so although it is a reliable means of treating a wide range of mutational profiles in cancer, it is also likely cytotoxic to healthy tissue. Cluster II discriminates between healthy cells and tumorigenic cells and is composed of ATR and MYC inhibition. It limits growth to be on par (proliferation = 1) with healthy cells, while increasing death only in cancer cell lines. The remaining highly additive clusters are those dealing with RTKs mutated in specific cancer cell lines. For example, Cluster *III* is additive in cell lines with *EGFR* gain-of-function mutations, while Cluster *V* is additive in NCIH1993, a cell line with a *MET* mutation. These clusters illustrate the importance of cell line-specific key genetic determinants of response that govern the most effective and additive combinations needed for efficacious treatment. However, given drugs within Cluster II are broadly effective regardless of mutational profile, while sparing healthy cells, we decided to explore further the mechanism of action of this line of therapy.

### Combined MYC and ATR inhibition is predicted to limit cell growth and kill cancer cells through double stranded breaks

**Figure 6:**
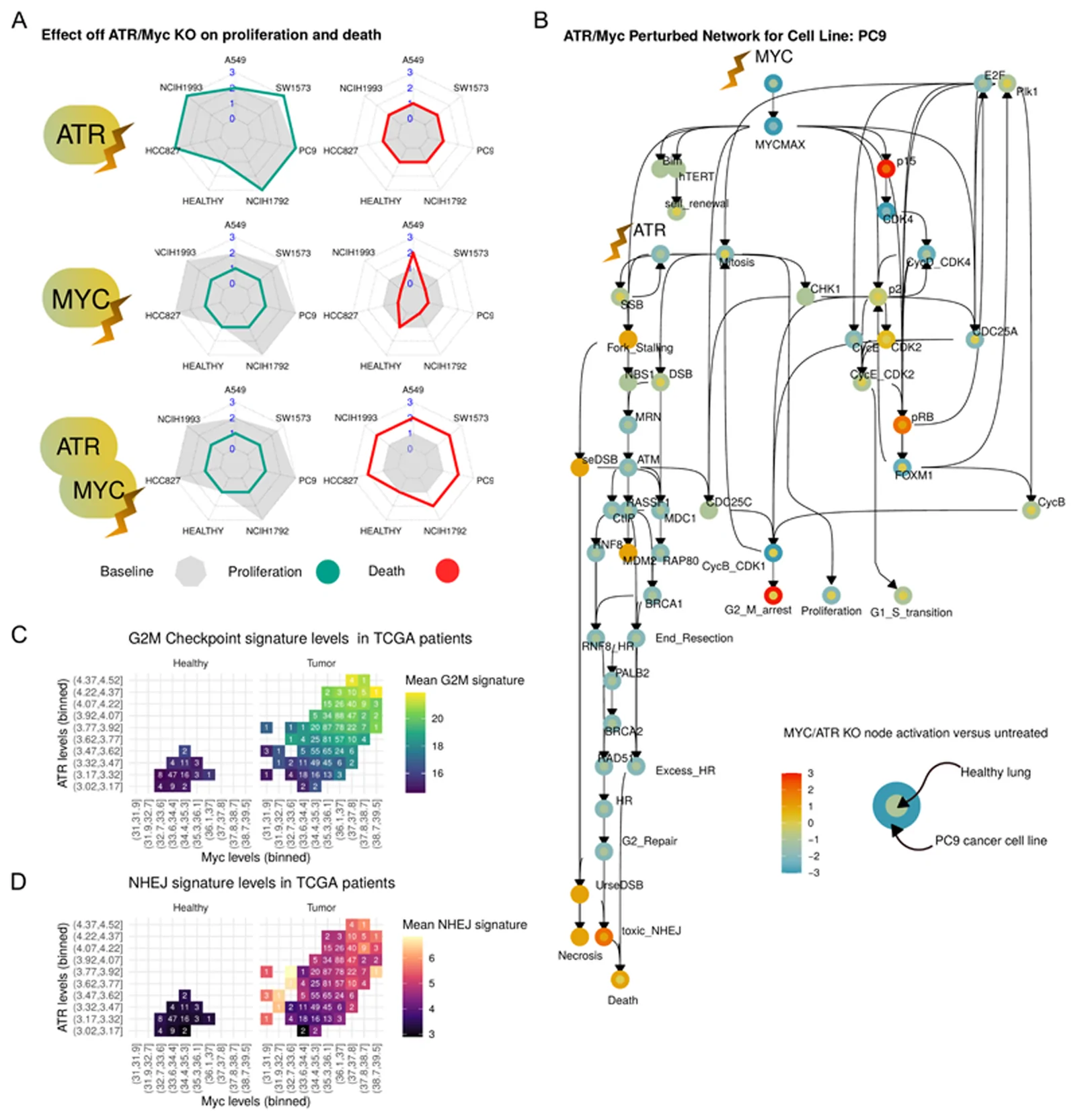
Mechanistic analysis of combined ATR and MYC inhibition illustrates their induction of DNA damage associated death. **A)** Radar plots showing absolute levels of proliferation (green) and apoptosis (red) after MYC inhibition, ATR inhibition and combination thereof. Baseline growth and proliferation shown in grey. **B)** Subnetwork of the model, covering proteins and variables that changed after treatment with MYC/ATR inhibitors in cancer cell line PC9. Node color shows the difference between ATR/MYC inhibition vs untreated (blue: decreased activation, red: increased) in PC9 (outer circle) and healthy lung cells (inner circle). **C)** Heatmaps showing pathway signature deciles in healthy and tumor lung tissue in TCGA NSCLC patients. X-axis shows combined expression of genes associated with Myc activation. Y-axis shows genes associated with the activity of ATR (Methods). Color represents mean normalized enrichment scores for G2/M checkpoint activation (a proliferation proxy), overlaid numbers show sample counts per bin. **D)** As in C but referring to a signature representing the activity of Non-Homologous End Joining (NHEJ).

The model predicts that inhibiting MYC limits proliferation and causes cell death when combined with ATR inhibition in cancer cell lines without affecting healthy cells. This is in line with our earlier prediction of the effect of MYC inhibition in combination with radiotherapy. ATR inhibition alone is predicted to have little impact on all cell lines tested, whereas MYC and ATR inhibitions are predicted to increase cell death in cancer cell lines more than in healthy cells (Figure 6A). To assess the effect of these perturbations on the entire model state, we used Principal Component Analysis (PCA) to visualize the entire state space of the model following treatment with ATR and MYC inhibitors (Supplementary Figure S14), seeing that the treatment predicts to affect healthy cells (bottom left) less, while leading to a large difference in cancer cell lines (top). The loadings for different model variables are also plotted as vectors to highlight those that contribute to the separation in coordinate space of the PCA and represent variables that are changing mostly after perturbation. We predict that following treatment there is an increase in p15, G2/M arrest and toxic NHEJ, which is responsible for the increase in cell death. The variables and the regulatory interactions that exist between them are plotted in Figure 6B. This suggests how ATR and MYC inhibitions combine to limit oncogenic signaling, as well as the induction of DNA damage-associated death in cell line PC9 (the remaining cell lines in Supplementary Figure S14). MYC inhibition is predicted to disrupt the oncogenic operation of the cell cycle, while also inhibiting apoptosis and homologous recombination. Inhibiting ATR could then increase single-ended double strand breaks (seDSB) by preventing the cell from responding properly to replication stress, which is higher in cancer cells due to their higher rate of proliferation. MYC inhibition prevents proper repair of seDSB owing to the loss of MRN and thus ATM activation, leading to toxic NHEJ^50^. Healthy cells are also predicted to be less affected by the inhibition of MYC, which is more highly activated in every cancer cell line (Figure 5C). As a result, inhibition of MYC and ATR is predicted to have the greatest therapeutic window of all the treatments we simulate and could be a promising therapeutic candidate applicable to a wider variety of mutational backgrounds.

To investigate this predicted interaction between MYC and ATR signaling in patient data, we stratified TCGA NSCLC patients based on their inferred MYC and ATR pathway activity scores (see Methods). Using gene-set enrichment analysis of processes represented by nodes identified in Figure 6B, we showed how varied activity of MYC and ATR pathways is associated with progression through the cell division cycle (a proxy for proliferation) and NHEJ (Figure 6C-D). In tumor samples, we see G2/M progression is decreased markedly with lower MYC and ATR activity, but no such relationship occurs in healthy cells. NHEJ has a weaker relationship, but with a subset of low-MYC/ATR tumors displaying an increase in this mode of DNA-damage repair. By quantifying these relationships with a linear model (see Methods), we see that low ATR and MYC is associated with minimal cell cycle progression, with either pathway alone being sufficient to elevate checkpoint signaling. For NHEJ, baseline levels of tumor signature activity are higher than healthy (*B* = 0.852, *p* < 2e-16). Low activities of MYC and ATR together slightly increase NHEJ (*B* = 0.131, *p* = 0.065), even though the individual main effects of MYC and ATR are weakly negative (Supplementary S14). These results suggest that low MYC and ATR activities (analogous to their inhibition) will reduce proliferation and simultaneously may increase NHEJ activity, meaning DNA repair by this more error-prone pathway will potentially be over-activated.

As of writing, MYC inhibitors are in early stage trials^62^, however, other specific drug combinations that we predict to be additive are composed of clinically approved drugs. Unlike MYC and ATR inhibition, these treatments are only effective in selective cell lines. To explore this effect more systematically, we investigated the key explanatory mutations in cell lines that drive responses to various groups of targeted therapy combinations that we predicted as being additive.

### EGFR and TP53 mutations explain additive response to combined MAPK and AKT inhibition

**Figure 7:**
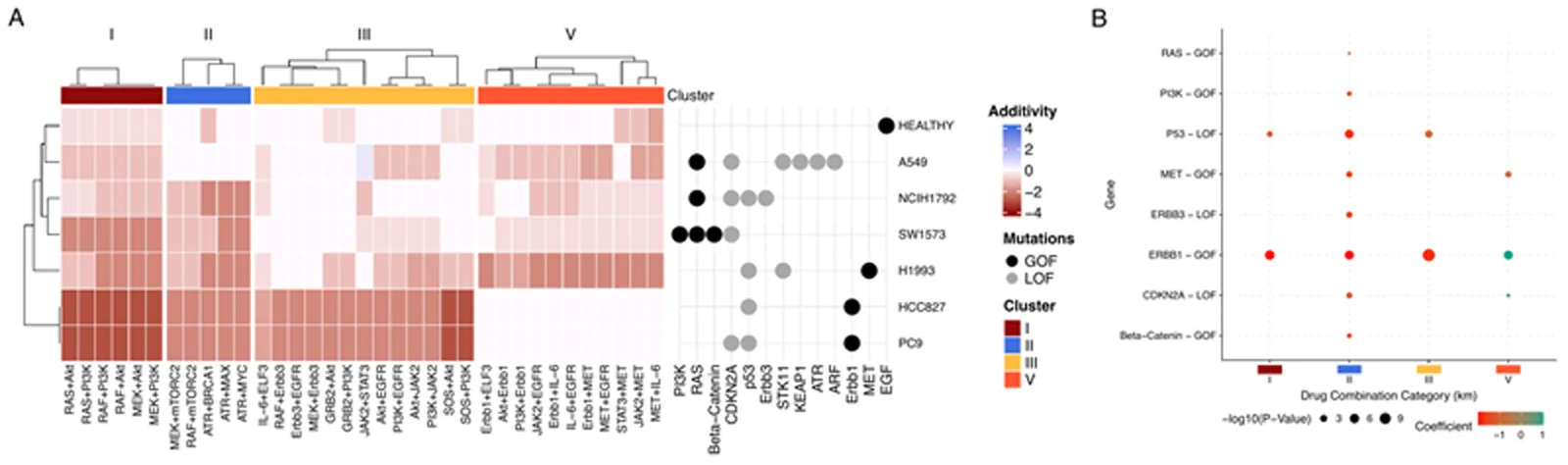
EGFR and TP53 mutations explain the response of cell lines to targeted combination therapy. **A)** Heatmap aligning the mutations of cell lines (right) with response to the most effective drug combination clusters from Figure 5 (red: I, blue: II, yellow: III, orange: V). Columns indicate specific drug treatments; rows indicate cell lines. Heatmap colors show additivity (red: strong additivity, white: none, see Methods). Schematic (right) shows mutations (columns) and cell lines (rows) (black: gain-of-function mutations, grey: loss-of-function). **B)** Linear model coefficients relating cell line mutations (y-axis) to additive drug cluster responses (x-axis). Blue indicates mutation presence associates with additive response; red indicates absence associates with additive response. Point size represents significance.

Since treatment effectiveness varied with small changes in mutational profile, we aimed to find which mutations have the greatest explanatory power in drug response. We visualized drug additivity in the most effective combination clusters alongside the mutations used to model the cell lines (Figure 7A). We predict that EGFR is strongly associated with response to treatments in clusters *I, III* and *V*. For example, cluster *I* treatments (MAPK/Akt inhibition) are most additive in PC9 and HCC827 (EGFR mutant cell lines). Similarly, cluster *III* treatments are most additive in EGFR gain-of-function cases, while cluster *V* shows the opposite. TP53 mutations are associated with response to clusters I, and III with the most additive responders to these being NCIH1993, HCC827 and PC9 (Figure 7A).

We used a linear model to quantify the relationship between cell line mutations and their responses to combination treatments and find the most explanatory mutations for additive drug combinations responses in the different cell lines (Methods). TP53 and EGFR mutations are most broadly associated with additive response to the tested combinations (Figure 3B). However, for Cluster V we see a negative coefficient for EGFR mutations, indicating the absence of this mutation is required to observe additivity. Instead, a MET amplification (as is the case for NCIH1993) is associated with additivity in this cluster (Figure 7B). These results suggest which mutations determine response in different mutational profiles, and highlight EGFR, TP53 and MET as key for patient stratification, out of the 13 driver genes we observe in our panel of cell lines (Figure 7). While it is expected that driver genes are useful in this manner, being able to prioritize which are most associated with difference in response is key to bridging therapeutic responses and oncogenic mutations, allowing us to stratify patients to find the most suitable course of treatment for a given situation.

## Discussion

We developed a computational platform for executable modelling of tumor intrinsic signaling that integrates mechanistic biological knowledge with computational scalability, enabling systematic exploration of the combinatorial therapeutic landscape. Using this platform, we assessed ~66,000 predicted combinations of targeted therapy and radiotherapy, finding the most suitable and effective treatment options, specific to given genotypes. As clinical trials and the development of drugs to take full advantage of the potential of combination treatments will take time, we also investigate methods for better targeting radiotherapy, demonstrate a novel method for stratification of patient sensitivity to radiotherapy using clinical data based on targets identified by the *in silico* model.

We show that this stratification replicates across two independent molecular modalities (RNA expression and CNV), illustrating the model’s generalizability across orthogonal biological signals. For both radiotherapy and combinations of targeted therapy, we predict that MYC has strong potential in targeting cancer cells without affecting healthy cells. MYC is frequently overexpressed in cancer, owing to its central position in the hourglass topology of pathways governing regulation and effect^63^. This importance makes resistance to MYC-targeting drugs extremely difficult^64^. This puts MYC in a unique place as a promising therapeutic target with inhibition well tolerated in healthy tissue^65^. However, despite advances in developing inhibitors targeting MYC, such as OMO-103^62^, none are currently clinically approved for treatment and are likely to be less effective compared to the full knockout simulated by the *in silico* model.

Radiotherapy remains a main component of the standard of care for NSCLC adenocarcinoma treatment^1^. We use our model to propose a novel 19-gene signature that, by combining CNV and RNA-expression data, stratifies patients into radiosensitive and radioresistant groups. We suggest this can be used to better target radiotherapy treatment to those patients that are most likely to benefit from it in the clinic. We predict, and validate with CRISPR screening, that inhibition of 53BP1 leads to radio-sensitization in cancer cell lines but not in healthy cells. 53BP1 loss is a common resistance mutation to PARP inhibitors^13^, and thus this may suggest a collateral sensitivity that can be exploited for second line treatments. This demonstrates how our results have relevance to clinical trial design for multiple settings, with this being most relevant in adjuvant, second line therapy, while our radio-sensitization signature is of most use for initial treatment. However, care will be needed to manage the risk of overlap in toxicity between radiation, radio-sensitizing treatments suggested by the model, and existing DNA damaging chemotherapeutics such as platinum agents^66^.

Challenges remain in the use of qualitative network models. Firstly, the development of such models currently requires manual curation of data to inform both the topology (Supplementary Table S1) and the specification of experiments used to verify the network model (Supplementary Table S5) rely on manual curation. For example, this means that it may be possible that a higher QWK of agreement to the specification was possible before validating with unseen experimental data, but that it was unable to be found due to limitations in time. Similarly, this poses barriers to updating the model with new experimental evidence and taking full advantage of large datasets, such as Perturb-Seq data^67^. This also led us to limit the coverage of the network model to focus on driver genes present in the cell lines used in our CRISPR studies, but the ease of updating the model through our open-source tools^68^ will allow future expansion to cover, for example, the effect of ALK driven lung cancers, as well as squamous cell and large cell carcinoma subtypes of NSCLC, and the impact of the tumor microenvironment^11^. Our current framework is optimized for signaling- and radiation-response pathways. Combinations acting primarily through metabolic or epigenetic mechanisms fall outside the model’s scope, representing both a limitation and an opportunity for future model expansion. In order to facilitate this, we separately demonstrate a machine learning method to build and train such networks, enabling more flexible and rapid training, as well as incorporation of information on wider network topology, which demonstrates comparable performance on this problem (bioRxiv:2025.05.16.653408). We are also limited in our ability to model healthy cells, as information on how these develop somatic mutations in adult tissue remains limited, and positive selection of mutation occurs only in a minority^69,70^, though is likely to be complex, and so we are modelling these as genetically identical gives the best prediction of aggregate behavior. However, as the mutational landscape is mapped, these differences can be incorporated in a similar manner to other cell lines we model. Our focus on the stable states of the network precludes modelling dose fractionation. In the future this could be enabled by combining the network model with a mathematical model^14^. This would also allow better modelling of normal tissue toxicity, separating short- and long-term effects. Finally, our model emphasizes single-target perturbations, and consequently it may underestimate synergistic effects arising from poly-pharmacological combinations which have been found to be significantly more synergistic^32^.

In this study we demonstrate how executable modelling enables system-level experiments to be rapidly conducted, building on our prior work^11–18^ and demonstrating validation both with cell line and patient data. In addition, our *in silico* approach allows mechanistic transparency, providing insight into why treatments do and do not work and the biological underpinnings of mutational profile-specific sensitivities. Using mutational data, our executable models can be individualized, paving the way for ‘digital twins’ of tumors for testing hundreds of thousands of therapeutic options. We envisage that executable models have the potential to improve personalized therapy in NSCLC adenocarcinoma patients and help predict and combat the development of resistance to treatment.

## Supplementary Material

1

2

3

4

5

6

7

8

9

10

11

12

13

14

15

16

17

18

19

20

21

22

23

24

25

## Data Availability

The cancer incidence and survival statistics data analyzed in this study were obtained from SEER21 (RRID:SCR_006902) at https://seer.cancer.gov/statfacts/html/lungb.html. The cancer cell line genomic characterization data analyzed in this study were obtained from Cell Model Passports (RRID:SCR_027682) at https://cellmodelpassports.sanger.ac.uk/. The experimental gene dependency data analyzed in this study were obtained from DepMap (RRID:SCR_017655) at https://depmap.sanger.ac.uk/. The clinical and genomic data for lung adenocarcinoma (LUAD) and lung squamous cell carcinoma (LUSC) analyzed in this study were obtained from The Cancer Genome Atlas Program (TCGA) (RRID:SCR_003193) at https://portal.gdc.cancer.gov/ and cBioPortal (RRID:SCR_014555) at https://www.cbioportal.org/. Gene sets of interest, including ATR- and MYC-associated pathways, G2M Checkpoint, DNA repair and NHEJ, analyzed in this study were obtained from MSigDB (RRID:SCR_016863) at http://software.broadinstitute.org/gsea/msigdb/index.jsp. The druggable proteome data analyzed in this study were obtained from the Human Protein Atlas (RRID:SCR_006710) at http://www.proteinatlas.org/. Full RNA-seq count data for TCGA LUAD and LUSC samples analyzed in this study were obtained from Xena (RRID:SCR_018938) at https://xena.ucsc.edu/. All other raw data generated in this study are available upon request from the corresponding author.

## Statement of Significance

A computational framework that simulates thousands of personalized treatment strategies offers a scalable, cost-effective way to tailor therapies and improve outcomes for genetically diverse non-small cell lung cancer patients.

## Code Availability

The code developed and used in this work, including all scripts and auxiliary scripts used to analyze data and generate figures, is available as a Code Ocean capsule (https://codeocean.com/capsule/0298466/tree/v1) and on the Fisher Lab Github repository (https://github.com/jfisher-lab/NSCLC). The NSCLC *in silico* model JSON file (Supplementary Data S4) can be run via the open-source BioModelAnalyzer tool (https://biomodelanalyzer.org).
