# Supplementary material for: Virtual Tumors Enable Prediction of Personalized Therapeutic Combinations for Non-Small Cell Lung Cancer
Source: Cancer Res. Author manuscript; Available in PMC 2026 Aug 11. (PMC7619348; doi:10.1158/0008-5472.CAN-25-2476)
Supplement: Supplementary file 1 [file EMS216192-supplement-10.pdf]

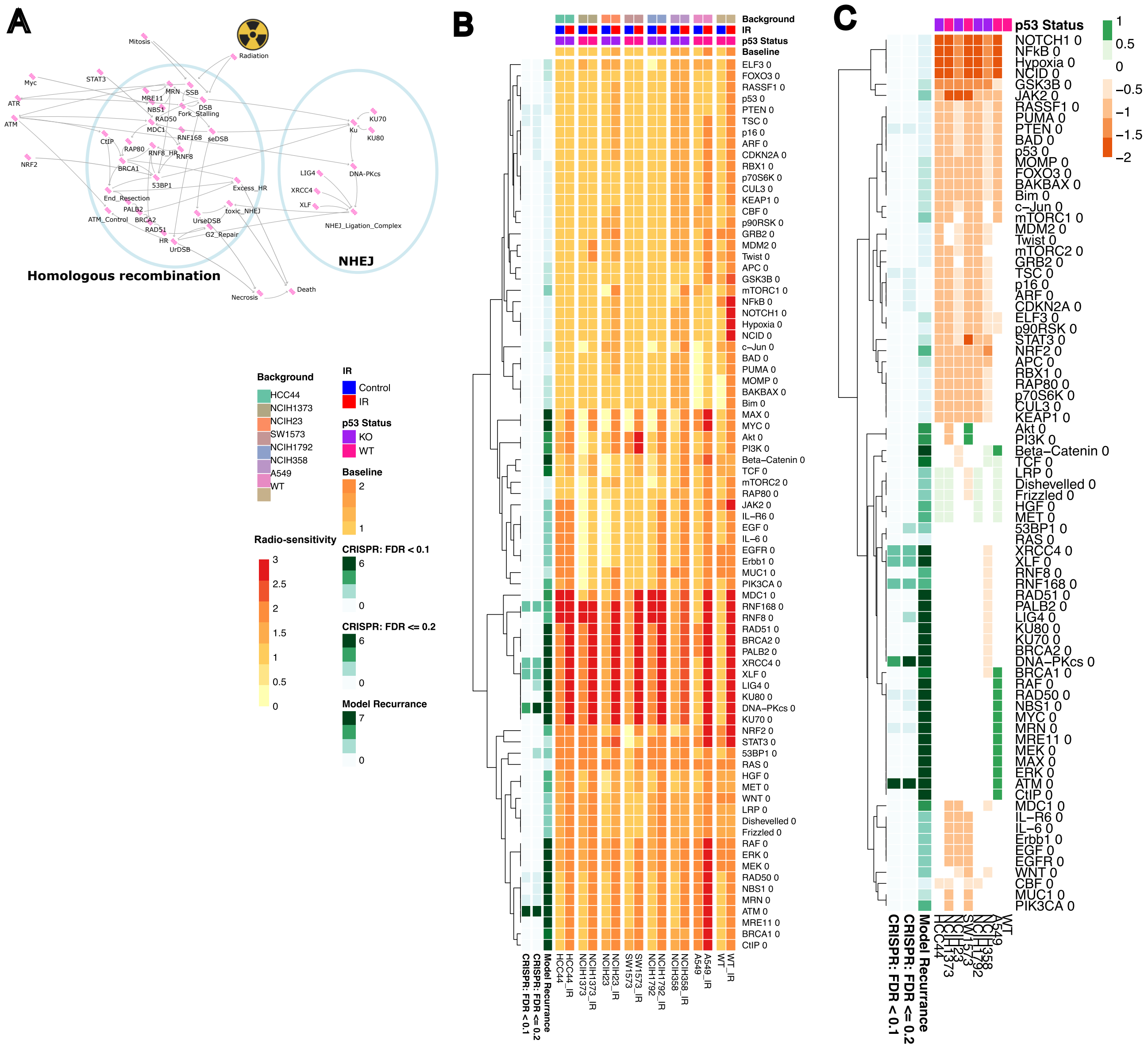

Supplementary Figure S6. Supplementary for predictions pertaining to sensitivity to radiotherapy. A) Schematic showing the part of the model that models irradiation and DNA-damage repair. **B, C)** Effect of gene knock-outs (rows) on different cell line backgrounds (columns). Color represents the activity of the node (cell death **(B)** and therapeutic window **(C)** difference between radiotherapy and knockout in cell line compared to WT cells).
