# Supplementary material for: Virtual Tumors Enable Prediction of Personalized Therapeutic Combinations for Non-Small Cell Lung Cancer
Source: Cancer Res. Author manuscript; Available in PMC 2026 Aug 11. (PMC7619348; doi:10.1158/0008-5472.CAN-25-2476)
Supplement: Supplementary file 2 [file EMS216192-supplement-11.pdf]

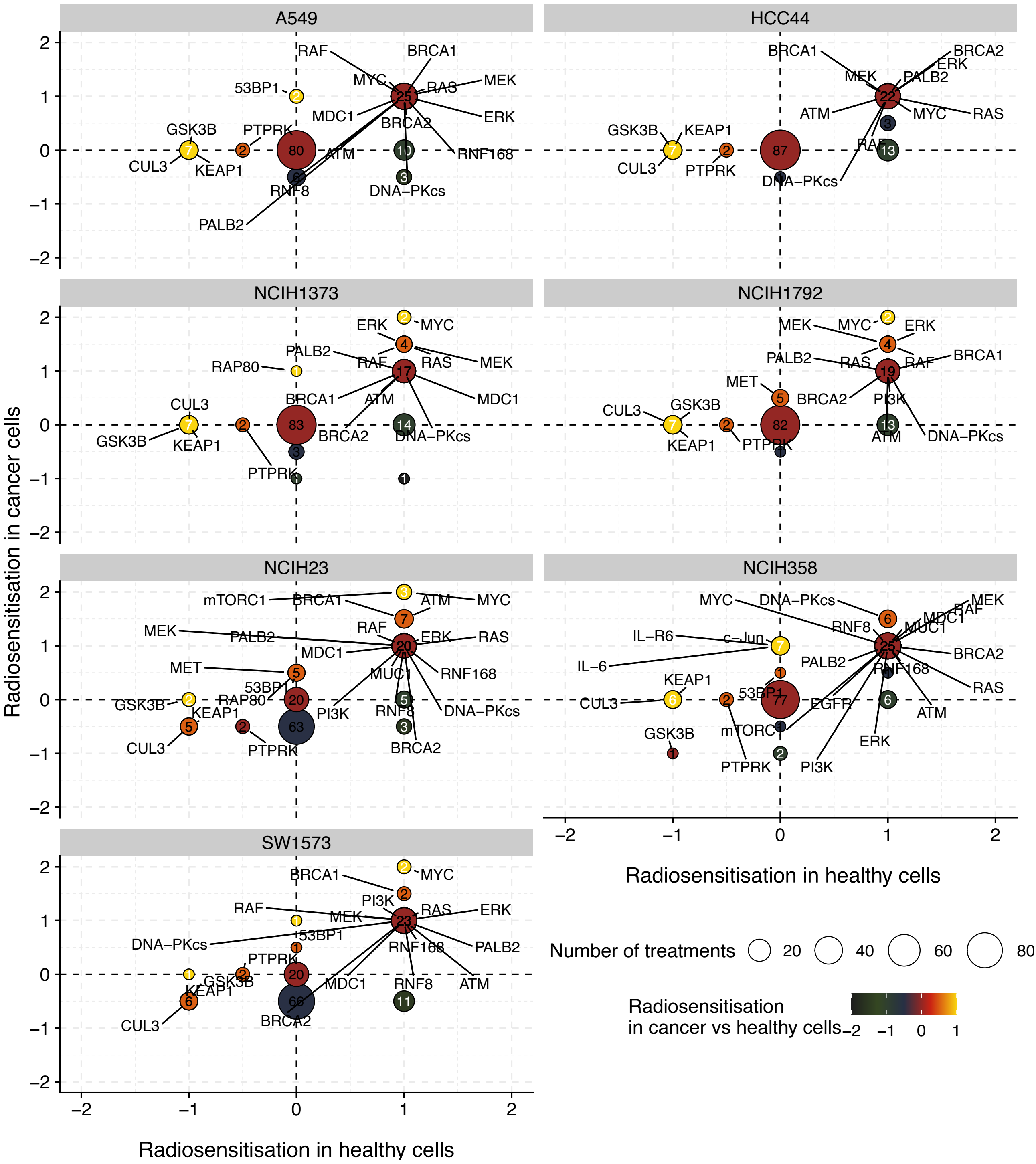

Supplementary Figure S7. Scatter plots showing the difference in DNA-damage associated radio-sensitivity in cancer (y axis) versus healthy cells (x axis). Black is more radio-sensitization of cancer than healthy, yellow is more radio-sensitization of healthy than cancer. Point size is the number of knockouts that have this effect.
