# Supplementary material for: Virtual Tumors Enable Prediction of Personalized Therapeutic Combinations for Non-Small Cell Lung Cancer
Source: Cancer Res. Author manuscript; Available in PMC 2026 Aug 11. (PMC7619348; doi:10.1158/0008-5472.CAN-25-2476)
Supplement: Supplementary file 3 [file EMS216192-supplement-12.pdf]

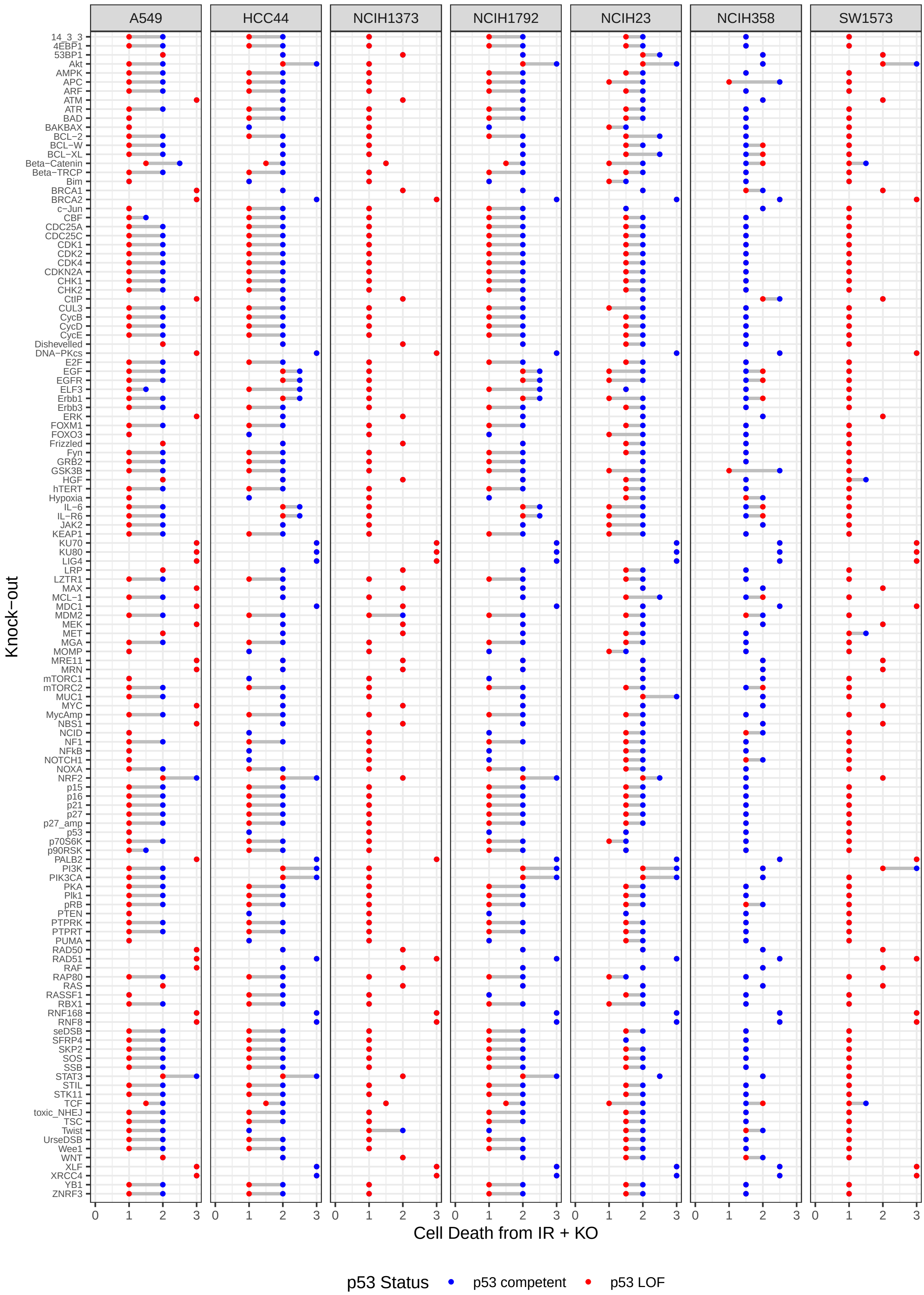

Supplementary Figure S8. The effect of switching p53 status on response to radiotherapy. Number of simulated knockouts leading to each possible level of cell death for p53-competent (blue) and p53-deficient (red) cell lines.
