# Supplementary material for: Virtual Tumors Enable Prediction of Personalized Therapeutic Combinations for Non-Small Cell Lung Cancer
Source: Cancer Res. Author manuscript; Available in PMC 2026 Aug 11. (PMC7619348; doi:10.1158/0008-5472.CAN-25-2476)
Supplement: Supplementary file 4 [file EMS216192-supplement-13.pdf]

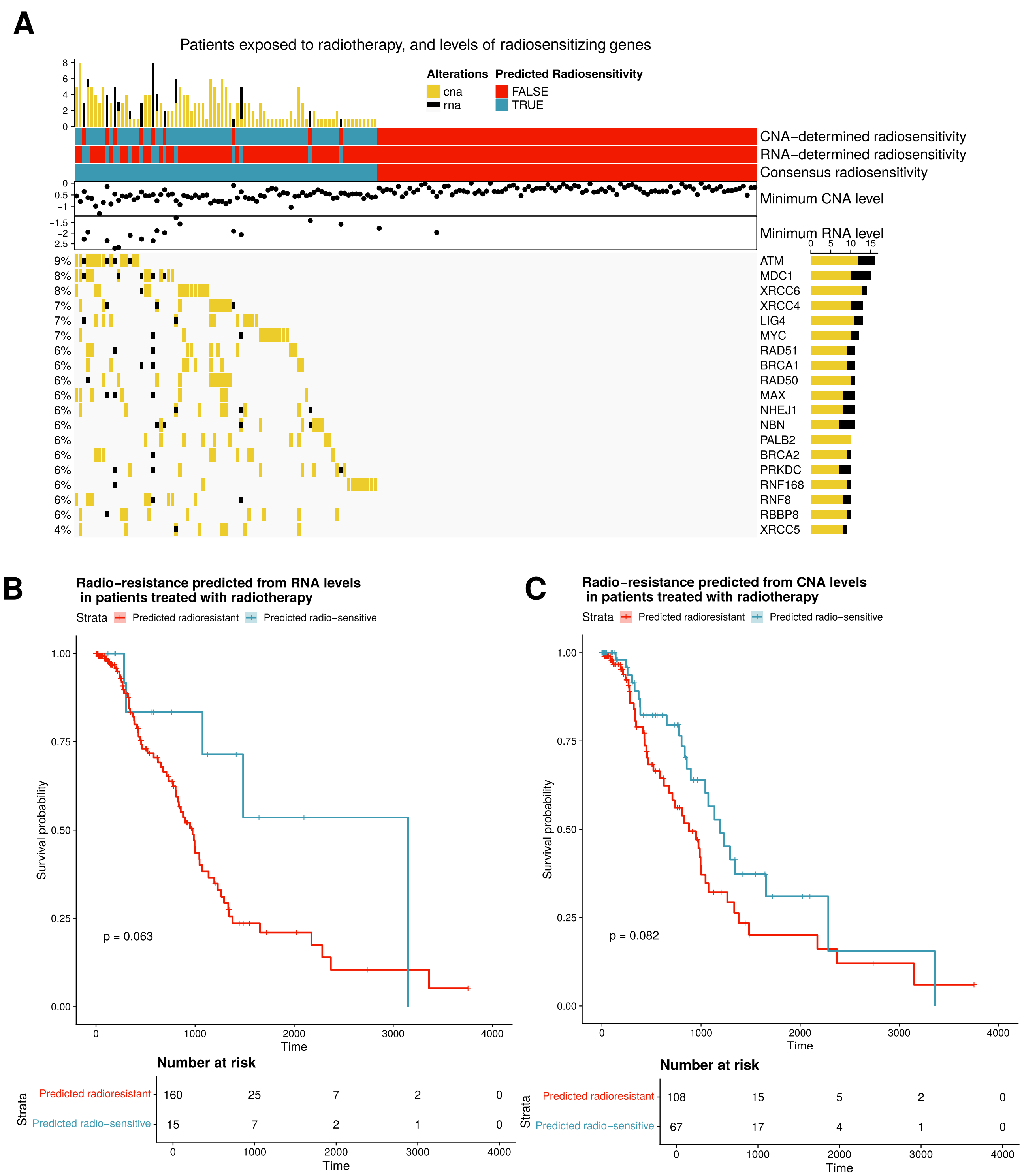

Supplementary Figure S9. ***In silico* predictions stratify radiation-treated patients into radiosensitive and radio-resistant strata. A)** Plot illustrating TCGA data of radiation treated patients (columns) according to the basal activity of predicted radio-sensitizing genes (rows). Cells indicate the presence of low levels of CNV (yellow) or expression (black) for a particular gene. The predicted radio-sensitivity of the patients is marked blue (sensitive) or red (resistant) by the bars on the top. Also shown is the minimum RNA expression and CNV levels for each patient and histograms showing the frequency of alterations for each patient (top) and gene (left). **B, C)** *In silico* predictions stratify radiation-treated patients into radiosensitive and radio-resistant strata. Kaplan-Meier survival curves showing the differences in survival (days) between radio-resistant patients (red) and radiosensitive patients (blue) for patients treated with radiation. These patients were stratified based on their expression levels **(B)** or their copy-number levels **(C)**.
