# Supplementary material for: Virtual Tumors Enable Prediction of Personalized Therapeutic Combinations for Non-Small Cell Lung Cancer
Source: Cancer Res. Author manuscript; Available in PMC 2026 Aug 11. (PMC7619348; doi:10.1158/0008-5472.CAN-25-2476)
Supplement: Supplementary file 5 [file EMS216192-supplement-14.pdf]

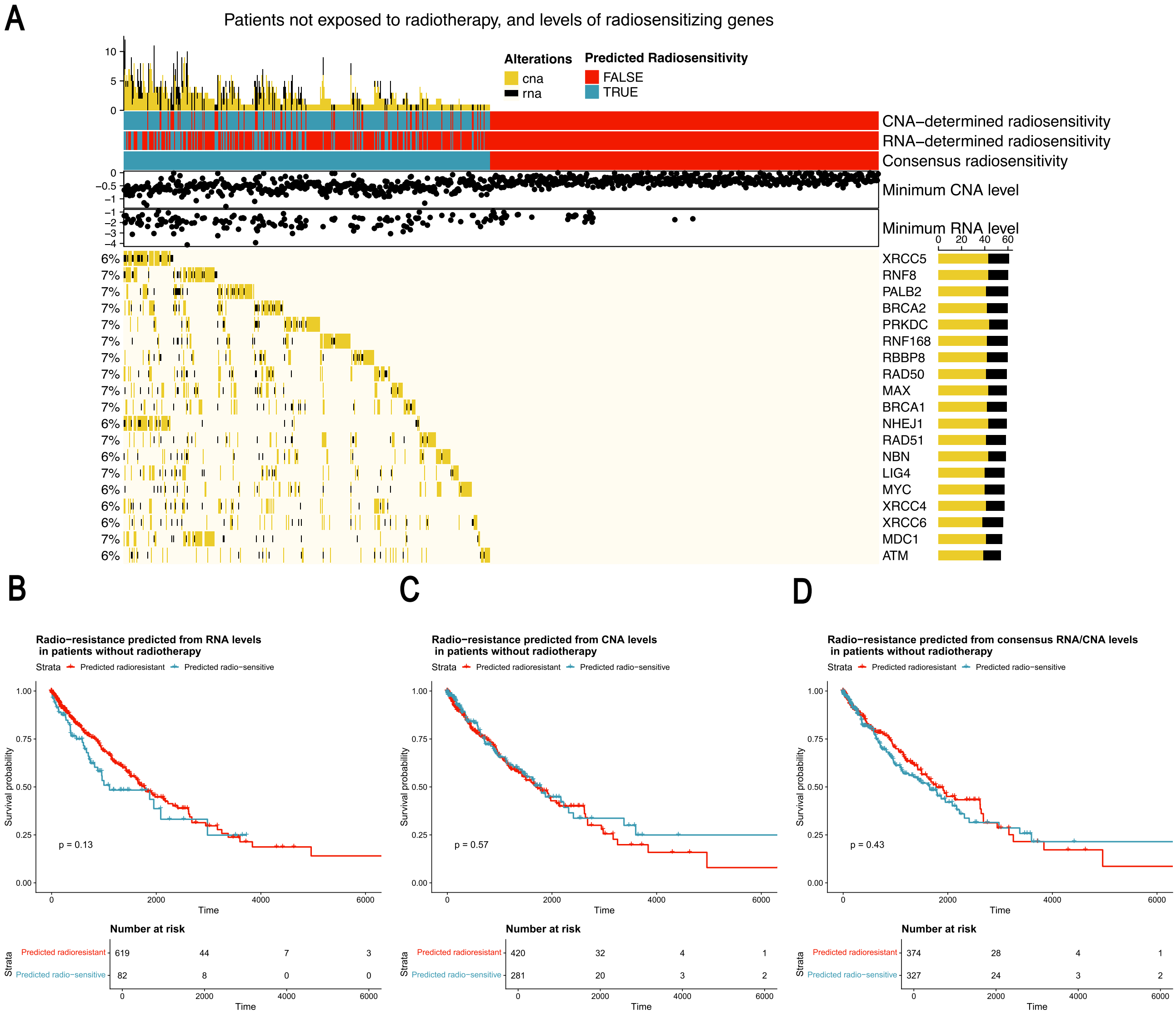

Supplementary Figure S10. *In silico* predictions stratify radiation-treated patients but not treatments that were not treated with radiation. A) Plot illustrating TCGA data of patients not treated with radiotherapy (columns) according to the basal activity of predicted radio-sensitizing genes (rows). Cells indicate the presence of low levels of CNV (yellow) or expression (black) for a particular gene. The predicted radio-sensitivity of the patients is marked blue (sensitive) or red (resistant) by the bars on the top. Also shown is the minimum RNA expression and CNV levels for each patient and histograms showing the frequency of alterations for each patient (top) and gene (left). **B, C, D)** Kaplan-Meier survival curves showing the differences in survival (days) between predicted radio-resistant patients (red) and radiosensitive patients (blue) for patients treated without radiotherapy. These patients were predicted radio-resistant according to expression levels **(B)**, copy number levels **(C)** or a consensus of both **(D)**. The x-axis indicates the time since diagnosis (days) and y-axis indicates the survival probability.
