# Supplementary material for: Virtual Tumors Enable Prediction of Personalized Therapeutic Combinations for Non-Small Cell Lung Cancer
Source: Cancer Res. Author manuscript; Available in PMC 2026 Aug 11. (PMC7619348; doi:10.1158/0008-5472.CAN-25-2476)
Supplement: Supplementary file 6 [file EMS216192-supplement-15.pdf]

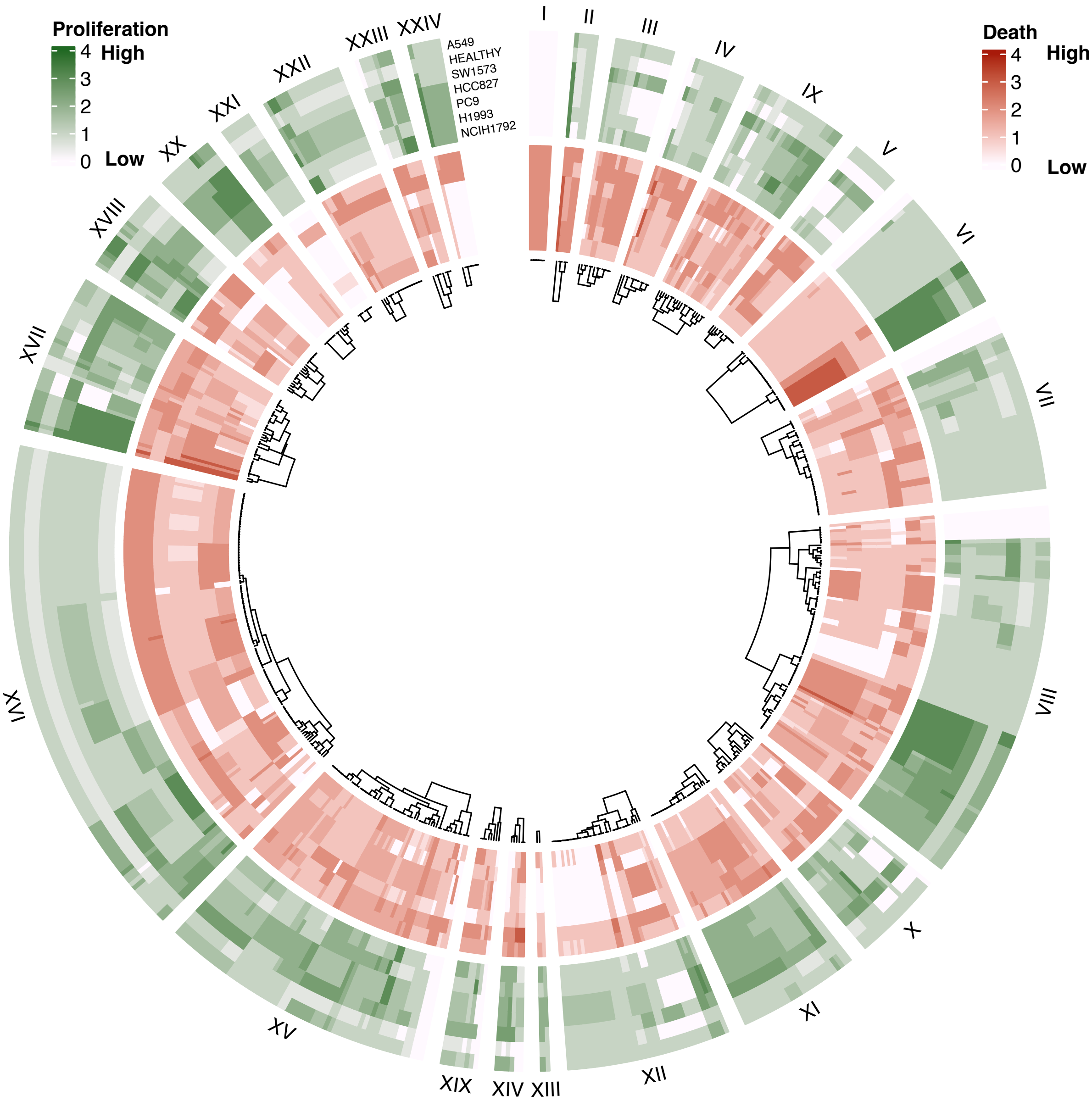

Supplementary Figure S11. Heatmap showing effect of drug combinations on selected NSCLC cell lines. Heatmap showing death (in red, inner circle) and proliferation (in green, outcircle). Columns (spokes of the wheel) are the drug combinations and rows are cell lines. Darker colors indicate higher values for the phenotypes.
