# Supplementary material for: Virtual Tumors Enable Prediction of Personalized Therapeutic Combinations for Non-Small Cell Lung Cancer
Source: Cancer Res. Author manuscript; Available in PMC 2026 Aug 11. (PMC7619348; doi:10.1158/0008-5472.CAN-25-2476)
Supplement: Supplementary file 7 [file EMS216192-supplement-16.pdf]

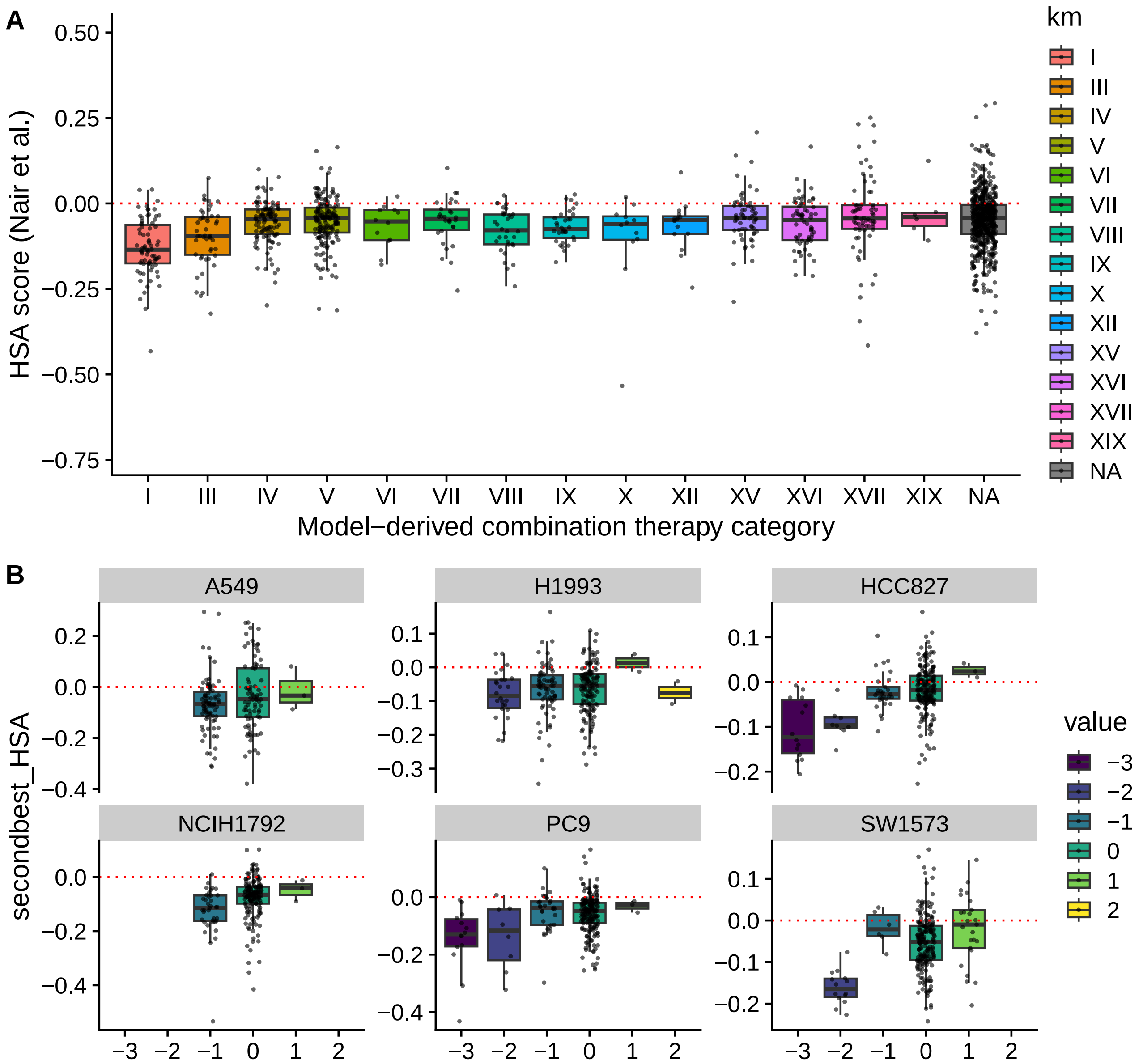

Supplementary Figure S12. Concordance of simulated results for drug combinations with Nair et al. (2023) A) Visualization of predicted additivity (x axis) of drug combinations versus experimentally measured HSA (y axis) for each drug cluster. **B)** Same visualization as panel A, but broken down by cell lines tested by Nair et al. (2023).

Figure References

Nair, N. U., Greninger, P., Zhang, X., Friedman, A. A., Amzallag, A., Cortez, E., Sahu, A. D., Lee, J. S., Dastur, A., Egan, R. K., Murchie, E., Ceribelli, M., Crowther, G. S., Beck, E., McClanaghan, J., Klump-Thomas, C., Boisvert, J. L., Damon, L. J., Wilson, K. M., Ho, J., Tam, A., McKnight, C., Michael, S., Itkin, Z., Garnett, M. J., Engelman, J. A., Haber, D. A., Thomas, C. J., Ruppin, E., and Benes, C. H. (2023). A landscape of response to drug combinations in non-small cell lung cancer. *Nature Communications*, 14(1):3830. Number: 1 Publisher: Nature Publishing Group.
