# Supplementary material for: Virtual Tumors Enable Prediction of Personalized Therapeutic Combinations for Non-Small Cell Lung Cancer
Source: Cancer Res. Author manuscript; Available in PMC 2026 Aug 11. (PMC7619348; doi:10.1158/0008-5472.CAN-25-2476)
Supplement: Supplementary file 8 [file EMS216192-supplement-17.pdf]

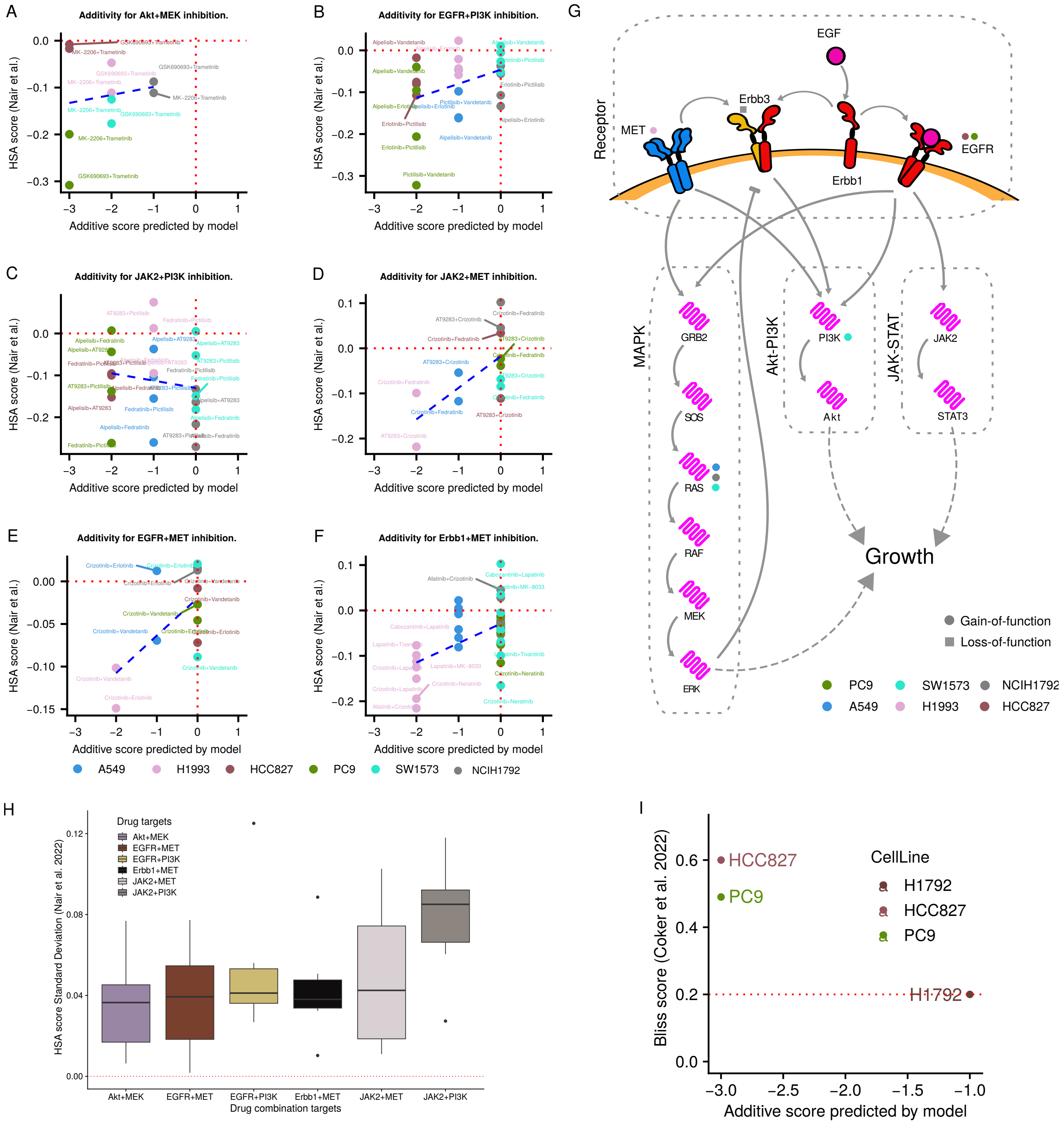

Supplementary Figure S13. Concordance of simulated results with published results for individual drugs. (A-F) Experimentally measured additivity (on the y-axis, HSA from Nair et al. (2023)) compared with the computational predicted value (x-axis) for Akt + MEK inhibition **(A)**, EGFR + PI3K inhibition **(B)**, JAK2 + PI3K inhibition **(C)**, JAK2 + MET inhibition **(D)**, EGFR + MET inhibition **(E)** and Erbb1 + MET inhibition **(F)**. The names of the drugs used are labeled on the points and the colors refers to the cell line with which the reading was taken. Additive score have a positive correlation with the HSA results of Nair et al. (2023) in 5 cases. We observe positive correlation between predicted additive score and HSA in all but one of the drug combinations (inhibition of AKT+MEK, EGFR+PI3K, JAK2+MET and EGFR/Erbb1+MET). JAK2+PI3K is the drug combination where the model prediction does not align with the experimental predictions. **G)** Sub-network of the model illustrating the mutations in the cell lines affecting growth pathways and the regulatory interactions between them. Edges represent regulatory interactions with a pointed arrowhead referring to an activating interaction and a blunt arrowhead indicating inhibitory interactions. Coloured circles indicate where a cell line has a gain-of-function mutation and square circles indicate where they have a low-of-function mutation. **(H)** Box-plot illustrating the standard deviation of additivity (y axis) of different drugs targeting specific proteins (x axis), taken from Nair et al. (2023). JAK2+PI3K inhibition has the highest variance, indicating that the results for JAK2 inhibition and PI3K inhibition are highly dependent on the specific drugs used to target either JAK2 or PI3K. **(I)** Plot comparing the predicted additivity of the model for MAPK+Akt inhibition (x axis) with BLISS score (y axis), taken from Coker et al. (2022). The lower the BLISS score, the more synergistic the drug treatment is. Coker et al. (2022), measured synergy between these two drugs in HCC827 found strong synergy (Bliss score of 0.6), showing high additivity, thus agreeing with our predictions and disagreeing with the findings in Nair et al. (2023).

Figure References

Coker, E. A., Stewart, A., Ozer, B., Minchom, A., Pickard, L., Ruddle, R., Carreira, S., Popat, S., O'Brien, M., Raynaud, F., de Bono, J., Al-Lazikani, B., and Banerji, U. (2022). Individualized Prediction of Drug Response and Rational Combination Therapy in NSCLC Using Artificial Intelligence–Enabled Studies of Acute Phosphoproteomic Changes. *Molecular Cancer Therapeutics*, 21(6):1020–1029.

Nair, N. U., Greninger, P., Zhang, X., Friedman, A. A., Amzallag, A., Cortez, E., Sahu, A. D., Lee, J. S., Dastur, A., Egan, R. K., Murchie, E., Ceribelli, M., Crowther, G. S., Beck, E., McClanaghan, J., Klump-Thomas, C., Boisvert, J. L., Damon, L. J., Wilson, K. M., Ho, J., Tam, A., McKnight, C., Michael, S., Itkin, Z., Garnett, M. J., Engelman, J. A., Haber, D. A., Thomas, C. J., Ruppin, E., and Benes, C. H. (2023). A landscape of response to drug combinations in non-small cell lung cancer. *Nature Communications*, 14(1):3830. Number: 1 Publisher: Nature Publishing Group.
