# Supplementary material for: Virtual Tumors Enable Prediction of Personalized Therapeutic Combinations for Non-Small Cell Lung Cancer
Source: Cancer Res. Author manuscript; Available in PMC 2026 Aug 11. (PMC7619348; doi:10.1158/0008-5472.CAN-25-2476)
Supplement: Supplementary file 9 [file EMS216192-supplement-18.pdf]

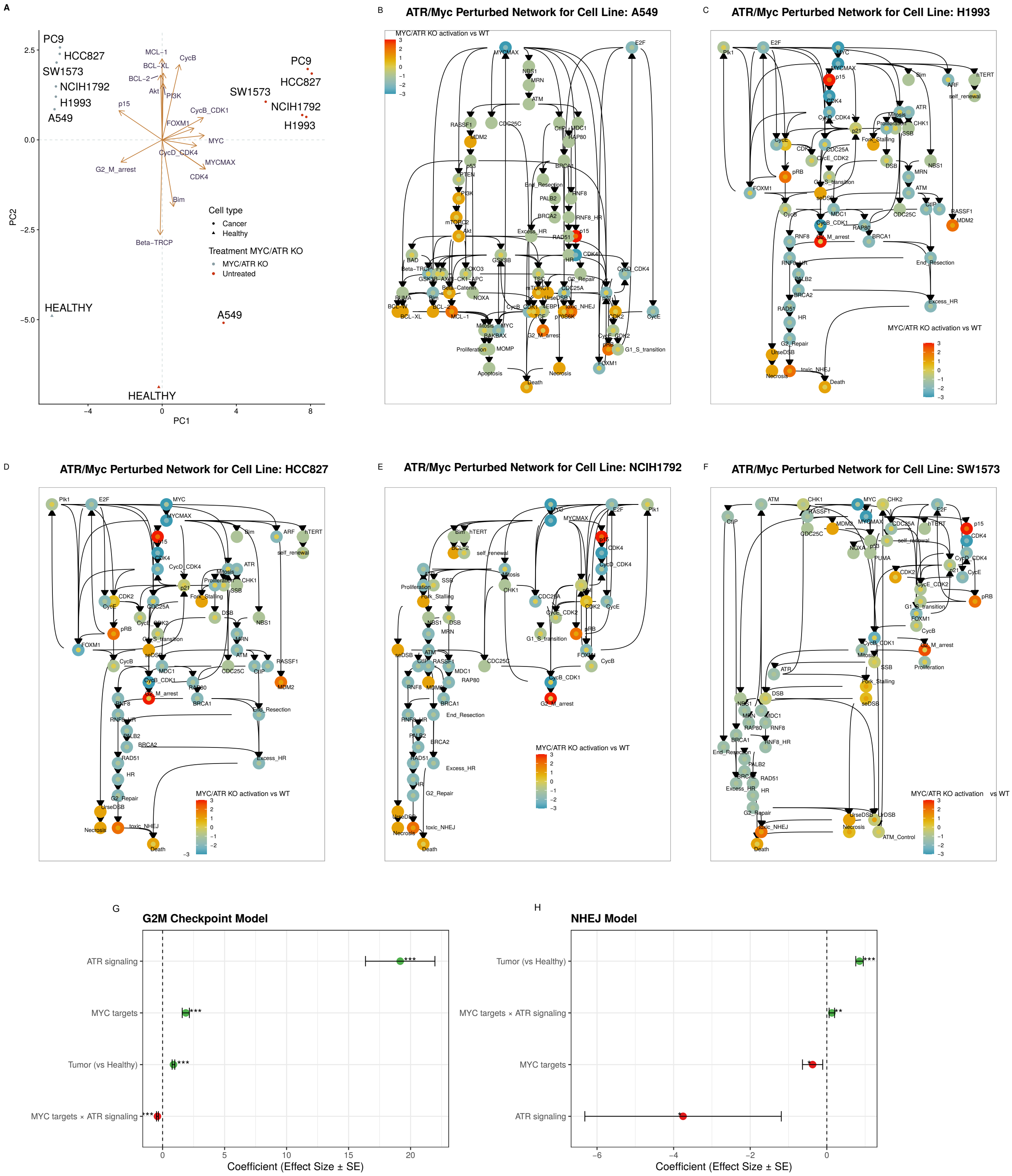

Supplementary Figure S14. Effect of treatment with MYC/ATR KO across genetic contexts. A) PCA showing the model states of different cell lines following treatment with MYC/ATR inhibition. Cancer cell lines are plotted as circles and healthy cells (lacking oncogenic mutations) are triangles. Untreated cells are in red and treated cells are in red. Vectors are also included in the PCA to visualize the loadings of different variables and how they contribute to the separation of *in silico* samples in 2D space. **B-F)** Sub-networks of the model, covering the proteins and variables that changed after treatment of MYC/ATR inhibitors for different cell lines. Color of the node illustrates the difference between ATR/MYC inhibition vs untreated (blue for a relative decrease in activation and red for an increase) in PC9 (outer circle) and the difference in healthy lung cells (inner circle). **G-H)** Dot-and-whisker plots show estimated effect sizes (dots) with standard error bars (whiskers) for predictors in linear models of G2M checkpoint (**G**) and Non-homologous End Joining (NHEJ) (**H**) pathway activity. Predictors include MYC target score, ATR signaling score, sample type (Tumor vs Healthy), and their interactions. They are colored by effect direction, and asterisks denote statistical significance according to the following convention: $p < 0.1$ (*), $p < 0.05$ (**), $p < 0.01$ (***). Dashed lines indicate zero effect.
