# Supplementary material for: Virtual Tumors Enable Prediction of Personalized Therapeutic Combinations for Non-Small Cell Lung Cancer
Source: Cancer Res. Author manuscript; Available in PMC 2026 Aug 11. (PMC7619348; doi:10.1158/0008-5472.CAN-25-2476)
Supplement: Supplementary file 10 [file EMS216192-supplement-19.pdf]

Supplementary Table S1. Regulatory edges in the *in silico* model.

From	To	Type	Reference
14_3_3	CDC25C	Inhibitor	Graves et al. 2001
53BP1	End_Resection	Inhibitor	Zimmermann et al. 2013
53BP1	Ku	Activator	Adams and Carpenter 2006
53BP1	MRN	Activator	Lee et al. 2010
53BP1	RNF8_HR	Inhibitor	Nakada et al. 2012
Akt	AMPK	Inhibitor	Hahn-Windgassen et al. 2005
Akt	Bim	Inhibitor	Qi et al. 2006
Akt	c-Jun	Activator	Go et al. 2001
Akt	Caspase9	Inhibitor	Cardone et al. 1998
Akt	hTERT	Activator	Daniel et al. 2012
Akt	MCL-1	Activator	Fennell and Swanton 2012
Akt	NFkB	Activator	Kucuksayan et al. 2016
Akt	NRF2	Activator	Kang et al. 2008; Wang et al. 2008
Akt	TSC	Inhibitor	Vicary and Roman 2016
APC	GSK3B-AXIN-CK1-APC	Activator	Zhang and Shay 2017
Apoptosis	Death	Activator	Roos and Kaina 2006
ATM	ATM_Control	Activator	Buscemi et al. 2004
ATM	CDC25C	Inhibitor	Thanasoula et al. 2012
ATM	CtIP	Activator	Han et al. 2021
ATM	DNA-PKcs	Activator	Chen et al. 2007
ATM	MDC1	Activator	Lou et al. 2006
ATM_Control	CHK2	Activator	Buscemi et al. 2004
ATR	Fork_Stalling	Inhibitor	Paulsen and Cimprich 2007
Beta-Catenin	hTERT	Activator	Pestana et al. 2017
Beta-Catenin	TCF	Activator	Uematsu et al. 2003
Beta-TRCP	Beta-Catenin	Inhibitor	Ougolkov et al. 2004
BRCA1	53BP1	Inhibitor	Feng et al. 2015
BRCA1	End_Resection	Activator	Cruz-García et al. 2014
BRCA1	RNF8_HR	Inhibitor	Nakada et al. 2012
BRCA2	RAD51	Activator	Luo et al. 2016
c-Jun	BCL-2	Inhibitor	Park et al. 1997
c-Jun	MYC	Activator	Noguchi et al. 1999
Caspase3	Apoptosis	Activator	Naniche et al. 2011
CDC25C	CycB_CDK1	Activator	Liu et al. 2020
CDK1	CycB_CDK1	Activator	Maddika et al. 2007
CDK2	CycE_CDK2	Activator	Gudas et al. 1999
CDK4	CycD_CDK4	Activator	Maddika et al. 2007
CDK4	TSC	Inhibitor	Romero-Pozuelo et al. 2020
CDKN2A	ARF	Activator	Sherr 2001
CDKN2A	p16	Activator	Chan et al. 2021
CHK1	CDC25C	Inhibitor	Uto et al. 2004
CHK2	CDC25C	Inhibitor	Uto et al. 2004
CtIP	BRCA1	Activator	Yun and Hiom 2009
CtIP	End_Resection	Activator	Cruz-García et al. 2014
CUL3	KEAP1-CUL3-RBX1	Activator	Thu et al. 2011
CUL3	LZTR1	Activator	Bigenzahn et al. 2018
CycB	CycB_CDK1	Activator	Maddika et al. 2007
CycD	CycD_CDK4	Activator	Satyanarayana and Kaldis 2009
CycD_CDK4	p21	Inhibitor	Abukhdeir and Park 2008
CycD_CDK4	p27	Inhibitor	James et al. 2008
CycE	CycE_CDK2	Activator	Gudas et al. 1999
CycE_CDK2	G1_S_transition	Activator	Bertoli et al. 2013
DNA-PKcs	NHEJ Ligation Complex	Activator	Davis et al. 2014
DSB	Ku	Activator	Trenner and Sartori 2019

From	To	Type	Reference
DSB	MRN	Activator	Lamarche et al. 2010
DSB	UrDSB	Activator	Waterman et al. 2020
E2F	CycD	Activator	Guo et al. 2011
E2F	CycE	Activator	Hwang and Clurman 2005
EGF	Erbb1	Activator	Pai et al. 1999; Jones et al. 1999
EGFR	Beta-Catenin	Activator	Nakayama et al. 2014
EGFR	GRB2	Activator	Lowenstein et al. 1992
EGFR	IL-6	Activator	Ray et al. 2018
EGFR	JAK2	Activator	Olayioye et al. 1999
EGFR	PI3K	Activator	Fumarola et al. 2014
EGFR	PKA	Activator	Caldwell et al. 2012
ELF3	MEK	Activator	Chen et al. 2019
ELF3	PI3K	Activator	Wang et al. 2018
End_Resection	PALB2	Activator	Nepomuceno et al. 2017
Erbb1	EGFR	Activator	Wieduwilt and Moasser 2008
Erbb1	Erbb3	Activator	Sithanandam and Anderson 2008
Erbb3	PI3K	Activator	Cook et al. 2011
ERK	AMPK	Inhibitor	Hwang et al. 2013
ERK	c-Jun	Activator	Leppä et al. 1998
ERK	Caspase9	Inhibitor	Allan et al. 2003
ERK	CycD	Activator	Chambard et al. 2007
ERK	Erbb3	Activator	Lee et al. 2020
ERK	MCL-1	Activator	Wang et al. 2006
ERK	MYC	Activator	Lavoie et al. 2020
ERK	NRF2	Activator	DeNicola et al. 2011
Excess_HR	Death	Activator	Appanah et al. 2020
Fork_Stalling	DSB	Activator	Cortez 2015
FOXM1	p21	Activator	Tan et al. 2010
FOXO3	Bim	Activator	Meng et al. 2010
Fyn	NRF2	Inhibitor	Niture et al. 2011
G2_Repair	UrDSB	Inhibitor	Paulsen et al. 2021
GRB2	MUC1	Activator	Pandey et al. 1995
GSK3B	4EBP1	Inhibitor	Ito et al. 2016
GSK3B	Fyn	Activator	Jain and Jaiswal 2007
GSK3B	GSK3B-AXIN-CK1-APC	Activator	Pongracz and Stockley 2006
GSK3B	MCL-1	Inhibitor	Fennell and Swanton 2012
GSK3B	p21	Inhibitor	Robertson et al. 2018
GSK3B	TSC	Activator	Evangelisti et al. 2020
HGF	MET	Activator	Organ and Tsao 2011
HR	G2_Repair	Activator	Beucher et al. 2009
hTERT	self_renewal	Activator	Leão et al. 2018
IL-6	IL-R6	Activator	Uciechowski and Dempke 2020
IL-R6	JAK2	Activator	Ni et al. 2004
IR	DSB	Activator	Vignard et al. 2013
IR	SSB	Activator	Mahaney et al. 2009
JAK2	GRB2	Activator	Bousoik and Montazeri Aliabadi 2018
JAK2	NFkB	Activator	Funakoshi-Tago 2011
JAK2	PI3K	Activator	Bousoik and Montazeri Aliabadi 2018
KEAP1	KEAP1-CUL3-RBX1	Activator	Thu et al. 2011
KEAP1-CUL3-RBX1	NFkB	Inhibitor	Thu et al. 2011
KEAP1-CUL3-RBX1	NRF2	Inhibitor	Iso et al. 2016
Ku	DNA-PKcs	Activator	Dobbs et al. 2010
KU70	Ku	Activator	Spagnolo et al. 2006
KU80	Ku	Activator	Spagnolo et al. 2006

From	To	Type	Reference
LIG4	NHEJ Ligation Complex	Activator	Ahnesorg et al. 2006
LZTR1	RAS	Inhibitor	Bigenzahn et al. 2018
MAX	MAXMGA	Activator	Baudino and Cleveland 2001
MAX	MYCMAX	Activator	Baudino and Cleveland 2001
MAXMGA	MYCMAX	Inhibitor	Baudino and Cleveland 2001
MDC1	RNF8	Activator	Kolas et al. 2007
MEK	CHK1	Activator	Lee et al. 2017
MET	Erbb3	Activator	Engelman et al. 2007
MET	GRB2	Activator	Sacco and Clague 2015
MET	MUC1	Activator	Singh et al. 2008
MET	PI3K	Activator	Hervieu and Kermorgant 2018
MGA	MAXMGA	Activator	Baudino and Cleveland 2001
Mitosis	DSB	Activator	Haber 2000
Mitosis	Proliferation	Activator	Levine and Holland 2018
MOMP	Apoptosis	Activator	Tait et al. 2010; Tait and Green 2010
MRE11	MRN	Activator	Lamarche et al. 2010
MRN	ATM	Activator	Uziel et al. 2003
MUC1	EGFR	Activator	Piyush et al. 2017
MUC1	GRB2	Activator	Singh and Bandyopadhyay 2007
MUC1	GSK3B	Inhibitor	Huang et al. 2005
MUC1	MET	Inhibitor	Singh et al. 2008
MUC1	NFkB	Activator	Chaturvedi et al. 2011
MUC1	p53	Activator	Singh et al. 2008
MYC	MYCMAX	Activator	Baudino and Cleveland 2001
MYCMAX	ARF	Activator	Zindy et al. 1998
MYCMAX	Bim	Activator	Muthalagu et al. 2014
MYCMAX	CycD	Activator	García-Gutiérrez et al. 2019
MYCMAX	hTERT	Activator	Khattar and Tergaonkar 2017
MYCMAX	NBS1	Activator	Chiang et al. 2003
MYCMAX	p15	Inhibitor	Staller et al. 2001
MYCMAX	PTEN	Activator	Kaur and Cole 2013
NBS1	MRN	Activator	Lamarche et al. 2010
Necrosis	Death	Activator	Golstein and Kroemer 2007
NFkB	c-Jun	Inhibitor	Eliseev et al. 2005
NFkB	CycD	Activator	Hinz et al. 1999
NHEJ Ligation Complex	UrDSB	Inhibitor	Dong et al. 2018
NOXA	MCL-1	Inhibitor	Zhang et al. 2011
NRF2	53BP1	Activator	Burger et al. 2017
NRF2	BCL-2	Activator	Niture and Jaiswal 2012
NRF2	BCL-XL	Activator	Niture and Jaiswal 2013
NRF2	CycB_CDK1	Activator	Murakami and Motohashi 2015
NRF2	CycD_CDK4	Activator	Shah et al. 2018
NRF2	CycE_CDK2	Activator	Kong et al. 2021
p15	CDK4	Inhibitor	Sandhu et al. 1997
p16	CDK4	Inhibitor	Kim and Sharpless 2006
p21	STAT3	Inhibitor	Coqueret and Gascan 2000
p53	ARF	Inhibitor	Zeng et al. 2011
p53	NFkB	Inhibitor	Zhao et al. 2016
p53	p21	Activator	Shamloo and Usluer 2019
p53	PTEN	Activator	Stambolic et al. 2001
p90RSK	NFkB	Activator	Li et al. 2019
p90RSK	TSC	Inhibitor	Saxton and Sabatini 2017
PALB2	BRCA2	Activator	Zhang et al. 2009a,b
PKA	NF1	Inhibitor	Feng et al. 2004
PTPRK	STAT3	Inhibitor	Xu et al. 2019
PTPRK	ZNRF3	Activator	Chang et al. 2020; Kim et al. 2021
PTPRT	STAT3	Inhibitor	Zhang et al. 2007; Siddiquee et al. 2007

From	To	Type	Reference
RAD50	MRN	Activator	Lamarche et al. 2010
RAD51	HR	Activator	Godin et al. 2016
RAF	MEK	Activator	Roberts and Der 2007
RAP80	BRCA1	Activator	Lombardi et al. 2017
RAS	NRF2	Activator	DeNicola et al. 2011
RAS	SKP2	Activator	Lee et al. 2015
RASSF1	MDM2	Inhibitor	Dubois et al. 2019
RBX1	KEAP1-CUL3-RBX1	Activator	Thu et al. 2011
RNF168	RNF8	Activator	Lu and Matunis 2013
RNF8	53BP1	Activator	Lu and Matunis 2013
RNF8	RNF8_HR	Activator	Nakada et al. 2012
RNF8_HR	RAD51	Activator	Nakada et al. 2012
SFRP4	WNT	Inhibitor	Ghoshal and Ghosh 2016
SKP2	STK11	Activator	Lee et al. 2015
SSB	ATR	Activator	Shiotani et al. 2013; Maréchal and Zou 2013
SSB	Fork_Stalling	Activator	Alexander and Orr-Weaver 2016
STAT3	BCL-2	Activator	Weerasinghe et al. 2007
STAT3	BCL-XL	Activator	Weerasinghe et al. 2007
STAT3	CycB	Activator	Zhang et al. 2014
STAT3	FOXM1	Activator	Liao et al. 2018
STAT3	MCL-1	Activator	Weerasinghe et al. 2007
STAT3	MUC1	Activator	Gao et al. 2009, 2026
TCF	CycD	Activator	Klein and Assoian 2008
TCF	MET	Activator	Boon et al. 2002
TCF	MYC	Activator	Dang 2012
TSC	mTORC1	Inhibitor	Jiang et al. 2019
Twist	ARF	Inhibitor	Kwok et al. 2007
Twist	p53	Inhibitor	Piccinin et al. 2012
UrDSB	ATM_Control	Activator	Ismail et al. 2005
UrDSB	Necrosis	Activator	Higuchi 2003
WNT	Twist	Activator	Howe et al. 2003
XRCC4	NHEJ Ligation Complex	Activator	Hentges et al. 2006; Ahnesorg et al. 2006
ZNRF3	Frizzled	Inhibitor	Chang et al. 2020
STAT3	CycD	Activator	Lee et al. 2019
STIL	Plk1	Activator	Castiel et al. 2011

Supplementary Table S1. Regulatory edges in the *in silico* model. Each row describes an edge, originating from the node in the ”from” column and directed to the node in the ”to” column.

Table References

Abukhdeir, A. M. and Park, B. H. (2008). P21 and p27: roles in carcinogenesis and drug resistance. *Expert reviews in molecular medicine*, 10:e19.

Adams, M. M. and Carpenter, P. B. (2006). Tying the loose ends together in dna double strand break repair with 53bp1. *Cell division*, 1:19.

Ahnesorg, P., Smith, P., and Jackson, S. P. (2006). Xlf interacts with the xrcc4-dna ligase iv complex to promote dna nonhomologous end-joining. *Cell*, 124(2):301–13.

Alexander, J. L. and Orr-Weaver, T. L. (2016). Replication fork instability and the consequences of fork collisions from rereplication. *Genes & development*, 30(20):2241–2252.

Allan, L. A., Morrice, N., Brady, S., Magee, G., Pathak, S., and Clarke, P. R. (2003). Inhibition of caspase-9 through phosphorylation at thr 125 by erk mapk. *Nature cell biology*, 5(7):647–54.

Appanah, R., Jones, D., Falquet, B., and Rass, U. (2020). Limiting homologous recombination at stalled replication forks is essential for cell viability: Dna2 to the rescue. *Current genetics*, 66(6):1085–1092.

Baudino, T. A. and Cleveland, J. L. (2001). The max network gone mad. *Molecular and cellular biology*, 21(3):691–702.

Bertoli, C., Skotheim, J. M., and de Bruin, R. A. M. (2013). Control of cell cycle transcription during g1 and s phases. *Nature reviews. Molecular cell biology*, 14(8):518–28.

Beucher, A., Birraux, J., Tchouandong, L., Barton, O., Shibata, A., Conrad, S., Goodarzi, A. A., Krempler, A., Jeggo, P. A., and Löbrich, M. (2009). Atm and artemis promote homologous recombination of radiation-induced dna double-strand breaks in g2. *The EMBO journal*, 28(21):3413–27.

Bigenzahn, J. W., Collu, G. M., Kartnig, F., Pieraks, M., Vladimer, G. I., Heinz, L. X., Sedlyarov, V., Schischlik, F., Fauster, A., Rebsamen, M., Parapatics, K., Blomen, V. A., Müller, A. C., Winter, G. E., Kralovics, R., Brummelkamp, T. R., Mlodzik, M., and Superti-Furga, G. (2018). Lztr1 is a regulator of ras ubiquitination and signaling. *Science (New York, N.Y.)*, 362(6419):1171–1177.

Boon, E. M. J., van der Neut, R., van de Wetering, M., Clevers, H., and Pals, S. T. (2002). Wnt signaling regulates expression of the receptor tyrosine kinase met in colorectal cancer. *Cancer research*, 62(18):5126–8.

Bousoik, E. and Montazeri Aliabadi, H. (2018). "do we know jack" about jak? a closer look at jak/stat signaling pathway. *Frontiers in oncology*, 8:287.

Burger, K., Schlackow, M., Potts, M., Hester, S., Mohammed, S., and Gullerova, M. (2017). Nuclear phosphorylated dicer processes double-stranded rna in response to dna damage. *The Journal of cell biology*, 216(8):2373–2389.

Buscemi, G., Perego, P., Carenini, N., Nakanishi, M., Chessa, L., Chen, J., Khanna, K., and Delia, D. (2004). Activation of atm and chk2 kinases in relation to the amount of dna strand breaks. *Oncogene*, 23(46):7691–700.

Caldwell, G. B., Howe, A. K., Nickl, C. K., Dostmann, W. R., Ballif, B. A., and Deming, P. B. (2012). Direct modulation of the protein kinase a catalytic subunit α by growth factor receptor tyrosine kinases. *Journal of cellular biochemistry*, 113(1):39–48.

Cardone, M. H., Roy, N., Stennicke, H. R., Salvesen, G. S., Franke, T. F., Stanbridge, E., Frisch, S., and Reed, J. C. (1998). Regulation of cell death protease caspase-9 by phosphorylation. *Science (New York, N.Y.)*, 282(5392):1318–21.

Castiel, A., Danieli, M. M., David, A., Moshkovitz, S., Aplan, P. D., Kirsch, I. R., Brandeis, M., Krämer, A., and Izraeli, S. (2011). The Stil protein regulates centrosome integrity and mitosis through suppression of Chfr. *Journal of Cell Science*, 124(4):532–539.

Chambard, J.-C., Lefloch, R., Pouysségur, J., and Lenormand, P. (2007). Erk implication in cell cycle regulation. *Biochimica et biophysica acta*, 1773(8):1299–310.

Chan, S. H., Chiang, J., and Ngeow, J. (2021). Cdkn2a germline alterations and the relevance of genotype-phenotype associations in cancer predisposition. *Hereditary cancer in clinical practice*, 19(1):21.

Chang, L.-S., Kim, M., Glinka, A., Reinhard, C., and Niehrs, C. (2020). The tumor suppressor ptprk promotes znrf3 internalization and is required for wnt inhibition in the spemann organizer. *eLife*, 9.

Chaturvedi, M. M., Sung, B., Yadav, V. R., Kannappan, R., and Aggarwal, B. B. (2011). NF-κB addiction and its role in cancer: 'one size does not fit all'. *Oncogene*, 30(14):1615–1630.

Chen, B. P. C., Uematsu, N., Kobayashi, J., Lerenthal, Y., Krempler, A., Yajima, H., Löbrich, M., Shiloh, Y., and Chen, D. J. (2007). Ataxia telangiectasia mutated (atm) is essential for dna-pkcs phosphorylations at the thr-2609 cluster upon dna double strand break. *The Journal of biological chemistry*, 282(9):6582–7.

Chen, H., Chen, W., Zhang, X., Hu, L., Tang, G., Kong, J., and Wang, Z. (2019). E26 transformation (ets)-specific related transcription factor-3 (elf3) orchestrates a positive feedback loop that constitutively activates the mapk/erk pathway to drive thyroid cancer. *Oncology reports*, 41(1):570–578.

Chiang, Y.-C., Teng, S.-C., Su, Y.-N., Hsieh, F.-J., and Wu, K.-J. (2003). c-myc directly regulates the transcription of the nbs1 gene involved in dna double-strand break repair. *The Journal of biological chemistry*, 278(21):19286–91.

Cook, R. S., Garrett, J. T., Sánchez, V., Stanford, J. C., Young, C., Chakrabarty, A., Rinehart, C., Zhang, Y., Wu, Y., Greenberger, L., Horak, I. D., and Arteaga, C. L. (2011). Erbb3 ablation impairs pi3k/akt-dependent mammary tumorigenesis. *Cancer research*, 71(11):3941–51.

Coqueret, O. and Gascan, H. (2000). Functional interaction of stat3 transcription factor with the cell cycle inhibitor p21waf1/cip1/sdi1. *The Journal of biological chemistry*, 275(25):18794–800.

Cortez, D. (2015). Preventing replication fork collapse to maintain genome integrity. *DNA repair*, 32:149–157.

Cruz-García, A., López-Saavedra, A., and Huertas, P. (2014). Brca1 accelerates ctip-mediated dna-end resection. *Cell reports*, 9(2):451–9.

Dang, C. V. (2012). Myc on the path to cancer. *Cell*, 149(1):22–35.

Daniel, M., Peek, G. W., and Tollefsbol, T. O. (2012). Regulation of the human catalytic subunit of telomerase (htert). *Gene*, 498(2):135–46.

Davis, A. J., Chen, B. P. C., and Chen, D. J. (2014). Dna-pk: a dynamic enzyme in a versatile dsb repair pathway. *DNA repair*, 17:21–9.

DeNicola, G. M., Karreth, F. A., Humpton, T. J., Gopinathan, A., Wei, C., Frese, K., Mangal, D., Yu, K. H., Yeo, C. J., Calhoun, E. S., Scrimieri, F., Winter, J. M., Hruban, R. H., Iacobuzio-Donahue, C., Kern, S. E., Blair, I. A., and Tuveson, D. A. (2011). Oncogene-induced nrf2 transcription promotes ros detoxification and tumorigenesis. *Nature*, 475(7354):106–9.

Dobbs, T. A., Tainer, J. A., and Lees-Miller, S. P. (2010). A structural model for regulation of nhej by dna-pkcs autophosphorylation. *DNA repair*, 9(12):1307–14.

Dong, J., Ren, Y., Zhang, T., Wang, Z., Ling, C. C., Li, G. C., He, F., Wang, C., and Wen, B. (2018). Inactivation of dna-pk by knockdown dna-pkcs or nu7441 impairs non-homologous end-joining of radiation-induced double strand break repair. *Oncology reports*, 39(3):912–920.

Dubois, F., Bergot, E., and Levallet, G. (2019). Cancer and rassf1a/rassf1c, the two faces of janus. *Trends in cancer*, 5(11):662–665.

Eliseev, R. A., Zuscik, M. J., Schwarz, E. M., O'Keefe, R. J., Drissi, H., and Rosier, R. N. (2005). Increased radiation-induced apoptosis of saos2 cells via inhibition of nfkappab: a role for c-jun n-terminal kinase. *Journal of cellular biochemistry*, 96(6):1262–73.

Engelman, J. A., Zejnullahu, K., Mitsudomi, T., Song, Y., Hyland, C., Park, J. O., Lindeman, N., Gale, C.-M., Zhao, X., Christensen, J., Kosaka, T., Holmes, A. J., Rogers, A. M., Cappuzzo, F., Mok, T., Lee, C., Johnson, B. E., Cantley, L. C., and Jänne, P. A. (2007). Met amplification leads to gefitinib resistance in lung cancer by activating erbb3 signaling. *Science (New York, N.Y.)*, 316(5827):1039–43.

Evangelisti, C., Chiarini, F., Paganelli, F., Marmiroli, S., and Martelli, A. M. (2020). Crosstalks of GSK3 signaling with the mTOR network and effects on targeted therapy of cancer. *Biochimica Et Biophysica Acta. Molecular Cell Research*, 1867(4):118635.

Feng, L., Li, N., Li, Y., Wang, J., Gao, M., Wang, W., and Chen, J. (2015). Cell cycle-dependent inhibition of 53bp1 signaling by brca1. *Cell discovery*, 1:15019.

Feng, L., Yunoue, S., Tokuo, H., Ozawa, T., Zhang, D., Patrakitkomjorn, S., Ichimura, T., Saya, H., and Araki, N. (2004). Pka phosphorylation and 14-3-3 interaction regulate the function of neurofibromatosis type i tumor suppressor, neurofibromin. *FEBS letters*, 557(1-3):275–82.

Fennell, D. A. and Swanton, C. (2012). Unlocking pandora's box: personalising cancer cell death in non-small cell lung cancer. *EPMA Journal*, 3(1):6.

Fumarola, C., Bonelli, M. A., Petronini, P. G., and Alfieri, R. R. (2014). Targeting pi3k/akt/mtor pathway in non small cell lung cancer. *Biochemical pharmacology*, 90(3):197–207.

Funakoshi-Tago, M. (2011). [analysis of the mechanism of polycythemia vera by studying jak2 mutant-induced signaling pathway]. *Yakugaku zasshi : Journal of the Pharmaceutical Society of Japan*, 131(8):1183–7.

Gao, J., McConnell, M. J., Yu, B., Li, J., Balko, J. M., Black, E. P., Johnson, J. O., Lloyd, M. C., Altiok, S., and Haura, E. B. (2009). Muc1 is a downstream target of stat3 and regulates lung cancer cell survival and invasion. *International journal of oncology*, 35(2):337–45.

Gao, J., Mcconnell, M. J., Yu, B., Li, J., Balko, J. M., Black, E. P., Johnson, J. O., Lloyd, M. C., Altiok, S., and Haura, E. B. (2026). [expression of concern] muc1 is a downstream target of stat3 and regulates lung cancer cell survival and invasion. *International journal of oncology*, 68(2).

García-Gutiérrez, L., Bretones, G., Molina, E., Arechaga, I., Symonds, C., Acosta, J. C., Blanco, R., Fernández, A., Alonso, L., Sicinski, P., Barbacid, M., Santamaría, D., and León, J. (2019). Myc stimulates cell cycle progression through the activation of cdk1 and phosphorylation of p27. *Scientific reports*, 9(1):18693.

Ghoshal, A. and Ghosh, S. S. (2016). Antagonizing canonical wnt signaling pathway by recombinant human sfrp4 purified from e. coli and its implications in cancer therapy. *Molecular and cellular biochemistry*, 418(1-2):119–35.

Go, Y. M., Boo, Y. C., Park, H., Maland, M. C., Patel, R., Pritchard, K. A., Fujio, Y., Walsh, K., Darley-Usmar, V., and Jo, H. (2001). Protein kinase b/akt activates c-jun nh(2)-terminal kinase by increasing no production in response to shear stress. *Journal of applied physiology (Bethesda, Md. : 1985)*, 91(4):1574–81.

Godin, S. K., Sullivan, M. R., and Bernstein, K. A. (2016). Novel insights into rad51 activity and regulation during homologous recombination and dna replication. *Biochemistry and cell biology = Biochimie et biologie cellulaire*, 94(5):407–418.

Golstein, P. and Kroemer, G. (2007). Cell death by necrosis: towards a molecular definition. *Trends in biochemical sciences*, 32(1):37–43.

Graves, P. R., Lovly, C. M., Uy, G. L., and Piwnica-Worms, H. (2001). Localization of human cdc25c is regulated both by nuclear export and 14-3-3 protein binding. *Oncogene*, 20(15):1839–51.

Gudas, J. M., Payton, M., Thukral, S., Chen, E., Bass, M., Robinson, M. O., and Coats, S. (1999). Cyclin e2, a novel g1 cyclin that binds cdk2 and is aberrantly expressed in human cancers. *Molecular and cellular biology*, 19(1):612–22.

Guo, Z.-Y., Hao, X.-H., Tan, F.-F., Pei, X., Shang, L.-M., Jiang, X.-L., and Yang, F. (2011). The elements of human cyclin d1 promoter and regulation involved. *Clinical epigenetics*, 2(2):63–76.

Haber, J. E. (2000). Partners and pathwaysrepairing a double-strand break. *Trends in genetics : TIG*, 16(6):259–64.

Hahn-Windgassen, A., Nogueira, V., Chen, C.-C., Skeen, J. E., Sonenberg, N., and Hay, N. (2005). Akt activates the mammalian target of rapamycin by regulating cellular atp level and ampk activity. *The Journal of biological chemistry*, 280(37):32081–9.

Han, J., Wan, L., Jiang, G., Cao, L., Xia, F., Tian, T., Zhu, X., Wu, M., Huen, M. S. Y., Wang, Y., Liu, T., and Huang, J. (2021). Atm controls the extent of dna end resection by eliciting sequential posttranslational modifications of ctip. *Proceedings of the National Academy of Sciences of the United States of America*, 118(12).

Hentges, P., Ahnesorg, P., Pitcher, R. S., Bruce, C. K., Kysela, B., Green, A. J., Bianchi, J., Wilson, T. E., Jackson, S. P., and Doherty, A. J. (2006). Evolutionary and functional conservation of the dna non-homologous end-joining protein, xlf/cernunnos. *The Journal of biological chemistry*, 281(49):37517–26.

Hervieu, A. and Kermorgant, S. (2018). The role of pi3k in met driven cancer: A recap. *Frontiers in molecular biosciences*, 5:86.

Higuchi, Y. (2003). Chromosomal dna fragmentation in apoptosis and necrosis induced by oxidative stress. *Biochemical pharmacology*, 66(8):1527–35.

Hinz, M., Krappmann, D., Eichten, A., Heder, A., Scheidereit, C., and Strauss, M. (1999). Nf-kappab function in growth control: regulation of cyclin d1 expression and g0/g1-to-s-phase transition. *Molecular and cellular biology*, 19(4):2690–8.

Howe, L. R., Watanabe, O., Leonard, J., and Brown, A. M. C. (2003). Twist is up-regulated in response to wnt1 and inhibits mouse mammary cell differentiation. *Cancer research*, 63(8):1906–13.

Huang, L., Chen, D., Liu, D., Yin, L., Kharbanda, S., and Kufe, D. (2005). Muc1 oncoprotein blocks glycogen synthase kinase 3beta-mediated phosphorylation and degradation of beta-catenin. *Cancer research*, 65(22):10413–22.

Hwang, H. C. and Clurman, B. E. (2005). Cyclin e in normal and neoplastic cell cycles. *Oncogene*, 24(17):2776–86.

Hwang, S.-L., Jeong, Y.-T., Li, X., Kim, Y. D., Lu, Y., Chang, Y.-C., Lee, I.-K., and Chang, H. W. (2013). Inhibitory cross-talk between the ampk and erk pathways mediates endoplasmic reticulum stress-induced insulin resistance in skeletal muscle. *British journal of pharmacology*, 169(1):69–81.

Ismail, I. H., Nyström, S., Nygren, J., and Hammarsten, O. (2005). Activation of ataxia telangiectasia mutated by dna strand break-inducing agents correlates closely with the number of dna double strand breaks. *The Journal of biological chemistry*, 280(6):4649–55.

Iso, T., Suzuki, T., Baird, L., and Yamamoto, M. (2016). Absolute amounts and status of the nrf2-keap1-cul3 complex within cells. *Molecular and cellular biology*, 36(24):3100–3112.

Ito, H., Ichiyanagi, O., Naito, S., Bilim, V. N., Tomita, Y., Kato, T., Nagaoka, A., and Tsuchiya, N. (2016). Gsk-3 directly regulates phospho-4ebp1 in renal cell carcinoma cell-line: an intrinsic subcellular mechanism for resistance to mtorc1 inhibition. *BMC cancer*, 16:393.

Jain, A. K. and Jaiswal, A. K. (2007). Gsk-3beta acts upstream of fyn kinase in regulation of nuclear export and degradation of nf-e2 related factor 2. *The Journal of biological chemistry*, 282(22):16502–10.

James, M. K., Ray, A., Leznova, D., and Blain, S. W. (2008). Differential modification of p27kip1 controls its cyclin d-cdk4 inhibitory activity. *Molecular and cellular biology*, 28(1):498–510.

Jiang, C., Bi, C., Jiang, X., Tian, T., Huang, X., Wang, C., Fernandez, M. R., Iqbal, J., Chan, W. C., McKeithan, T. W., Lewis, R. E., and Fu, K. (2019). The mir-17 92 cluster activates mtorc1 in mantle cell lymphoma by targeting multiple regulators in the stk11/ampk/tsc/mtor pathway. *British journal of haematology*, 185(3):616–620.

Jones, J. T., Akita, R. W., and Sliwkowski, M. X. (1999). Binding specificities and affinities of egf domains for erbb receptors. *FEBS letters*, 447(2-3):227–31.

Kang, K. A., Kim, J. S., Zhang, R., Piao, M. J., Ko, D. O., Wang, Z. H., Maeng, Y. H., Eun, S. Y., and Hyun, J. W. (2008). Induction of heme oxygenase-1 by plant extract kiom-79 via akt pathway and nf-e2 related factor 2 in pancreatic beta-cells. *Journal of toxicology and environmental health. Part A*, 71(20):1392–9.

Kaur, M. and Cole, M. D. (2013). Myc acts via the pten tumor suppressor to elicit autoregulation and genome-wide gene repression by activation of the ezh2 methyltransferase. *Cancer research*, 73(2):695–705.

Khattar, E. and Tergaonkar, V. (2017). Transcriptional regulation of telomerase reverse transcriptase (tert) by myc. *Frontiers in cell and developmental biology*, 5:1.

Kim, M., Reinhard, C., and Niehrs, C. (2021). A met-ptprk kinase-phosphatase rheostat controls znrf3 and wnt signaling. *eLife*, 10.

Kim, W. Y. and Sharpless, N. E. (2006). The regulation of ink4/arf in cancer and aging. *Cell*, 127(2):265–75.

Klein, E. A. and Assoian, R. K. (2008). Transcriptional regulation of the cyclin d1 gene at a glance. *Journal of cell science*, 121(Pt 23):3853–7.

Kolas, N. K., Chapman, J. R., Nakada, S., Ylanko, J., Chahwan, R., Sweeney, F. D., Panier, S., Mendez, M., Wildenhain, J., Thomson, T. M., Pelletier, L., Jackson, S. P., and Durocher, D. (2007). Orchestration of the dna-damage response by the rnf8 ubiquitin ligase. *Science (New York, N.Y.)*, 318(5856):1637–40.

Kong, Q., Deng, H., Li, C., Wang, X., Shimoda, Y., Tao, S., Kato, K., Zhang, J., Yamanaka, K., and An, Y. (2021). Sustained high expression of nrf2 and its target genes induces dysregulation of cellular proliferation and apoptosis is associated with arsenite-induced malignant transformation of human bronchial epithelial cells. *The Science of the total environment*, 756:143840.

Kucuksayan, H., Ozes, O. N., and Akca, H. (2016). Downregulation of satb2 is critical for induction of epithelial-to-mesenchymal transition and invasion of nsclc cells. *Lung cancer (Amsterdam, Netherlands)*, 98:122–129.

Kwok, W. K., Ling, M.-T., Yuen, H. F., Wong, Y.-C., and Wang, X. (2007). Role of p14arf in twist-mediated senescence in prostate epithelial cells. *Carcinogenesis*, 28(12):2467–75.

Lamarche, B. J., Orazio, N. I., and Weitzman, M. D. (2010). The mrn complex in double-strand break repair and telomere maintenance. *FEBS letters*, 584(17):3682–95.

Lavoie, H., Gagnon, J., and Therrien, M. (2020). Erk signalling: a master regulator of cell behaviour, life and fate. *Nature reviews. Molecular cell biology*, 21(10):607–632.

Leão, R., Apolónio, J. D., Lee, D., Figueiredo, A., Tabori, U., and Castelo-Branco, P. (2018). Mechanisms of human telomerase reverse transcriptase (hTERT) regulation: Clinical impacts in cancer. *Journal of Biomedical Science*, 25(1):22.

Lee, H. H., Jung, J., Moon, A., Kang, H., and Cho, H. (2019). Antitumor and anti-invasive effect of apigenin on human breast carcinoma through suppression of il-6 expression. *International journal of molecular sciences*, 20(13).

Lee, H.-J., Cao, Y., Pham, V., Blackwood, E., Wilson, C., Evangelista, M., Klijn, C., Stokoe, D., and Settleman, J. (2017). Ras-mek signaling mediates a critical chk1-dependent dna damage response in cancer cells. *Molecular cancer therapeutics*, 16(4):694–704.

Lee, J.-H., Goodarzi, A. A., Jeggo, P. A., and Paull, T. T. (2010). 53bp1 promotes atm activity through direct interactions with the mrn complex. *The EMBO journal*, 29(3):574–85.

Lee, L., Ramos-Alvarez, I., Moody, T. W., Mantey, S. A., and Jensen, R. T. (2020). Neuropeptide bombesin receptor activation stimulates growth of lung cancer cells through her3 with a mapk-dependent mechanism. *Biochimica et biophysica acta. Molecular cell research*, 1867(4):118625.

Lee, S.-W., Li, C.-F., Jin, G., Cai, Z., Han, F., Chan, C.-H., Yang, W.-L., Li, B.-K., Rezaeian, A. H., Li, H.-Y., Huang, H.-Y., and Lin, H.-K. (2015). Skp2-dependent ubiquitination and activation of lkb1 is essential for cancer cell survival under energy stress. *Molecular cell*, 57(6):1022–1033.

Leppä, S., Saffrich, R., Ansorge, W., and Bohmann, D. (1998). Differential regulation of c-jun by erk and jnk during pc12 cell differentiation. *The EMBO journal*, 17(15):4404–13.

Levine, M. S. and Holland, A. J. (2018). The impact of mitotic errors on cell proliferation and tumorigenesis. *Genes & development*, 32(9-10):620–638.

Li, Y., He, M., Lin, W., Xiang, Z., Huang, J., Xu, P., Shi, Y., and Wang, H. (2019). Responses of bladder smooth muscle to the stretch go through extracellular signal-regulated kinase (erk)/p90 ribosomal s6 protein kinase (p90rsk)/nuclear factor-κb (nf-κb) pathway. *Neurourology and urodynamics*, 38(6):1504–1516.

Liao, G.-B., Li, X.-Z., Zeng, S., Liu, C., Yang, S.-M., Yang, L., Hu, C.-J., and Bai, J.-Y. (2018). Regulation of the master regulator foxm1 in cancer. *Cell communication and signaling : CCS*, 16(1):57.

Liu, K., Zheng, M., Lu, R., Du, J., Zhao, Q., Li, Z., Li, Y., and Zhang, S. (2020). The role of cdc25c in cell cycle regulation and clinical cancer therapy: a systematic review. *Cancer cell international*, 20:213.

Lombardi, P. M., Matunis, M. J., and Wolberger, C. (2017). Rap80, ubiquitin and sumo in the dna damage response. *Journal of molecular medicine (Berlin, Germany)*, 95(8):799–807.

Lou, Z., Minter-Dykhouse, K., Franco, S., Gostissa, M., Rivera, M. A., Celeste, A., Manis, J. P., van Deursen, J., Nussenzweig, A., Paull, T. T., Alt, F. W., and Chen, J. (2006). Mdc1 maintains genomic stability by participating in the amplification of atm-dependent dna damage signals. *Molecular cell*, 21(2):187–200.

Lowenstein, E. J., Daly, R. J., Batzer, A. G., Li, W., Margolis, B., Lammers, R., Ullrich, A., Skolnik, E. Y., Bar-Sagi, D., and Schlessinger, J. (1992). The sh2 and sh3 domain-containing protein grb2 links receptor tyrosine kinases to ras signaling. *Cell*, 70(3):431–42.

Lu, J. and Matunis, M. J. (2013). A mediator methylation mystery: Jmjd1c demethylates mdc1 to regulate dna repair. *Nature structural & molecular biology*, 20(12):1346–8.

Luo, K., Li, L., Li, Y., Wu, C., Yin, Y., Chen, Y., Deng, M., Nowsheen, S., Yuan, J., and Lou, Z. (2016). A phosphorylation-deubiquitination cascade regulates the brca2-rad51 axis in homologous recombination. *Genes & development*, 30(23):2581–2595.

Maddika, S., Ande, S. R., Panigrahi, S., Paranjothy, T., Weglarczyk, K., Zuse, A., Eshraghi, M., Manda, K. D., Wiechec, E., and Los, M. (2007). Cell survival, cell death and cell cycle pathways are interconnected: implications for cancer therapy. *Drug resistance updates : reviews and commentaries in antimicrobial and anticancer chemotherapy*, 10(1-2):13–29.

Mahaney, B. L., Meek, K., and Lees-Miller, S. P. (2009). Repair of ionizing radiation-induced dna double-strand breaks by non-homologous end-joining. *The Biochemical journal*, 417(3):639–50.

Maréchal, A. and Zou, L. (2013). Dna damage sensing by the atm and atr kinases. *Cold Spring Harbor perspectives in biology*, 5(9).

Meng, J., Fang, B., Liao, Y., Chresta, C. M., Smith, P. D., and Roth, J. A. (2010). Apoptosis induction by mek inhibition in human lung cancer cells is mediated by bim. *PloS one*, 5(9):e13026.

Murakami, S. and Motohashi, H. (2015). Roles of nrf2 in cell proliferation and differentiation. *Free radical biology & medicine*, 88(Pt B):168–178.

Muthalagu, N., Junttila, M. R., Wiese, K. E., Wolf, E., Morton, J., Bauer, B., Evan, G. I., Eilers, M., and Murphy, D. J. (2014). Bim is the primary mediator of myc-induced apoptosis in multiple solid tissues. *Cell reports*, 8(5):1347–53.

Nakada, S., Yonamine, R. M., and Matsuo, K. (2012). Rnf8 regulates assembly of rad51 at dna double-strand breaks in the absence of brca1 and 53bp1. *Cancer research*, 72(19):4974–83.

Nakayama, S., Sng, N., Carretero, J., Welner, R., Hayashi, Y., Yamamoto, M., Tan, A. J., Yamaguchi, N., Yasuda, H., Li, D., Soejima, K., Soo, R. A., Costa, D. B., Wong, K.-K., and Kobayashi, S. S. (2014). β-catenin contributes to lung tumor development induced by egfr mutations. *Cancer research*, 74(20):5891–902.

Naniche, N., Sau, D., and Pasinelli, P. (2011). In vivo and in vitro determination of cell death markers in neurons. *Methods in molecular biology (Clifton, N.J.)*, 793:9–21.

Nepomuceno, T. C., De Gregoriis, G., de Oliveira, F. M. B., Suarez-Kurtz, G., Monteiro, A. N., and Carvalho, M. A. (2017). The role of palb2 in the dna damage response and cancer predisposition. *International journal of molecular sciences*, 18(9).

Ni, C.-W., Hsieh, H.-J., Chao, Y.-J., and Wang, D. L. (2004). Interleukin-6-induced jak2/stat3 signaling pathway in endothelial cells is suppressed by hemodynamic flow. *American journal of physiology. Cell physiology*, 287(3):C771–80.

Niture, S. K., Jain, A. K., Shelton, P. M., and Jaiswal, A. K. (2011). Src subfamily kinases regulate nuclear export and degradation of transcription factor nrf2 to switch off nrf2-mediated antioxidant activation of cytoprotective gene expression. *The Journal of biological chemistry*, 286(33):28821–28834.

Niture, S. K. and Jaiswal, A. K. (2012). Nrf2 protein up-regulates antiapoptotic protein bcl-2 and prevents cellular apoptosis. *The Journal of biological chemistry*, 287(13):9873–9886.

Niture, S. K. and Jaiswal, A. K. (2013). Nrf2-induced antiapoptotic bcl-xl protein enhances cell survival and drug resistance. *Free radical biology & medicine*, 57:119–31.

Noguchi, K., Kitanaka, C., Yamana, H., Kokubu, A., Mochizuki, T., and Kuchino, Y. (1999). Regulation of c-myc through phosphorylation at ser-62 and ser-71 by c-jun n-terminal kinase. *The Journal of biological chemistry*, 274(46):32580–7.

Olayioye, M. A., Beuvink, I., Horsch, K., Daly, J. M., and Hynes, N. E. (1999). Erbb receptor-induced activation of stat transcription factors is mediated by src tyrosine kinases. *The Journal of biological chemistry*, 274(24):17209–18.

Organ, S. L. and Tsao, M.-S. (2011). An overview of the c-met signaling pathway. *Therapeutic advances in medical oncology*, 3(1 Suppl):S7–S19.

Ougolkov, A., Zhang, B., Yamashita, K., Bilim, V., Mai, M., Fuchs, S. Y., and Minamoto, T. (2004). Associations among beta-trcp, an e3 ubiquitin ligase receptor, beta-catenin, and nf-kappab in colorectal cancer. *Journal of the National Cancer Institute*, 96(15):1161–70.

Pai, R., Jones, M. K., Tomikawa, M., and Tarnawski, A. S. (1999). Activation of raf-1 during experimental gastric ulcer healing is ras-mediated and protein kinase c-independent. *The American journal of pathology*, 155(5):1759–66.

Pandey, P., Kharbanda, S., and Kufe, D. (1995). Association of the df3/muc1 breast cancer antigen with grb2 and the sos/ras exchange protein. *Cancer research*, 55(18):4000–3.

Park, J., Kim, I., Oh, Y. J., Lee, K., Han, P. L., and Choi, E. J. (1997). Activation of c-jun n-terminal kinase antagonizes an anti-apoptotic action of bcl-2. *The Journal of biological chemistry*, 272(27):16725–8.

Paulsen, R. D. and Cimprich, K. A. (2007). The atr pathway: fine-tuning the fork. *DNA repair*, 6(7):953–66.

Paulsen, T., Malapati, P., Shibata, Y., Wilson, B., Eki, R., Benamar, M., Abbas, T., and Dutta, A. (2021). Microdna levels are dependent on mmej, repressed by c-nhej pathway, and stimulated by dna damage. *Nucleic acids research*, 49(20):11787–11799.

Pestana, A., Vinagre, J., Sobrinho-Simões, M., and Soares, P. (2017). TERT biology and function in cancer: Beyond immortalisation. *Journal of Molecular Endocrinology*, 58(2):R129–R146.

Piccinin, S., Tonin, E., Sessa, S., Demontis, S., Rossi, S., Pecciarini, L., Zanatta, L., Pivetta, F., Grizzo, A., Sonego, M., Rosano, C., Dei Tos, A. P., Doglioni, C., and Maestro, R. (2012). A "twist box" code of p53 inactivation: twist box: p53 interaction promotes p53 degradation. *Cancer cell*, 22(3):404–15.

Piyush, T., Chacko, A. R., Sindrewicz, P., Hilkens, J., Rhodes, J. M., and Yu, L.-G. (2017). Interaction of galectin-3 with muc1 on cell surface promotes egfr dimerization and activation in human epithelial cancer cells. *Cell death and differentiation*, 24(11):1937–1947.

Pongracz, J. E. and Stockley, R. A. (2006). Wnt signalling in lung development and diseases. *Respiratory Research*, 7(1):15.

Qi, X.-J., Wildey, G. M., and Howe, P. H. (2006). Evidence that ser87 of bimel is phosphorylated by akt and regulates bimel apoptotic function. *The Journal of biological chemistry*, 281(2):813–23.

Ray, K., Ujvari, B., Ramana, V., and Donald, J. (2018). Cross-talk between egfr and il-6 drives oncogenic signaling and offers therapeutic opportunities in cancer. *Cytokine & growth factor reviews*, 41:18–27.

Roberts, P. J. and Der, C. J. (2007). Targeting the raf-mek-erk mitogen-activated protein kinase cascade for the treatment of cancer. *Oncogene*, 26(22):3291–310.

Robertson, H., Hayes, J. D., and Sutherland, C. (2018). A partnership with the proteasome; the destructive nature of gsk3. *Biochemical pharmacology*, 147:77–92.

Romero-Pozuelo, J., Figlia, G., Kaya, O., Martin-Villalba, A., and Teleman, A. A. (2020). Cdk4 and cdk6 couple the cell-cycle machinery to cell growth via mtorc1. *Cell reports*, 31(2):107504.

Roos, W. P. and Kaina, B. (2006). Dna damage-induced cell death by apoptosis. *Trends in molecular medicine*, 12(9):440–50.

Sacco, J. J. and Clague, M. J. (2015). Dysregulation of the met pathway in non-small cell lung cancer: implications for drug targeting and resistance. *Translational lung cancer research*, 4(3):242–52.

Sandhu, C., Garbe, J., Bhattacharya, N., Daksis, J., Pan, C. H., Yaswen, P., Koh, J., Slingerland, J. M., and Stampfer, M. R. (1997). Transforming growth factor beta stabilizes p15ink4b protein, increases p15ink4b-cdk4 complexes, and inhibits cyclin d1-cdk4 association in human mammary epithelial cells. *Molecular and cellular biology*, 17(5):2458–67.

Satyanarayana, A. and Kaldis, P. (2009). Mammalian cell-cycle regulation: several cdks, numerous cyclins and diverse compensatory mechanisms. *Oncogene*, 28(33):2925–39.

Saxton, R. A. and Sabatini, D. M. (2017). mtor signaling in growth, metabolism, and disease. *Cell*, 168(6):960–976.

Shah, S. Z. A., Zhao, D., Hussain, T., Sabir, N., Mangi, M. H., and Yang, L. (2018). p62-keap1-nrf2-are pathway: A contentious player for selective targeting of autophagy, oxidative stress and mitochondrial dysfunction in prion diseases. *Frontiers in molecular neuroscience*, 11:310.

Shamloo, B. and Usluer, S. (2019). p21 in cancer research. *Cancers*, 11(8).

Sherr, C. J. (2001). The ink4a/arf network in tumour suppression. *Nature reviews. Molecular cell biology*, 2(10):731–7.

Shiotani, B., Nguyen, H. D., Håkansson, P., Maréchal, A., Tse, A., Tahara, H., and Zou, L. (2013). Two distinct modes of atr activation orchestrated by rad17 and nbs1. *Cell reports*, 3(5):1651–62.

Siddiquee, K. A. Z., Gunning, P. T., Glenn, M., Katt, W. P., Zhang, S., Schrock, C., Sebti, S. M., Jove, R., Hamilton, A. D., and Turkson, J. (2007). An oxazole-based small-molecule stat3 inhibitor modulates stat3 stability and processing and induces antitumor cell effects. *ACS chemical biology*, 2(12):787–98.

Singh, P. K., Behrens, M. E., Eggers, J. P., Cerny, R. L., Bailey, J. M., Shanmugam, K., Gendler, S. J., Bennett, E. P., and Hollingsworth, M. A. (2008). Phosphorylation of muc1 by met modulates interaction with p53 and mmp1 expression. *The Journal of biological chemistry*, 283(40):26985–95.

Singh, R. and Bandyopadhyay, D. (2007). Muc1: a target molecule for cancer therapy. *Cancer biology & therapy*, 6(4):481–6.

Sithanandam, G. and Anderson, L. M. (2008). The erbb3 receptor in cancer and cancer gene therapy. *Cancer gene therapy*, 15(7):413–48.

Spagnolo, L., Rivera-Calzada, A., Pearl, L. H., and Llorca, O. (2006). Three-dimensional structure of the human dna-pkcs/ku70/ku80 complex assembled on dna and its implications for dna dsb repair. *Molecular cell*, 22(4):511–9.

Staller, P., Peukert, K., Kiermaier, A., Seoane, J., Lukas, J., Karsunky, H., Möröy, T., Bartek, J., Massagué, J., Hänel, F., and Eilers, M. (2001). Repression of p15ink4b expression by myc through association with miz-1. *Nature cell biology*, 3(4):392–9.

Stambolic, V., MacPherson, D., Sas, D., Lin, Y., Snow, B., Jang, Y., Benchimol, S., and Mak, T. W. (2001). Regulation of pten transcription by p53. *Molecular cell*, 8(2):317–25.

Tait, S. W. G. and Green, D. R. (2010). Mitochondria and cell death: outer membrane permeabilization and beyond. *Nature reviews. Molecular cell biology*, 11(9):621–32.

Tait, S. W. G., Parsons, M. J., Llambi, F., Bouchier-Hayes, L., Connell, S., Muñoz-Pinedo, C., and Green, D. R. (2010). Resistance to caspase-independent cell death requires persistence of intact mitochondria. *Developmental cell*, 18(5):802–13.

Tan, Y., Chen, Y., Yu, L., Zhu, H., Meng, X., Huang, X., Meng, L., Ding, M., Wang, Z., and Shan, L. (2010). Two-fold elevation of expression of foxm1 transcription factor in mouse embryonic fibroblasts enhances cell cycle checkpoint activity by stimulating p21 and chk1 transcription. *Cell proliferation*, 43(5):494–504.

Thanasoula, M., Escandell, J. M., Suwaki, N., and Tarsounas, M. (2012). Atm/atr checkpoint activation downregulates cdc25c to prevent mitotic entry with uncapped telomeres. *The EMBO journal*, 31(16):3398–410.

Thu, K. L., Pikor, L. A., Chari, R., Wilson, I. M., Macaulay, C. E., English, J. C., Tsao, M.-S., Gazdar, A. F., Lam, S., Lam, W. L., and Lockwood, W. W. (2011). Genetic disruption of keap1/cul3 e3 ubiquitin ligase complex components is a key mechanism of nf-kappab pathway activation in lung cancer. *Journal of thoracic oncology : official publication of the International Association for the Study of Lung Cancer*, 6(9):1521–9.

Trenner, A. and Sartori, A. A. (2019). Harnessing dna double-strand break repair for cancer treatment. *Frontiers in oncology*, 9:1388.

Uciechowski, P. and Dempke, W. C. M. (2020). Interleukin-6: A masterplayer in the cytokine network. *Oncology*, 98(3):131–137.

Uematsu, K., Kanazawa, S., You, L., He, B., Xu, Z., Li, K., Peterlin, B. M., McCormick, F., and Jablons, D. M. (2003). Wnt pathway activation in mesothelioma: evidence of dishevelled overexpression and transcriptional activity of beta-catenin. *Cancer research*, 63(15):4547–51.

Uto, K., Inoue, D., Shimuta, K., Nakajo, N., and Sagata, N. (2004). Chk1, but not chk2, inhibits cdc25 phosphatases by a novel common mechanism. *The EMBO journal*, 23(16):3386–96.

Uziel, T., Lerenthal, Y., Moyal, L., Andegeko, Y., Mittelman, L., and Shiloh, Y. (2003). Requirement of the mrn complex for atm activation by dna damage. *The EMBO journal*, 22(20):5612–21.

Vicary, G. W. and Roman, J. (2016). Targeting the mammalian target of rapamycin in lung cancer. *The American journal of the medical sciences*, 352(5):507–516.

Vignard, J., Mirey, G., and Salles, B. (2013). Ionizing-radiation induced dna double-strand breaks: a direct and indirect lighting up. *Radiotherapy and oncology : journal of the European Society for Therapeutic Radiology and Oncology*, 108(3):362–9.

Wang, C. X., Song, J. H., Song, D. K., Yong, V. W., Shuaib, A., and Hao, C. (2006). Cyclin-dependent kinase-5 prevents neuronal apoptosis through erk-mediated upregulation of bcl-2. *Cell death and differentiation*, 13(7):1203–12.

Wang, H., Yu, Z., Huo, S., Chen, Z., Ou, Z., Mai, J., Ding, S., and Zhang, J. (2018). Overexpression of elf3 facilitates cell growth and metastasis through pi3k/akt and erk signaling pathways in non-small cell lung cancer. *The international journal of biochemistry & cell biology*, 94:98–106.

Wang, L., Chen, Y., Sternberg, P., and Cai, J. (2008). Essential roles of the pi3 kinase/akt pathway in regulating nrf2-dependent antioxidant functions in the rpe. *Investigative ophthalmology & visual science*, 49(4):1671–8.

Waterman, D. P., Haber, J. E., and Smolka, M. B. (2020). Checkpoint responses to dna double-strand breaks. *Annual review of biochemistry*, 89:103–133.

Weerasinghe, P., Garcia, G. E., Zhu, Q., Yuan, P., Feng, L., Mao, L., and Jing, N. (2007). Inhibition of stat3 activation and tumor growth suppression of non-small cell lung cancer by g-quartet oligonucleotides. *International journal of oncology*, 31(1):129–36.

Wieduwilt, M. J. and Moasser, M. M. (2008). The epidermal growth factor receptor family: biology driving targeted therapeutics. *Cellular and molecular life sciences : CMLS*, 65(10):1566–84.

Xu, X., Li, D., Liu, J., Ma, Z., Huang, H., Min, L., Dai, L., and Dong, S. (2019). Downregulation of ptprk promotes cell proliferation and metastasis of nsclc by enhancing stat3 activation. *Analytical cellular pathology (Amsterdam)*, 2019:4265040.

Yun, M. H. and Hiom, K. (2009). Ctip-brca1 modulates the choice of dna double-strand-break repair pathway throughout the cell cycle. *Nature*, 459(7245):460–3.

Zeng, Y., Kotake, Y., Pei, X.-H., Smith, M. D., and Xiong, Y. (2011). p53 binds to and is required for the repression of arf tumor suppressor by hdac and polycomb. *Cancer research*, 71(7):2781–92.

Zhang, F., Fan, Q., Ren, K., and Andreassen, P. R. (2009a). Palb2 functionally connects the breast cancer susceptibility proteins brca1 and brca2. *Molecular cancer research : MCR*, 7(7):1110–8.

Zhang, F., Ma, J., Wu, J., Ye, L., Cai, H., Xia, B., and Yu, X. (2009b). Palb2 links brca1 and brca2 in the dna-damage response. *Current biology : CB*, 19(6):524–9.

Zhang, L., Lopez, H., George, N. M., Liu, X., Pang, X., and Luo, X. (2011). Selective involvement of bh3-only proteins and differential targets of noxa in diverse apoptotic pathways. *Cell death and differentiation*, 18(5):864–73.

Zhang, L. and Shay, J. W. (2017). Multiple roles of apc and its therapeutic implications in colorectal cancer. *Journal of the National Cancer Institute*, 109(8).

Zhang, M., Bian, Z.-G., Zhang, Y., Wang, J.-H., Kan, L., Wang, X., Niu, H.-Y., and He, P. (2014). Cucurbitacin b inhibits proliferation and induces apoptosis via stat3 pathway inhibition in a549 lung cancer cells. *Molecular medicine reports*, 10(6):2905–11.

Zhang, X., Guo, A., Yu, J., Possemato, A., Chen, Y., Zheng, W., Polakiewicz, R. D., Kinzler, K. W., Vogelstein, B., Velculescu, V. E., and Wang, Z. J. (2007). Identification of stat3 as a substrate of receptor protein tyrosine phosphatase t. *Proceedings of the National Academy of Sciences of the United States of America*, 104(10):4060–4.

Zhao, M., Fan, J., Liu, Y., Yu, Y., Xu, J., Wen, Q., Zhang, J., Fu, S., Wang, B., Xiang, L., Feng, J., Wu, J., and Yang, L. (2016). Oncogenic role of the tp53-induced glycolysis and apoptosis regulator in nasopharyngeal carcinoma through nf-κb pathway modulation. *International journal of oncology*, 48(2):756–64.

Zimmermann, M., Lottersberger, F., Buonomo, S. B., Sfeir, A., and de Lange, T. (2013). 53bp1 regulates dsb repair using rif1 to control 5' end resection. *Science (New York, N.Y.)*, 339(6120):700–4.

Zindy, F., Eischen, C. M., Randle, D. H., Kamijo, T., Cleveland, J. L., Sherr, C. J., and Roussel, M. F. (1998). Myc signaling via the arf tumor suppressor regulates p53-dependent apoptosis and immortalization. *Genes & development*, 12(15):2424–33.
