# Supplementary material for: Virtual Tumors Enable Prediction of Personalized Therapeutic Combinations for Non-Small Cell Lung Cancer
Source: Cancer Res. Author manuscript; Available in PMC 2026 Aug 11. (PMC7619348; doi:10.1158/0008-5472.CAN-25-2476)
Supplement: Supplementary file 11 [file EMS216192-supplement-20.pdf]

**Supplementary Table S2. Target functions used in the *in silico* model.**

| Node | Target Function | Reason | Reference |
|---|---|---|---|
| 14 3 3 | avg(pos)-avg(neg) | Sequesters Cdc25c in cytoplasm and inhibits its activity | Qi and Martinez 2003 |
| 4EBP1 | 3-mTORC1 | Inhibted by mTORC1 | Guo et al. 2019 |
| 53BP1 | RNF8-BRCA1 + NRF2 -1 | Activated by RNF8 and NRF2 inhibited by BRCA1 | Hu et al. 2014 |
| Akt | ceil(avg(PI3K , mTORC2)) - max(0 , AMPK-1) | Activated by PI3K and mTORC2 inhibited by AMPK | Guo et al. 2013 |
| AMPK | STK11 - max(ERK , Akt) | Activated by STK11 dowregulated by ERK and or Akt | Thoreen and Sabatini 2005 |
| APC | 1 | Contributes to GSK3B AXIN CK1 APC complex | Xing et al. 2003 |
| Apoptosis | ceil(avg(MOMP , Caspase3)) | Activated by MOMP and Caspase 3 | Tait et al. 2010; Tait and Green 2010 |
| ARF | (max(0 , MYCMAX-2) + max(0 , E2F-2)) * min(1 , CDKN2A) - (Twist-2) | Encoded by CDKN2A Activated by overexpression of MYCMAX and ARF inhibited by Twist | Komori et al. 2005; Zindy et al. 1998 |
| ATM | MRN*max(0 , min(1 , 1-ATM_deficient))+min(1 , ATM_deficient*MRN) | | |
| ATM Control | ATM-(2-(UrDSB)) | ATM is activated by DNA double strand breaks | Buscemi et al. 2004; Ismail et al. 2005 |
| ATM deficient | avg(pos)-avg(neg) | | |
| ATR | avg(pos)-avg(neg) | | Maréchal and Zou 2013 |
| BAD | 3-ceil(avg(Akt , p90RSK)) | Inhibited by Akt and p90RSK | Datta et al. 1997; Hurbin et al. 2005 |
| BAKBAX | 3 - ceil(avg(BCL-2 , BCL-W , BCL-XL , MCL-1)) | Inhibited by BCL 2 BCL W BCL XL and MCL 1 | Leber et al. 2018 |

| Node | Target Function | Reason | Reference |
|---|---|---|---|
| BCL 2 | (2 - ceil(avg(BAD , Bim , PUMA)) + min(NFkB , min(NRF2 , STAT3)) - max(0 , 1-c-Jun)) * max(1 , (c-Jun-1)) | Activated by BIM BAD PUMA inhibited by NFkB NRF2 STAT3 and c Jun | Chen et al. 2005; Liang and Fesik 1997 |
| BCL W | 3-floor(avg(Bim , PUMA)) | Inhibited by BIM and PUMA | Adams et al. 2005; Wroblewski et al. 2013; Liu et al. 2014; Gong et al. 2013 |
| BCL XL | 2 - ceil(avg(BAD , Bim , PUMA)) + floor(avg(NFkB , NRF2 , Beta-Catenin , STAT3)) | Activated by NFkB NRF2 Beta Catenin and STAT3 inhibited by BAD Bim and PUMA | Zhang et al. 2018; Adams et al. 2005; Niture and Jaiswal 2013; Li et al. 2013; Chang et al. 2014; Zhang et al. 2013 |
| Beta Catenin | ((2-GSK3B-AXIN-CK1-APC) + Akt + max(0 , EGFR-2)) * (3-Beta-TRCP) | Activated by GSK3B AXIN CK1 APC inhibited by Akt EGFR and Beta TRCP | Zhou et al. 2020; Nakayama et al. 2014 |
| Beta TRCP | 2 * min(max(GSK3B , 0) , 1) | Inhibits Beta Catenin GSK 3 which can phosphorylate TrCP increasing NRF2 ubiquitination | |
| Bim | 3 - ceil(avg(Akt , ERK+1)) + floor(avg(MYCMAX , FOXO3)) | Activated by MYCMAX and FOXO3 inhibited by Akt and ERK | Meng et al. 2010b,a; Takezawa et al. 2011 |
| BRCA1 | max(CtIP , RAP80) * min(1 , CtIP) | Activated by CtIP and RAP80 CtIP function essential for activity | Boichuk et al. 2011 |
| BRCA2 | avg(pos)-avg(neg) | Activated by PALB2 | Zhang et al. 2009a |
| c Jun | floor(avg(Akt , ERK)) * max (1 , (2-NFkB)) | Activated by Akt ERK and NFkB | Nguyen et al. 2004 |
| Caspase3 | avg(pos)-avg(neg) | | |
| Caspase9 | ceil(MOMP - floor(avg(Akt , ERK , CycB_CDK1))) | Activated by MOMP inhibited by Akt ERK and Cyclin B CDK1 | Kalkavan and Green 2018; Cardone et al. 1998; Allan et al. 2003 |
| CBF | 3-NCID | | |

| Node | Target Function | Reason | Reference |
|---|---|---|---|
| CDC25A | ceil(avg(E2F , Plk1 , MYCMAX)) - ceil(avg(CHK1 , CHK2)) | Activated by E2F Plk1 and MYCMAX inhibited by CHK1 and CHK2 | Vigo et al. 1999; Zörnig and Evan 1996 |
| CDC25C | 1+(MYCMAX)-floor(avg(14_3_3 , ATM , CHK1 , CHK2)) | | Karlsson-Rosenthal and Millar 2006; Vigo et al. 1999; Zörnig and Evan 1996; Mailand et al. 2002 |
| CDK1 | 3-Wee1 | Inhibited by Wee1 | |
| CDK2 | 3-avg(p21 , Wee1) | Inhibited by p21 and Wee1 | Al Bitar and Gali-Muhtasib 2019 |
| CDK4 | 3-(max(p15 , p16)) | Inhibited by p15 and p16 | Bockstaele et al. 2006 |
| CDKN2A | 2 | Protein encoding gene | Brastianos et al. 2026; Tong et al. 2021 |
| CHK1 | max(ATR , floor(avg(ATR , MEK-1))) | Activated by ATR Activity potentiated by high MEK activity | Cheng et al. 2017 |
| CHK2 | avg(pos)-avg(neg) | | |
| CtIP | avg(pos)-avg(neg) | Activated by ATM | Han et al. 2021 |
| CUL3 | 2 | Constitutively active unless mutated | Mikac et al. 2021 |
| CycB | ceil(avg(FOXM1 , STAT3)) - FOXO3 | Activated by FOXM1 and STAT3 inhibited by FOXO3 | Li et al. 2012 |
| CycB CDK1 | ceil(avg(CycB , CDK1 , CDC25A , NRF2) - max(0 , p21-2)) * min(1 , CDC25C) * min(1 , CycB) * min(1 , CDK1) * min(1 , CDC25A) | Activated by NRF2 Cyclin B and CDK1 inhibited by p21 | Timofeev et al. 2010; Kreis et al. 2015 |
| CycD | ceil(avg(YB1 , NFkB , TCF , ERK , STAT3)) - floor(avg(GSK3B , 4EBP1)) + MYCMAX | Activated by YB1 NFkB TCF MYCMAX ERK and STAT3 inhibited by GSK3B 4EBP1 | Harada et al. 2014; Hinz et al. 1999; Assoian and Klein 2008; Eeckhoute et al. 2006; García-Gutiérrez et al. 2019; Kang et al. 2019; Barnhart et al. 2008 |

| Node | Target Function | Reason | Reference |
|---|---|---|---|
| CycD CDK4 | floor(avg(CycD , CDK4 , NRF2) - avg(p27 , p21)) * min(1 , min(CycD , CDK4)) | Activated by NRF2 Cyclin D and CDK4 inhibited by p27 and p21 | Datar et al. 2006 |
| CycE | avg(pos)-avg(neg) | | |
| CycE CDK2 | ceil(avg(min(CycE , CDK2) * min(1 , CDC25A) , NRF2)) - (avg(max(0 , p27-1) , max(0 , p21-1))) | Activated by NRF2 Cyclin E CDK2 CDC25A inhibited by p27 and p21 | Gudas et al. 1999; He et al. 2005; Sheaff et al. 1997 |
| Death | (max(max(Apoptosis , Necrosis) , toxic_NHEJ)) + max(0 , min(1 , Excess_HR-1)) | Cell death may be apoptotic or necrotic Excess HR is toxic to the cell | Golstein and Kroemer 2007; Roos and Kaina 2006; Appanah et al. 2020 |
| Dishevelled | avg(Frizzled , LRP) *min(1 , LRP)*min(1 , Frizzled) | Activated by LRP and Frizzled whic are both essential for activity | Cong et al. 2004 |
| DNA PKcs | max(ceil(avg((ATM-1) , Ku)) , Ku) * min(1 , Ku) | Requires presence of Ku for activity degree of activity depends on ATM and Ku activities | Dobbs et al. 2010 |
| DSB | max(max(avg(Mitosis , IR) , avg(Fork_Stalling , IR)) , IR) | IR induces high levels of DSBs and routinely occur during mitosis Fork stalling leads to DNA DSBs | Crasta et al. 2012; Cortez 2015 |
| E2F | 3-pRB | Inhibited by pRB | Giacinti and Giordano 2006 |
| EGF | 2 | Activated by EGFR | Wells 1999 |
| EGFR | Erbb1 * (min(1 , MUC1) * max(1 , (MUC1-1))) | Activated by Erbb1 and MUC1 MUC1 function essential for activity | Kharbanda et al. 2014 |
| ELF3 | 1 | Activated by RAS and PI3K | |
| End Resection | max(BRCA1 , avg(CtIP , BRCA1)-53BP1) | Activated by CtIP and BRCA1 inhibited by 53BP1 | Cruz-García et al. 2014; Zimmermann et al. 2013 |
| Erbb1 | avg(pos)-avg(neg) | | |
| Erbb3 | ((max(0 , (1-ERK))*max(0 , min(1 , Erbb1-2)) + max(0 , (1-Erbb1))*max(0 , min(1 , MET-2))))*3 | Activated by ERK MET and EGFR | Sithanandam and Anderson 2008; Oh et al. 2020 |

| Node | Target Function | Reason | Reference |
|---|---|---|---|
| ERK | MEK | MEK is essential for ERK activity ERK is further activated by MUC1 | |
| Excess HR | min(End_Resection , 3-max(0 , min(1 , max(BRCA1 , BRCA2)))) | | |
| Fork Stalling | SSB-ATR | Replication fork stalling is caused by DNA single strand breaks ATR prevents fork collapse and promotesrestart of replication fork preogression | Alexander and Orr-Weaver 2016; Paulsen and Cimprich 2007 |
| FOXM1 | floor(avg(CycD_CDK4 , E2F , MYCMAX , STAT3)) - pRB | Activated by CyclinD CDK4 E2F MYCMAX and STAT3 inhibited by pRB | Liao et al. 2018 |
| FOXO3 | 3 - ceil(avg(Akt , ERK)) + min(1 , min(AMPK , p53)) | Activated by AMPK and p53 inhibited by Akt and ERK | Renault et al. 2011 |
| Frizzled | WNT-max(0 , ZNRF3-1) | Activated by Wnt inhibited by ZNRF3 | Chang et al. 2020 |
| Fyn | avg(pos)-avg(neg) | | |
| G1 S transition | avg(pos)-avg(neg) | Activated by Cyclin E CDK2 | Bertoli et al. 2013 |
| G1arrest | avg(pos)-avg(neg) | Inhibited by p27 | Park et al. 2001 |
| G2 M arrest | 3 - CycB_CDK1 | Inhibited by CyclinB CDK1 | Castedo et al. 2002b |
| G2 Repair | avg(pos)-avg(neg) | HR and MMEJ mediate DNA double strand break repair in S and G2 phases of the cell cycle | Truong et al. 2013 |
| GRB2 | ceil(avg(EGFR , JAK2 , MET , MUC1)) | Activated by EGFR JAK2 MET and MUC1 | Sacco and Clague 2015 |
| GSK3B | 3 - ceil(avg(Akt , ERK , MUC1 , p70S6K-1 , p90RSK)) | Inhibited by Akt ERK MUC1 p70S6K and p90RSK | |
| GSK3B AXIN CK1 APC | (3-Dishevelled) * min(GSK3B , APC) | Inhibited by Dishevelled APC and GSK3B are key members of this protein complex | Huang and He 2008 |

| Node | Target Function | Reason | Reference |
|---|---|---|---|
| HGF | 2 | Activated by MET | Organ and Tsao 2011 |
| HR | avg(pos)-avg(neg) | Mediates homologous recombination | Cloud et al. 2012 |
| hTERT | ceil(avg(Akt , Beta-Catenin , MYCMAX)) | Activated by Akt Beta Catenin and MYCMAX | Daniel et al. 2012; Khattar and Tergaonkar 2017 |
| Hypoxia | 2 | | |
| IL 6 | 2 + max(0 , EGFR-2) | Constitutive expression High EGFR activity elevates expression | Ray et al. 2018 |
| IL R6 | avg(pos)-avg(neg) | | |
| IR | 3-IR_FLIP | DSBs are induced by IR | Barnard et al. 2013 |
| IR FLIP | 3 | | |
| JAK2 | floor(avg(EGFR , IL-R6)) + max(0 , IL-R6-2) + max(0 , EGFR-2) | Independent activation by EGFR and IL R6 | Wee and Wang 2017 |
| KEAP1 | 2 | Constituively activated unless mutated | Nguyen et al. 2009 |
| KEAP1 CUL3 RBX1 | min(RBX1 , min(CUL3 , KEAP1)) | Ubiquitin ligase complex composed by KEAP1 CUL3 and RBX1 | Thu et al. 2011 |
| Ku | avg(max(DSB , seDSB) , 53BP1) * min(1 , KU70)*min(1 , KU80) | Components of Ku complex are Ku70 and KU80 Activated by DSBs and 53BP1 | |
| KU70 | 1 | Component of the Ku heterodimer | Mari et al. 2006 |
| KU80 | 1 | Component of the Ku heterodimer | Mari et al. 2006 |
| LIG4 | 1 | Component of the NHEJ Ligation Complex | Gu et al. 2007 |
| LRP | avg(pos)-avg(neg) | | |
| LZTR1 | CUL3-1 | Activated by CUL3 | Bigenzahn et al. 2018 |
| MAX | 3 | Constitutively active unless mutated | Kato et al. 1992 |
| MAXMGA | min (MGA , MAX) | Activated by MAX and MGA requires function of both these proteins for activity | Diolaiti et al. 2015 |

| Node | Target Function | Reason | Reference |
|---|---|---|---|
| MCL 1 | 2 - ceil(avg(Bim , NOXA , PUMA , GSK3B)) + min(min(Akt , ERK) , STAT3) | Activated by Akt ERK and STAT3 inhibited by Bim NOXA PUMA and GSK3B | Jin et al. 2011; Cai et al. 2011; Song et al. 2005; Kang et al. 2017; Weerasinghe et al. 2007 |
| MDC1 | max(DSB , seDSB) * min(1 , ATM+max(0 , min(1 , STAT3-2)))*min(1 , ATM) | ATM mediated phosphorylation of H2AX at double strand breaks is essential for MDC1 activation | Eliezer et al. 2014 |
| MDM2 | 3 - ceil(avg(ARF , RASSF1)) | Inhibited by ARF and RASSF1 | Song et al. 2008 |
| MEK | RAF-max(0 , min(1 , 1-ELF3)) | Activated by RAF inhibited by ELF3 | Roberts and Der 2007; Chen et al. 2019 |
| MET | HGF +(min(1 , TCF)-1) | Activated by HGF and TCF inhibited by MUC1 | Raghav et al. 2012; Singh et al. 2008 |
| MGA | 1 | Inhibits MYCMAX through the MAXMGA heterodimer | Baudino and Cleveland 2001 |
| Mitosis | ceil(avg(CycB_CDK1 , Plk1)) | Activated by CyclinB CDK1 and Plk1 | Castedo et al. 2002a; Gheghiani et al. 2017 |
| MOMP | avg(pos)-avg(neg) | | |
| MRE11 | 1 | Component of the MRN complex | Langerak et al. 2011 |
| MRN | min((MRE11) , min (NBS1 , RAD50)) * max(DSB , floor(avg(DSB , 53BP1))) | Composed of MRE11 NBS1 and RAD50 primarily activated by DSBs and also by 53BP1 | Uziel et al. 2003; Lee et al. 2010a,b |
| mTORC1 | 3-TSC | Inhibited by TSC | Jiang et al. 2019 |
| mTORC2 | avg(pos)-avg(neg) | | |
| MUC1 | floor(avg(STAT3 , GRB2 , MET , 1)) | Activated by STAT3 GRB2 and MET | Gao et al. 2009 |
| MYC | ceil(avg(ERK , TCF , c-Jun)) - ceil(avg(GSK3B , 4EBP1))*min(2 , max(1 , MycAmp)) | Activated by ERK STAT3 and B catenin inhibited by FOXO3 p53 GSK3B and 4EBP1 | Barnhart et al. 2008; Noguchi et al. 1999 |
| MycAmp | avg(pos)-avg(neg) | | |

| Node | Target Function | Reason | Reference |
|---|---|---|---|
| MYCMAX | min(MYC , MAX) * min(2 , 3-(MAXMGA)) | Requires both MYC and MAX activity antagonised by MAXMGA | Baudino and Cleveland 2001 |
| NBS1 | 1 *min(1 , MYCMAX) | Component of the MRN complex MYCMAX function essential for activity | Kobayashi et al. 2004; Chiang et al. 2003 |
| NCID | avg(pos)-avg(neg) | The ultimate event in Notch multiple proteolytic processing leads to the release in the nucleus of the activated form of Notch intracellular Notch or NotchIC | |
| Necrosis | max(UrDSB , min(1 , UrseDSB)) | Unrepaired DNA Double Strand Breaks result in Necrotic death | |
| NF1 | 3-PKA | PKA downregulates NF1 expression | Bergoug et al. 2020 |
| NFkB | ceil(avg(Akt , p90RSK , JAK2 , MUC1))-floor(avg(p53 , KEAP1-CUL3-RBX1)) | Activated by Akt p90RSK JAK2 and MUC1 inhibited by p53 and KEAP1 | Zhu et al. 2019 |
| NHEJ Ligation Complex | DNA-PKcs * min(min(1 , LIG4) , min(XRCC4 , XLF)) | Protein complex consists of XRCC4 XLF4 and LIG4 Activated by DNA PKcs | Davis et al. 2014; Ahnesorg et al. 2006 |
| NOTCH1 | avg(pos)-avg(neg) | NOTCH1 only active in NSCLC in hypoxic conditions | |
| NOXA | avg(pos)-avg(neg) | | |

| Node | Target Function | Reason | Reference |
|---|---|---|---|
| NRF2 | 3 - floor(avg(Fyn , KEAP1-CUL3-RBX1*2 , Beta-TRCP)) + floor(avg(ERK , Akt)) | Activated by ERK and Akt inhibited by Fyn and KEAP1 Another regulator of NRF2 is glycogen synthase kinase 3 beta GSK 3 This protein can phosphorylate TrCP increasing the ability of TrCP to ubiquitinate NRF2 | Barrera-Rodríguez 2018 |
| p15 | 3-MYCMAX | Inhibited by MYCMAX | García-Gutiérrez et al. 2019 |
| p16 | CDKN2A - YB1 | Inhibtied by YB1 Encoded by CDKN2A | |
| p21 | ceil(avg(NRF2 , FOXO3 , FOXM1) + p53) - floor(avg(max(MYCMAX-1 , 0) , CycD_CDK4 , Akt , NRF2 , GSK3B)) | Activated by p53 FOXO3 and FOXM1 inhibited by MYCMAX CycD CDK4 Akt NRF2 and GSK3B | Lee et al. 2008; Zhou et al. 2018; Li et al. 2018; García-Gutiérrez et al. 2019; Tan et al. 2010 |
| p27 | (1 + FOXO3 - floor(avg(MYCMAX , Akt , p90RSK , CycE_CDK2 , NRF2))) | Activated by FOXO3 inhibited by MYCMAX Akt p90RSK Cyclin E CDK2 and NF2 | García-Gutiérrez et al. 2019; Sherr and Roberts 1999; Lu et al. 2017 |
| p27 amp | 1 | | |
| p53 | 3 - ceil(avg(MDM2 , Twist , NCID)) + min(1 , (min(min(CHK1 , CHK2) , min(AMPK , MUC1)))) | Activated by AMPK CHK1 CHK2 and MUC1 inhibited by MDM2 and Twist It is inhibited by NOTCH1 through the modulation of its stability at the posttranslational level | Thoreen and Sabatini 2005; Chène 2003; Yang et al. 2005 |
| p70S6K | avg(pos)-avg(neg) | | |
| p90RSK | avg(pos)-avg(neg) | | |
| PALB2 | avg(pos)-avg(neg) | Activated by BRCA1 | Zhang et al. 2009b |

| Node | Target Function | Reason | Reference |
|---|---|---|---|
| PI3K | floor(avg(EGFR , EGFR , MET , JAK2 , RAS , MUC1) - PTEN + Erbb3) * min(1 , PIK3CA) + max(ELF3-1 , 0) | Activated by EGFR MET JAK2 MUC1 IRS4 Erbb3 and RAS and inhibited by PTEN PIK3CA encodes the catalytic subunit of PI3K and its function is essential to the activity of PI3K | Bidkhori et al. 2012; Hervieu and Kermorgant 2018; Bousoik and Montazeri Aliabadi 2018; Shaw and Cantley 2006; Lee et al. 2007; Engelman et al. 2007; Raina et al. 2011; Yang et al. 2025 |
| PIK3CA | 1 | Encodes catalytic subunit of PI3K | Martinez-Martí and Felip 2011 |
| PKA | avg(pos)-avg(neg) | Activated by Erbb1 | |
| Plk1 | 2 -ceil(avg(p53 , FOXO3))+ floor(avg(STIL , FOXM1)) | Activated by FOXM1 and STIL inhibited by p53, FOXO3, p21 | Nestal de Moraes et al. 2016; Alvarez et al. 2001 |
| pRB | 2 - min(CycE_CDK2 , CycD_CDK4) | Inibitied by CycE CDK2 and CycD CDK4 | VanArsdale et al. 2015 |
| Proliferation | avg(pos)-avg(neg) | | |
| PTEN | floor(avg(p53 , MYCMAX)) - floor(avg(CBF , c-Jun)) | Activated by p53 and MYCMAX inhibited by c Jun Notch signalling downregulates the expression of PTEN via the induction of hairy enhancer of split 1 | Stambolic et al. 2001 |
| PTPRK | 2 | Activates ZNRF3 inhibits STAT3 | Chang et al. 2020 |
| PTPRT | 1 | Activated by homodimerisation activates STAT3 | Zhang et al. 2007 |
| PUMA | max(FOXO3 , p53) *min(1 , p53) | Activated by FOXO3 and p53 p53 function essential for activity | Nakano and Vousden 2001; You et al. 2006 |
| RAD50 | 1 | Component of the MRN complex | Dupré et al. 2006 |

| Node | Target Function | Reason | Reference |
|---|---|---|---|
| RAD51 | floor(min(BRCA2 , RNF8_HR)) | Activated by BRCA2 and RNF8 | Esashi et al. 2007 |
| RAD51 | floor(min(BRCA2 , RNF8_HR)) | Activated by BRCA2 and RNF8 | Esashi et al. 2007; Nakada et al. 2012 |
| RAF | avg(pos)-avg(neg) | Ras essential for activity Activity further potentiated by IRS4 | |
| RAP80 | avg(pos)-avg(neg) | Recruited by MDC1 to DNA double strand breaks | Strauss and Goldberg 2011 |
| RAS | SOS - max(0 , (avg(NF1 , LZTR1)-1)) | Activated by SOS inhibited by NF1 | Boriack-Sjodin et al. 1998 |
| RASSF1 | avg(pos)-avg(neg) | Inhibited by DNMT3B | Palakurthy et al. 2009 |
| RBX1 | 2 | Constitutively active unless mutated | Wei and Sun 2010 |
| RNF168 | 1 | Component of the RNF8 RNF168 ubiquitin ligase complex | Chen et al. 2021 |
| RNF8 | MDC1*min(1 , RNF168) | Activated by MDC1 acts in concurrence with RNF168 to promote accumulation of 53BP1 | Huen et al. 2007; Bartocci and Denchi 2013 |
| RNF8 HR | RNF8 - max(0 , 2*(1- min(1 , BRCA1) - min(1 , 53BP1))) | Both BRCA1 and 53BP1 must be absent for RNF8 to promote homologous recombination via RAD51 | Nakada et al. 2012 |
| seDSB | avg(pos)-avg(neg) | | |
| self renewal | avg(pos)-avg(neg) | | |
| Senescence | avg(p21 , p16) *min(1 , p21)*min(1 , p16) | | |
| SFRP4 | 1 | Inhibits Wnt | Park et al. 2008 |
| SKP2 | avg(pos)-avg(neg) | Activated by Ras | Lee et al. 2015 |
| SOS | avg(pos)-avg(neg) | | |
| SSB | max(ceil(avg(Mitosis , IR)) , IR) | Induced by ionising radiation and routinely occurs during mitosis | Mahaney et al. 2009 |

| Node | Target Function | Reason | Reference |
|---|---|---|---|
| STAT3 | ((floor(avg(JAK2 , MET , EGFR)) + max(0 , JAK2-2))) + max(0 , 1-PTPRT) + max(0 , 1-PTPRK) | Activated by JAK2 and MET inhibited by PTPRK The STAT3 transcription factor binds to activated MET through phosphorylated tyrosine residue Y1356 of MET | Quan et al. 2019; Xu et al. 2019 |
| STIL | 1 | Activates Plk1 | Castiel et al. 2011 |
| STK11 | max(2 , SKP2) | Constituively activated unless mutated Maximally activated by high SKP2 activity | Lee et al. 2015; Lin et al. 2015 |
| TCF | avg(pos)-avg(neg) | | |
| toxic NHEJ | NHEJ_Ligation_Complex*min(1 , UrseDSB)-G2_Repair | | |
| TSC | max(GSK3B , AMPK) - floor(avg(ERK , Akt , Akt , Akt , p90RSK)) + 1 | Activated by GSK3B and AMPK inhibited by ERK Akt and p90RSK | Corradetti et al. 2004; Jiang et al. 2019; Tian et al. 2019; Mo et al. 2020; Kwiatkowski and Manning 2005 |
| Twist | avg(pos)-avg(neg) | Activated by Wnt inhibited by ZNRF3 | |
| UrDSB | DSB-floor(avg(G2_Repair , NHEJ_Ligation_Complex)) | Unrepaired DNA double strand breaks are repaird by HR NHEJ and MMEJ | Mao et al. 2008; Seol et al. 2018 |
| UrseDSB | avg(pos)-avg(neg) | | |
| Wee1 | max(3-Plk1 , floor(avg(CHK1 , CHK2))) | Activated by Plk1 CHK1 and CHK2 | Watanabe et al. 2004 |
| WNT | 3-SFRP4 | Inhibited by SFRP4 | Park et al. 2008 |
| XLF | 1 | | |
| XRCC4 | 1 | Component of the NHEJ Ligation Complex | Koch et al. 2004 |
| YB1 | avg(pos)-avg(neg) | | |
| ZNRF3 | avg(pos)-avg(neg) | Inhibits Frizzled | Chang et al. 2020 |

**Supplementary Table S2. Target functions used in the *in silico* model.** The target function for each node is specified, along with a brief description of the mechanism and a reference where required. Notes on the syntax of the target functions are available from https://biomodelanalyzer.org/. A number in the Target Function column represents nodes that are set to a constant value.

## Table References


Adams, J. M., Huang, D. C. S., Strasser, A., Willis, S., Chen, L., Wei, A., van Delft, M., Fletcher, J. I., Puthalakath, H., Kuroda, J., Michalak, E. M., Kelly, P. N., Bouillet, P., Villunger, A., O'Reilly, L., Bath, M. L., Smith, D. P., Egle, A., Harris, A. W., Hinds, M., Colman, P., and Cory, S. (2005). Subversion of the bcl-2 life/death switch in cancer development and therapy. *Cold Spring Harbor symposia on quantitative biology*, 70:469–77.

Ahnesorg, P., Smith, P., and Jackson, S. P. (2006). Xlf interacts with the xrcc4-dna ligase iv complex to promote dna nonhomologous end-joining. *Cell*, 124(2):301–13.

Al Bitar, S. and Gali-Muhtasib, H. (2019). The role of the cyclin dependent kinase inhibitor p21cip1/waf1 in targeting cancer: Molecular mechanisms and novel therapeutics. *Cancers*, 11(10).

Alexander, J. L. and Orr-Weaver, T. L. (2016). Replication fork instability and the consequences of fork collisions from rereplication. *Genes & development*, 30(20):2241–2252.

Allan, L. A., Morrice, N., Brady, S., Magee, G., Pathak, S., and Clarke, P. R. (2003). Inhibition of caspase-9 through phosphorylation at thr 125 by erk mapk. *Nature cell biology*, 5(7):647–54.

Alvarez, B., Martínez-A, C., Burgering, B. M., and Carrera, A. C. (2001). Forkhead transcription factors contribute to execution of the mitotic programme in mammals. *Nature*, 413(6857):744–747.

Appanah, R., Jones, D., Falquet, B., and Rass, U. (2020). Limiting homologous recombination at stalled replication forks is essential for cell viability: Dna2 to the rescue. *Current genetics*, 66(6):1085–1092.

Assoian, R. K. and Klein, E. A. (2008). Growth control by intracellular tension and extracellular stiffness. *Trends in cell biology*, 18(7):347–52.

Barnard, S., Bouffler, S., and Rothkamm, K. (2013). The shape of the radiation dose response for dna double-strand break induction and repair. *Genome integrity*, 4(1):1.

Barnhart, B. C., Lam, J. C., Young, R. M., Houghton, P. J., Keith, B., and Simon, M. C. (2008). Effects of 4e-bp1 expression on hypoxic cell cycle inhibition and tumor cell proliferation and survival. *Cancer biology & therapy*, 7(9):1441–9.

Barrera-Rodríguez, R. (2018). Importance of the keap1-nrf2 pathway in nsclc: Is it a possible biomarker? *Biomedical reports*, 9(5):375–382.

Bartocci, C. and Denchi, E. L. (2013). Put a ring on it: regulation and inhibition of rnf8 and rnf168 ring finger e3 ligases at dna damage sites. *Frontiers in genetics*, 4:128.

Baudino, T. A. and Cleveland, J. L. (2001). The max network gone mad. *Molecular and cellular biology*, 21(3):691–702.

Bergoug, M., Doudeau, M., Godin, F., Mosrin, C., Vallée, B., and Bénédetti, H. (2020). Neurofibromin structure, functions and regulation. *Cells*, 9(11).

Bertoli, C., Skotheim, J. M., and de Bruin, R. A. M. (2013). Control of cell cycle transcription during g1 and s phases. *Nature reviews. Molecular cell biology*, 14(8):518–28.

Bidkhori, G., Moeini, A., and Masoudi-Nejad, A. (2012). Modeling of tumor progression in nsclc and intrinsic resistance to tki in loss of pten expression. *PloS one*, 7(10):e48004.

Bigenzahn, J. W., Collu, G. M., Kartnig, F., Pieraks, M., Vladimer, G. I., Heinz, L. X., Sedlyarov, V., Schischlik, F., Fauster, A., Rebsamen, M., Parapatics, K., Blomen, V. A., Müller, A. C., Winter, G. E., Kralovics, R., Brummelkamp, T. R., Mlodzik, M., and Superti-Furga, G. (2018). Lztr1 is a regulator of ras ubiquitination and signaling. *Science (New York, N.Y.)*, 362(6419):1171–1177.

Bockstaele, L., Coulonval, K., Kooken, H., Paternot, S., and Roger, P. P. (2006). Regulation of cdk4. *Cell division*, 1:25.

Boichuk, S., Hu, L., Makielski, K., Pandolfi, P. P., and Gjoerup, O. V. (2011). Functional connection between rad51 and pml in homology-directed repair. *PloS one*, 6(10):e25814.

Boriack-Sjodin, P. A., Margarit, S. M., Bar-Sagi, D., and Kuriyan, J. (1998). The structural basis of the activation of ras by sos. *Nature*, 394(6691):337–43.

Bousoik, E. and Montazeri Aliabadi, H. (2018). ”do we know jack” about jak? a closer look at jak/stat signaling pathway. *Frontiers in oncology*, 8:287.

Brastianos, P. K., Dooley, K., Geyer, S., Gerstner, E. R., Kaufmann, T. J., Iafrate, A. J., Martinez-Lage, M., Milhem, M., Welch, M. R., Kaley, T. J., Drappatz, J., Chan, A., Kumthekar, P., Matsuoka, C. K., Strowd, R. E., Cohen, A. L., Jaeckle, K., Robell, L., Magge, R. S., Nam, J. Y., Blondin, N., Shaikh, N., Rabinowitz, I., Thomas, A. A., Piccioni, D. E., Brown, P., Kaluziak, S., Codd, E., Cahill, D. P., Santagata, S., Barker, F. G., and Galanis, E. (2026). Abemaciclib in meningiomas with somatic nf2 or cdk pathway alterations: the phase 2 alliance a071401 trial. *Nature medicine*, 32(2):717–724.

Buscemi, G., Perego, P., Carenini, N., Nakanishi, M., Chessa, L., Chen, J., Khanna, K., and Delia, D. (2004). Activation of atm and chk2 kinases in relation to the amount of dna strand breaks. *Oncogene*, 23(46):7691–700.

Cai, Z., Simons, D. L., Fu, X.-Y., Feng, G.-S., Wu, S. M., and Zhang, X. (2011). Loss of shp2-mediated mitogen-activated protein kinase signaling in muller glial cells results in retinal degeneration. *Molecular and cellular biology*, 31(14):2973–83.

Cardone, M. H., Roy, N., Stennicke, H. R., Salvesen, G. S., Franke, T. F., Stanbridge, E., Frisch, S., and Reed, J. C. (1998). Regulation of cell death protease caspase-9 by phosphorylation. *Science (New York, N.Y.)*, 282(5392):1318–21.

Castedo, M., Perfettini, J.-L., Roumier, T., and Kroemer, G. (2002a). Cyclin-dependent kinase-1: linking apoptosis to cell cycle and mitotic catastrophe. *Cell death and differentiation*, 9(12):1287–93.

Castedo, M., Roumier, T., Blanco, J., Ferri, K. F., Barretina, J., Tintignac, L. A., Andreau, K., Perfettini, J.-L., Amendola, A., Nardacci, R., Leduc, P., Ingber, D. E., Druillennec, S., Roques, B., Leibovitch, S. A., Vilella-Bach, M., Chen, J., Este, J. A., Modjtahedi, N., Piacentini, M., and Kroemer, G. (2002b). Sequential involvement of cdk1, mtor and p53 in apoptosis induced by the hiv-1 envelope. *The EMBO journal*, 21(15):4070–80.

Castiel, A., Danieli, M. M., David, A., Moshkovitz, S., Aplan, P. D., Kirsch, I. R., Brandeis, M., Krämer, A., and Izraeli, S. (2011). The stil protein regulates centrosome integrity and mitosis through suppression of chfr. *Journal of cell science*, 124(Pt 4):532–9.

Chang, C., Zhao, W., Xie, B., Deng, Y., Han, T., Cui, Y., Dai, Y., Zhang, Z., Gao, J., Guo, H., and Yan, J. (2014). Pao pereira extract suppresses castration-resistant prostate cancer cell growth, survival, and invasion through inhibition of nf$\kappa$b signaling. *Integrative cancer therapies*, 13(3):249–58.

Chang, L.-S., Kim, M., Glinka, A., Reinhard, C., and Niehrs, C. (2020). The tumor suppressor ptprk promotes znrf3 internalization and is required for wnt inhibition in the spemann organizer. *eLife*, 9.

Chen, B.-R., Wang, Y., Shen, Z.-J., Bennett, A., Hindi, I., Tyler, J. K., and Sleckman, B. P. (2021). The rnf8 and rnf168 ubiquitin ligases regulate pro- and anti-resection activities at broken dna ends during non-homologous end joining. *DNA repair*, 108:103217.

Chen, H., Chen, W., Zhang, X., Hu, L., Tang, G., Kong, J., and Wang, Z. (2019). E26 transformation (ets)-specific related transcription factor-3 (elf3) orchestrates a positive feedback loop that constitutively activates the mapk/erk pathway to drive thyroid cancer. *Oncology reports*, 41(1):570–578.

Chen, L., Willis, S. N., Wei, A., Smith, B. J., Fletcher, J. I., Hinds, M. G., Colman, P. M., Day, C. L., Adams, J. M., and Huang, D. C. S. (2005). Differential targeting of prosurvival bcl-2 proteins by their bh3-only ligands allows complementary apoptotic function. *Molecular cell*, 17(3):393–403.

Cheng, A. N., Fan, C.-C., Lo, Y.-K., Kuo, C.-L., Wang, H.-C., Lien, I.-H., Lin, S.-Y., Chen, C.-H., Jiang, S. S., Chang, I.-S., Juan, H.-F., Lyu, P.-C., and Lee, A. Y.-L. (2017). Cdc7-dbf4-mediated phosphorylation of hsp90-s164 stabilizes hsp90-hclk2-mrn complex to enhance atr/atm signaling that overcomes replication stress in cancer. *Scientific reports*, 7(1):17024.

Chiang, Y.-C., Teng, S.-C., Su, Y.-N., Hsieh, F.-J., and Wu, K.-J. (2003). c-myc directly regulates the transcription of the nbs1 gene involved in dna double-strand break repair. *The Journal of biological chemistry*, 278(21):19286–91.

Chène, P. (2003). Inhibiting the p53-mdm2 interaction: an important target for cancer therapy. *Nature reviews. Cancer*, 3(2):102–9.

Cloud, V., Chan, Y.-L., Grubb, J., Budke, B., and Bishop, D. K. (2012). Rad51 is an accessory factor for dmc1-mediated joint molecule formation during meiosis. *Science (New York, N.Y.)*, 337(6099):1222–5.

Cong, F., Schweizer, L., and Varmus, H. (2004). Wnt signals across the plasma membrane to activate the beta-catenin pathway by forming oligomers containing its receptors, frizzled and lrp. *Development (Cambridge, England)*, 131(20):5103–15.

Corradetti, M. N., Inoki, K., Bardeesy, N., DePinho, R. A., and Guan, K.-L. (2004). Regulation of the tsc pathway by lkb1: evidence of a molecular link between tuberous sclerosis complex and peutz-jeghers syndrome. *Genes & development*, 18(13):1533–8.

Cortez, D. (2015). Preventing replication fork collapse to maintain genome integrity. *DNA repair*, 32:149–157.

Crasta, K., Ganem, N. J., Dagher, R., Lantermann, A. B., Ivanova, E. V., Pan, Y., Nezi, L., Protopopov, A., Chowdhury, D., and Pellman, D. (2012). Dna breaks and chromosome pulverization from errors in mitosis. *Nature*, 482(7383):53–8.

Cruz-García, A., López-Saavedra, A., and Huertas, P. (2014). Brca1 accelerates ctip-mediated dna-end resection. *Cell reports*, 9(2):451–9.

Daniel, M., Peek, G. W., and Tollefsbol, T. O. (2012). Regulation of the human catalytic subunit of telomerase (htert). *Gene*, 498(2):135–46.

Datar, S. A., Galloni, M., de la Cruz, A., Marti, M., Edgar, B. A., and Frei, C. (2006). Mammalian cyclin d1/cdk4 complexes induce cell growth in drosophila. *Cell cycle (Georgetown, Tex.)*, 5(6):647–52.

Datta, S. R., Dudek, H., Tao, X., Masters, S., Fu, H., Gotoh, Y., and Greenberg, M. E. (1997). Akt phosphorylation of bad couples survival signals to the cell-intrinsic death machinery. *Cell*, 91(2):231–41.

Davis, A. J., Chen, B. P. C., and Chen, D. J. (2014). Dna-pk: a dynamic enzyme in a versatile dsb repair pathway. *DNA repair*, 17:21–9.

Diolaiti, D., McFerrin, L., Carroll, P. A., and Eisenman, R. N. (2015). Functional interactions among members of the max and mlx transcriptional network during oncogenesis. *Biochimica et biophysica acta*, 1849(5):484–500.

Dobbs, T. A., Tainer, J. A., and Lees-Miller, S. P. (2010). A structural model for regulation of nhej by dna-pkcs autophosphorylation. *DNA repair*, 9(12):1307–14.

Dupré, A., Boyer-Chatenet, L., and Gautier, J. (2006). Two-step activation of atm by dna and the mre11-rad50-nbs1 complex. *Nature structural & molecular biology*, 13(5):451–7.

Eeckhoute, J., Carroll, J. S., Geistlinger, T. R., Torres-Arzayus, M. I., and Brown, M. (2006). A cell-type-specific transcriptional network required for estrogen regulation of cyclin d1 and cell cycle progression in breast cancer. *Genes & development*, 20(18):2513–26.

Eliezer, Y., Argaman, L., Kornowski, M., Roniger, M., and Goldberg, M. (2014). Interplay between the dna damage proteins mdc1 and atm in the regulation of the spindle assembly checkpoint. *The Journal of biological chemistry*, 289(12):8182–93.

Engelman, J. A., Zejnullahu, K., Mitsudomi, T., Song, Y., Hyland, C., Park, J. O., Lindeman, N., Gale, C.-M., Zhao, X., Christensen, J., Kosaka, T., Holmes, A. J., Rogers, A. M., Cappuzzo, F., Mok, T., Lee, C., Johnson, B. E., Cantley, L. C., and Jänne, P. A. (2007). Met amplification leads to gefitinib resistance in lung cancer by activating erbb3 signaling. *Science (New York, N.Y.)*, 316(5827):1039–43.

Esashi, F., Galkin, V. E., Yu, X., Egelman, E. H., and West, S. C. (2007). Stabilization of rad51 nucleoprotein filaments by the c-terminal region of brca2. *Nature structural & molecular biology*, 14(6):468–74.

Gao, J., McConnell, M. J., Yu, B., Li, J., Balko, J. M., Black, E. P., Johnson, J. O., Lloyd, M. C., Altiok, S., and Haura, E. B. (2009). Muc1 is a downstream target of stat3 and regulates lung cancer cell survival and invasion. *International journal of oncology*, 35(2):337–45.

García-Gutiérrez, L., Delgado, M. D., and León, J. (2019). Myc oncogene contributions to release of cell cycle brakes. *Genes*, 10(3).

Gheghiani, L., Loew, D., Lombard, B., Mansfeld, J., and Gavet, O. (2017). Plk1 activation in late g2 sets up commitment to mitosis. *Cell reports*, 19(10):2060–2073.

Giacinti, C. and Giordano, A. (2006). Rb and cell cycle progression. *Oncogene*, 25(38):5220–7.

Golstein, P. and Kroemer, G. (2007). A multiplicity of cell death pathways. symposium on apoptotic and non-apoptotic cell death pathways. *EMBO reports*, 8(9):829–33.

Gong, J., Zhang, J.-P., Li, B., Zeng, C., You, K., Chen, M.-X., Yuan, Y., and Zhuang, S.-M. (2013). Microrna-125b promotes apoptosis by regulating the expression of mcl-1, bcl-w and il-6r. *Oncogene*, 32(25):3071–9.

Gu, J., Lu, H., Tsai, A. G., Schwarz, K., and Lieber, M. R. (2007). Single-stranded dna ligation and xlf-stimulated incompatible dna end ligation by the xrcc4-dna ligase iv complex: influence of terminal dna sequence. *Nucleic acids research*, 35(17):5755–62.

Gudas, J. M., Payton, M., Thukral, S., Chen, E., Bass, M., Robinson, M. O., and Coats, S. (1999). Cyclin e2, a novel g1 cyclin that binds cdk2 and is aberrantly expressed in human cancers. *Molecular and cellular biology*, 19(1):612–22.

Guo, J., Li, X., Zhang, W., Chen, Y., Zhu, S., Chen, L., Xu, R., Lv, Y., Wu, D., Guo, M., Liu, X., Lu, W., and Deng, H. (2019). Hsp60-regulated mitochondrial proteostasis and protein translation promote tumor growth of ovarian cancer. *Scientific reports*, 9(1):12628.

Guo, Y., Du, J., and Kwiatkowski, D. J. (2013). Molecular dissection of akt activation in lung cancer cell lines. *Molecular cancer research : MCR*, 11(3):282–93.

Han, J., Wan, L., Jiang, G., Cao, L., Xia, F., Tian, T., Zhu, X., Wu, M., Huen, M. S. Y., Wang, Y., Liu, T., and Huang, J. (2021). Atm controls the extent of dna end resection by eliciting sequential posttranslational modifications of ctip. *Proceedings of the National Academy of Sciences of the United States of America*, 118(12).

Harada, M., Kotake, Y., Ohhata, T., Kitagawa, K., Niida, H., Matsuura, S., Funai, K., Sugimura, H., Suda, T., and Kitagawa, M. (2014). Yb-1 promotes transcription of cyclin d1 in human non-small-cell lung cancers. *Genes to cells : devoted to molecular & cellular mechanisms*, 19(6):504–16.

He, G., Siddik, Z. H., Huang, Z., Wang, R., Koomen, J., Kobayashi, R., Khokhar, A. R., and Kuang, J. (2005). Induction of p21 by p53 following dna damage inhibits both cdk4 and cdk2 activities. *Oncogene*, 24(18):2929–43.

Hervieu, A. and Kermorgant, S. (2018). The role of pi3k in met driven cancer: A recap. *Frontiers in molecular biosciences*, 5:86.

Hinz, M., Krappmann, D., Eichten, A., Heder, A., Scheidereit, C., and Strauss, M. (1999). Nf-kappab function in growth control: regulation of cyclin d1 expression and g0/g1-to-s-phase transition. *Molecular and cellular biology*, 19(4):2690–8.

Hu, Y., Wang, C., Huang, K., Xia, F., Parvin, J. D., and Mondal, N. (2014). Regulation of 53bp1 protein stability by rnf8 and rnf168 is important for efficient dna double-strand break repair. *PloS one*, 9(10):e110522.

Huang, H. and He, X. (2008). Wnt/beta-catenin signaling: new (and old) players and new insights. *Current opinion in cell biology*, 20(2):119–25.

Huen, M. S. Y., Grant, R., Manke, I., Minn, K., Yu, X., Yaffe, M. B., and Chen, J. (2007). Rnf8 transduces the dna-damage signal via histone ubiquitylation and checkpoint protein assembly. *Cell*, 131(5):901–14.

Hurbin, A., Coll, J.-L., Dubrez-Daloz, L., Mari, B., Auberger, P., Brambilla, C., and Favrot, M.-C. (2005). Cooperation of amphiregulin and insulin-like growth factor-1 inhibits bax- and bad-mediated apoptosis via a protein kinase c-dependent pathway in non-small cell lung cancer cells. *The Journal of biological chemistry*, 280(20):19757–67.

Ismail, I. H., Nyström, S., Nygren, J., and Hammarsten, O. (2005). Activation of ataxia telangiectasia mutated by dna strand break-inducing agents correlates closely with the number of dna double strand breaks. *The Journal of biological chemistry*, 280(6):4649–55.

Jiang, C., Bi, C., Jiang, X., Tian, T., Huang, X., Wang, C., Fernandez, M. R., Iqbal, J., Chan, W. C., McKeithan, T. W., Lewis, R. E., and Fu, K. (2019). The mir-17 92 cluster activates mtorc1 in mantle cell lymphoma by targeting multiple regulators in the stk11/ampk/tsc/mtor pathway. *British journal of haematology*, 185(3):616–620.

Jin, L., Hu, W. L., Jiang, C. C., Wang, J. X., Han, C. C., Chu, P., Zhang, L. J., Thorne, R. F., Wilmott, J., Scolyer, R. A., Hersey, P., Zhang, X. D., and Wu, M. (2011). Microrna-149*, a p53-responsive microrna, functions as an oncogenic regulator in human melanoma. *Proceedings of the National Academy of Sciences of the United States of America*, 108(38):15840–5.

Kalkavan, H. and Green, D. R. (2018). Momp, cell suicide as a bcl-2 family business. *Cell death and differentiation*, 25(1):46–55.

Kang, H., Heo, S., Shin, J. J., Ji, E., Tak, H., Ahn, S., Lee, K. J., Lee, E. K., and Kim, W. (2019). A mir-194/ptbp1/ccnd3 axis regulates tumor growth in human hepatocellular carcinoma. *The Journal of pathology*, 249(3):395–408.

Kang, X.-H., Zhang, J.-H., Zhang, Q.-Q., Cui, Y.-H., Wang, Y., Kou, W.-Z., Miao, Z.-H., Lu, P., Wang, L.-F., Xu, Z.-Y., and Cao, F. (2017). Degradation of mcl-1 through gsk-3$\beta$ activation regulates apoptosis induced by bufalin in non-small cell lung cancer h1975 cells. *Cellular physiology and biochemistry : international journal of experimental cellular physiology, biochemistry, and pharmacology*, 41(5):2067–2076.

Karlsson-Rosenthal, C. and Millar, J. B. A. (2006). Cdc25: Mechanisms of checkpoint inhibition and recovery. *Trends in Cell Biology*, 16(6):285–292.

Kato, G. J., Lee, W. M., Chen, L. L., and Dang, C. V. (1992). Max: functional domains and interaction with c-myc. *Genes & development*, 6(1):81–92.

Kharbanda, A., Rajabi, H., Jin, C., Tchaicha, J., Kikuchi, E., Wong, K.-K., and Kufe, D. (2014). Targeting the oncogenic muc1-c protein inhibits mutant egfr-mediated signaling and survival in non-small cell lung cancer cells. *Clinical cancer research : an official journal of the American Association for Cancer Research*, 20(21):5423–34.

Khattar, E. and Tergaonkar, V. (2017). Transcriptional regulation of telomerase reverse transcriptase (tert) by myc. *Frontiers in cell and developmental biology*, 5:1.

Kobayashi, J., Antoccia, A., Tauchi, H., Matsuura, S., and Komatsu, K. (2004). Nbs1 and its functional role in the dna damage response. *DNA repair*, 3(8-9):855–61.

Koch, C. A., Agyei, R., Galicia, S., Metalnikov, P., O'Donnell, P., Starostine, A., Weinfeld, M., and Durocher, D. (2004). Xrcc4 physically links dna end processing by polynucleotide kinase to dna ligation by dna ligase iv. *The EMBO journal*, 23(19):3874–85.

Komori, H., Enomoto, M., Nakamura, M., Iwanaga, R., and Ohtani, K. (2005). Distinct e2f-mediated transcriptional program regulates p14arf gene expression. *The EMBO journal*, 24(21):3724–36.

Kreis, N.-N., Louwen, F., and Yuan, J. (2015). Less understood issues: p21(cip1) in mitosis and its therapeutic potential. *Oncogene*, 34(14):1758–67.

Kwiatkowski, D. J. and Manning, B. D. (2005). Tuberous sclerosis: a gap at the crossroads of multiple signaling pathways. *Human molecular genetics*, 14 Spec No. 2:R251–8.

Langerak, P., Mejia-Ramirez, E., Limbo, O., and Russell, P. (2011). Release of ku and mrn from dna ends by mre11 nuclease activity and ctp1 is required for homologous recombination repair of double-strand breaks. *PLoS genetics*, 7(9):e1002271.

Leber, B., Kale, J., and Andrews, D. W. (2018). Unleashing blocked apoptosis in cancer cells: New mcl1 inhibitors find their groove. *Cancer discovery*, 8(12):1511–1514.

Lee, J.-H., Goodarzi, A. A., Jeggo, P. A., and Paull, T. T. (2010a). 53bp1 promotes atm activity through direct interactions with the mrn complex. *The EMBO journal*, 29(3):574–85.

Lee, J.-H., Kang, Y., Khare, V., Jin, Z.-Y., Kang, M.-Y., Yoon, Y., Hyun, J.-W., Chung, M.-H., Cho, S.-I., Jun, J. Y., Chang, I.-Y., and You, H. J. (2010b). The p53-inducible gene 3 (pig3) contributes to early cellular response to dna damage. *Oncogene*, 29(10):1431–50.

Lee, J. Y., Engelman, J. A., and Cantley, L. C. (2007). Biochemistry. pi3k charges ahead. *Science (New York, N.Y.)*, 317(5835):206–7.

Lee, K. B., Kim, K.-R., Huh, T.-L., and Lee, Y. M. (2008). Proton induces apoptosis of hypoxic tumor cells by the p53-dependent and p38/jnk mapk signaling pathways. *International journal of oncology*, 33(6):1247–56.

Lee, S.-W., Li, C.-F., Jin, G., Cai, Z., Han, F., Chan, C.-H., Yang, W.-L., Li, B.-K., Rezaeian, A. H., Li, H.-Y., Huang, H.-Y., and Lin, H.-K. (2015). Skp2-dependent ubiquitination and activation of lkb1 is essential for cancer cell survival under energy stress. *Molecular cell*, 57(6):1022–1033.

Li, H., Sun, L., Tang, Z., Fu, L., Xu, Y., Li, Z., Luo, W., Qiu, X., and Wang, E. (2012). Overexpression of trim24 correlates with tumor progression in non-small cell lung cancer. *PloS one*, 7(5):e37657.

Li, H., Zou, T., Meng, S., Peng, Y.-Z., and Yang, J.-F. (2018). p21 protects cardiomyocytes against ischemia-reperfusion injury by inhibiting oxidative stress. *Molecular medicine reports*, 17(3):4665–4671.

Li, X., Yang, X., Liu, Y., Gong, N., Yao, W., Chen, P., Qin, J., Jin, H., Li, J., Chu, R., Shan, L., Zhang, R., Zhang, W., and Wang, H. (2013). Japonicone a suppresses growth of burkitt lymphoma cells through its effect on nf-$\kappa$b. *Clinical cancer research : an official journal of the American Association for Cancer Research*, 19(11):2917–28.

Liang, H. and Fesik, S. W. (1997). Three-dimensional structures of proteins involved in programmed cell death. *Journal of molecular biology*, 274(3):291–302.

Liao, G.-B., Li, X.-Z., Zeng, S., Liu, C., Yang, S.-M., Yang, L., Hu, C.-J., and Bai, J.-Y. (2018). Regulation of the master regulator foxm1 in cancer. *Cell communication and signaling : CCS*, 16(1):57.

Lin, R., Elf, S., Shan, C., Kang, H.-B., Ji, Q., Zhou, L., Hitosugi, T., Zhang, L., Zhang, S., Seo, J. H., Xie, J., Tucker, M., Gu, T.-L., Sudderth, J., Jiang, L., Mitsche, M., DeBerardinis, R. J., Wu, S., Li, Y., Mao, H., Chen, P. R., Wang, D., Chen, G. Z., Hurwitz, S. J., Lonial, S., Arellano, M. L., Khoury, H. J., Khuri, F. R., Lee, B. H., Lei, Q., Brat, D. J., Ye, K., Boggon, T. J., He, C., Kang, S., Fan, J., and Chen, J. (2015). 6-phosphogluconate dehydrogenase links oxidative ppp, lipogenesis and tumour growth by inhibiting lkb1-ampk signalling. *Nature cell biology*, 17(11):1484–96.

Liu, Z., Zhang, J., Wu, L., Liu, J., and Zhang, M. (2014). Overexpression of gnao1 correlates with poor prognosis in patients with gastric cancer and plays a role in gastric cancer cell proliferation and apoptosis. *International journal of molecular medicine*, 33(3):589–96.

Lu, W., Yu, T., Liu, S., Li, S., Li, S., Liu, J., Xu, Y., Xing, H., Tian, Z., Tang, K., Rao, Q., Wang, J., and Wang, M. (2017). Fhl2 interacts with iaspp and impacts the biological functions of leukemia cells. *Oncotarget*, 8(25):40885–40895.

Mahaney, B. L., Meek, K., and Lees-Miller, S. P. (2009). Repair of ionizing radiation-induced dna double-strand breaks by non-homologous end-joining. *The Biochemical journal*, 417(3):639–50.

Mailand, N., Podtelejnikov, A. V., Groth, A., Mann, M., Bartek, J., and Lukas, J. (2002). Regulation of G(2)/M events by Cdc25A through phosphorylation-dependent modulation of its stability. *The EMBO journal*, 21(21):5911–5920.

Mao, Z., Bozzella, M., Seluanov, A., and Gorbunova, V. (2008). Dna repair by nonhomologous end joining and homologous recombination during cell cycle in human cells. *Cell cycle (Georgetown, Tex.)*, 7(18):2902–6.

Mari, P.-O., Florea, B. I., Persengiev, S. P., Verkaik, N. S., Brüggenwirth, H. T., Modesti, M., Giglia-Mari, G., Bezstarosti, K., Demmers, J. A. A., Luider, T. M., Houtsmuller, A. B., and van Gent, D. C. (2006). Dynamic assembly of end-joining complexes requires interaction between ku70/80 and xrcc4. *Proceedings of the National Academy of Sciences of the United States of America*, 103(49):18597–602.

Martinez-Martí, A. and Felip, E. (2011). Pi3k pathway in nsclc. *Frontiers in oncology*, 1:55.

Maréchal, A. and Zou, L. (2013). Dna damage sensing by the atm and atr kinases. *Cold Spring Harbor perspectives in biology*, 5(9).

Meng, J., Dai, B., Fang, B., Bekele, B. N., Bornmann, W. G., Sun, D., Peng, Z., Herbst, R. S., Papadimitrakopoulou, V., Minna, J. D., Peyton, M., and Roth, J. A. (2010a). Combination treatment with mek and akt inhibitors is more effective than each drug alone in human non-small cell lung cancer in vitro and in vivo. *PloS one*, 5(11):e14124.

Meng, J., Fang, B., Liao, Y., Chresta, C. M., Smith, P. D., and Roth, J. A. (2010b). Apoptosis induction by mek inhibition in human lung cancer cells is mediated by bim. *PloS one*, 5(9):e13026.

Mikac, S., Rychłowski, M., Dziadosz, A., Szabelska-Beresewicz, A., Fahraeus, R., Hupp, T., and Sznarkowska, A. (2021). Identification of a stable, non-canonically regulated nrf2 form in lung cancer cells. *Antioxidants (Basel, Switzerland)*, 10(5).

Mo, Y., Wu, Y., Li, X., Rao, H., Tian, X., Wu, D., Qiu, Z., Zheng, G., and Hu, J. (2020). Osthole delays hepatocarcinogenesis in mice by suppressing akt/fasn axis and erk phosphorylation. *European journal of pharmacology*, 867:172788.

Nakada, S., Yonamine, R. M., and Matsuo, K. (2012). Rnf8 regulates assembly of rad51 at dna double-strand breaks in the absence of brca1 and 53bp1. *Cancer research*, 72(19):4974–83.

Nakano, K. and Vousden, K. H. (2001). Puma, a novel proapoptotic gene, is induced by p53. *Molecular cell*, 7(3):683–94.

Nakayama, S., Sng, N., Carretero, J., Welner, R., Hayashi, Y., Yamamoto, M., Tan, A. J., Yamaguchi, N., Yasuda, H., Li, D., Soejima, K., Soo, R. A., Costa, D. B., Wong, K.-K., and Kobayashi, S. S. (2014). $\beta$-catenin contributes to lung tumor development induced by egfr mutations. *Cancer research*, 74(20):5891–902.

Nestal de Moraes, G., Bella, L., Zona, S., J Burton, M., and W-F Lam, E. (2016). Insights into a critical role of the foxo3a-foxm1 axis in dna damage response and genotoxic drug resistance. *Current drug targets*, 17(2):164–177.

Nguyen, T., Nioi, P., and Pickett, C. B. (2009). The nrf2-antioxidant response element signaling pathway and its activation by oxidative stress. *The Journal of biological chemistry*, 284(20):13291–5.

Nguyen, T. T. T., Tran, E., Nguyen, T. H., Do, P. T., Huynh, T. H., and Huynh, H. (2004). The role of activated mek-erk pathway in quercetin-induced growth inhibition and apoptosis in a549 lung cancer cells. *Carcinogenesis*, 25(5):647–59.

Niture, S. K. and Jaiswal, A. K. (2013). Nrf2-induced antiapoptotic bcl-xl protein enhances cell survival and drug resistance. *Free radical biology & medicine*, 57:119–31.

Noguchi, K., Kitanaka, C., Yamana, H., Kokubu, A., Mochizuki, T., and Kuchino, Y. (1999). Regulation of c-myc through phosphorylation at ser-62 and ser-71 by c-jun n-terminal kinase. *The Journal of biological chemistry*, 274(46):32580–7.

Oh, H.-N., Lee, M.-H., Kim, E., Kwak, A.-W., Seo, J.-H., Yoon, G., Cho, S.-S., Choi, J.-S., Lee, S.-M., Seo, K.-S., Chae, J.-I., and Shim, J.-H. (2020). Dual inhibition of egfr and met by echinatin retards cell growth and induces apoptosis of lung cancer cells sensitive or resistant to gefitinib. *Phytotherapy research : PTR*, 34(2):388–400.

Organ, S. L. and Tsao, M.-S. (2011). An overview of the c-met signaling pathway. *Therapeutic advances in medical oncology*, 3(1 Suppl):S7–S19.

Palakurthy, R. K., Wajapeyee, N., Santra, M. K., Gazin, C., Lin, L., Gobeil, S., and Green, M. R. (2009). Epigenetic silencing of the rassf1a tumor suppressor gene through hoxb3-mediated induction of dnmt3b expression. *Molecular cell*, 36(2):219–30.

Park, J.-R., Jung, J.-W., Lee, Y.-S., and Kang, K.-S. (2008). The roles of wnt antagonists dkk1 and sfrp4 during adipogenesis of human adipose tissue-derived mesenchymal stem cells. *Cell proliferation*, 41(6):859–874.

Park, K. H., Seol, J. Y., Yoo, C. G., Kim, Y. W., Han, S. K., Lee, E. H., Kim, C. M., Shim, Y. S., and Lee, C. T. (2001). Adenovirus expressing p27(kip1) induces growth arrest of lung cancer cell lines and suppresses the growth of established lung cancer xenografts. *Lung cancer (Amsterdam, Netherlands)*, 31(2-3):149–55.

Paulsen, R. D. and Cimprich, K. A. (2007). The atr pathway: fine-tuning the fork. *DNA repair*, 6(7):953–66.

Qi, W. and Martinez, J. D. (2003). Reduction of 14-3-3 proteins correlates with increased sensitivity to killing of human lung cancer cells by ionizing radiation. *Radiation research*, 160(2):217–23.

Quan, Z., Zhang, B.-B., Yin, F., Du, J., Zhi, Y.-T., Xu, J., and Song, N. (2019). Ddx5 silencing suppresses the migration of basal cell carcinoma cells by downregulating jak2/stat3 pathway. *Technology in cancer research & treatment*, 18:1533033819892258.

Raghav, K. P. S., Gonzalez-Angulo, A. M., and Blumenschein, G. R. (2012). Role of hgf/met axis in resistance of lung cancer to contemporary management. *Translational lung cancer research*, 1(3):179–93.

Raina, D., Kosugi, M., Ahmad, R., Panchamoorthy, G., Rajabi, H., Alam, M., Shimamura, T., Shapiro, G. I., Supko, J., Kharbanda, S., and Kufe, D. (2011). Dependence on the muc1-c oncoprotein in non-small cell lung cancer cells. *Molecular cancer therapeutics*, 10(5):806–16.

Ray, K., Ujvari, B., Ramana, V., and Donald, J. (2018). Cross-talk between egfr and il-6 drives oncogenic signaling and offers therapeutic opportunities in cancer. *Cytokine & growth factor reviews*, 41:18–27.

Renault, V. M., Thekkat, P. U., Hoang, K. L., White, J. L., Brady, C. A., Kenzelmann Broz, D., Venturelli, O. S., Johnson, T. M., Oskoui, P. R., Xuan, Z., Santo, E. E., Zhang, M. Q., Vogel, H., Attardi, L. D., and Brunet, A. (2011). The pro-longevity gene foxo3 is a direct target of the p53 tumor suppressor. *Oncogene*, 30(29):3207–21.

Roberts, P. J. and Der, C. J. (2007). Targeting the raf-mek-erk mitogen-activated protein kinase cascade for the treatment of cancer. *Oncogene*, 26(22):3291–310.

Roos, W. P. and Kaina, B. (2006). Dna damage-induced cell death by apoptosis. *Trends in molecular medicine*, 12(9):440–50.

Sacco, J. J. and Clague, M. J. (2015). Dysregulation of the met pathway in non-small cell lung cancer: implications for drug targeting and resistance. *Translational lung cancer research*, 4(3):242–52.

Seol, J.-H., Shim, E. Y., and Lee, S. E. (2018). Microhomology-mediated end joining: Good, bad and ugly. *Mutation research*, 809:81–87.

Shaw, R. J. and Cantley, L. C. (2006). Ras, pi(3)k and mtor signalling controls tumour cell growth. *Nature*, 441(7092):424–30.

Sheaff, R. J., Groudine, M., Gordon, M., Roberts, J. M., and Clurman, B. E. (1997). Cyclin e-cdk2 is a regulator of p27kip1. *Genes & development*, 11(11):1464–78.

Sherr, C. J. and Roberts, J. M. (1999). Cdk inhibitors: positive and negative regulators of g1-phase progression. *Genes & development*, 13(12):1501–12.

Singh, P. K., Behrens, M. E., Eggers, J. P., Cerny, R. L., Bailey, J. M., Shanmugam, K., Gendler, S. J., Bennett, E. P., and Hollingsworth, M. A. (2008). Phosphorylation of muc1 by met modulates interaction with p53 and mmp1 expression. *The Journal of biological chemistry*, 283(40):26985–95.

Sithanandam, G. and Anderson, L. M. (2008). The erbb3 receptor in cancer and cancer gene therapy. *Cancer gene therapy*, 15(7):413–48.

Song, L., Coppola, D., Livingston, S., Cress, D., and Haura, E. B. (2005). Mcl-1 regulates survival and sensitivity to diverse apoptotic stimuli in human non-small cell lung cancer cells. *Cancer biology & therapy*, 4(3):267–76.

Song, M. S., Song, S. J., Kim, S. Y., Oh, H. J., and Lim, D.-S. (2008). The tumour suppressor rassf1a promotes mdm2 self-ubiquitination by disrupting the mdm2-daxx-hausp complex. *The EMBO journal*, 27(13):1863–74.

Stambolic, V., MacPherson, D., Sas, D., Lin, Y., Snow, B., Jang, Y., Benchimol, S., and Mak, T. W. (2001). Regulation of pten transcription by p53. *Molecular cell*, 8(2):317–25.

Strauss, C. and Goldberg, M. (2011). Recruitment of proteins to dna double-strand breaks: Mdc1 directly recruits rap80. *Cell cycle (Georgetown, Tex.)*, 10(17):2850–7.

Tait, S. W. G. and Green, D. R. (2010). Mitochondria and cell death: outer membrane permeabilization and beyond. *Nature reviews. Molecular cell biology*, 11(9):621–32.

Tait, S. W. G., Parsons, M. J., Llambi, F., Bouchier-Hayes, L., Connell, S., Muñoz-Pinedo, C., and Green, D. R. (2010). Resistance to caspase-independent cell death requires persistence of intact mitochondria. *Developmental cell*, 18(5):802–13.

Takezawa, K., Okamoto, I., Nishio, K., Jänne, P. A., and Nakagawa, K. (2011). Role of erk-bim and stat3-survivin signaling pathways in alk inhibitor-induced apoptosis in eml4-alk-positive lung cancer. *Clinical cancer research : an official journal of the American Association for Cancer Research*, 17(8):2140–8.

Tan, Y., Chen, Y., Yu, L., Zhu, H., Meng, X., Huang, X., Meng, L., Ding, M., Wang, Z., and Shan, L. (2010). Two-fold elevation of expression of foxm1 transcription factor in mouse embryonic fibroblasts enhances cell cycle checkpoint activity by stimulating p21 and chk1 transcription. *Cell proliferation*, 43(5):494–504.

Thoreen, C. C. and Sabatini, D. M. (2005). Ampk and p53 help cells through lean times. *Cell metabolism*, 1(5):287–8.

Thu, K. L., Pikor, L. A., Chari, R., Wilson, I. M., Macaulay, C. E., English, J. C., Tsao, M.-S., Gazdar, A. F., Lam, S., Lam, W. L., and Lockwood, W. W. (2011). Genetic disruption of keap1/cul3 e3 ubiquitin ligase complex components is a key mechanism of nf-kappab pathway activation in lung cancer. *Journal of thoracic oncology : official publication of the International Association for the Study of Lung Cancer*, 6(9):1521–9.

Tian, X., Zhou, Y., Wang, Y., Zhang, S., Feng, J., Wang, X., Guo, H., Fan, R., Feng, N., Jia, M., Gu, X., Li, J., Yang, L., Wang, Y., Li, J., Zheng, G., Fu, F., and Pei, J. (2019). Mitochondrial dysfunction and apoptosis are attenuated on $\kappa$-opioid receptor activation through ampk/gsk-3$\beta$ pathway after myocardial ischemia and reperfusion. *Journal of cardiovascular pharmacology*, 73(2):70–81.

Timofeev, O., Cizmecioglu, O., Settele, F., Kempf, T., and Hoffmann, I. (2010). Cdc25 phosphatases are required for timely assembly of cdk1-cyclin b at the g2/m transition. *The Journal of biological chemistry*, 285(22):16978–90.

Tong, K., Kothari, O. A., Haro, K. S., Panda, A., Bandari, M. M., Carrick, J. N., Hur, J. J., Zhang, L., Chan, C. S., Xing, J., Gatza, M. L., Ganesan, S., and Verzi, M. P. (2021). Smad4 is critical in suppression of braf-v600e serrated tumorigenesis. *Oncogene*, 40(41):6034–6048.

Truong, L. N., Li, Y., Shi, L. Z., Hwang, P. Y.-H., He, J., Wang, H., Razavian, N., Berns, M. W., and Wu, X. (2013). Microhomology-mediated end joining and homologous recombination share the initial end resection step to repair dna double-strand breaks in mammalian cells. *Proceedings of the National Academy of Sciences of the United States of America*, 110(19):7720–5.

Uziel, T., Lerenthal, Y., Moyal, L., Andegeko, Y., Mittelman, L., and Shiloh, Y. (2003). Requirement of the mrn complex for atm activation by dna damage. *The EMBO journal*, 22(20):5612–21.

VanArsdale, T., Boshoff, C., Arndt, K. T., and Abraham, R. T. (2015). Molecular pathways: Targeting the cyclin d-cdk4/6 axis for cancer treatment. *Clinical cancer research : an official journal of the American Association for Cancer Research*, 21(13):2905–10.

Vigo, E., Müller, H., Prosperini, E., Hateboer, G., Cartwright, P., Moroni, M. C., and Helin, K. (1999). Cdc25a phosphatase is a target of e2f and is required for efficient e2f-induced s phase. *Molecular and cellular biology*, 19(9):6379–95.

Watanabe, N., Arai, H., Nishihara, Y., Taniguchi, M., Watanabe, N., Hunter, T., and Osada, H. (2004). M-phase kinases induce phospho-dependent ubiquitination of somatic wee1 by scfbeta-trcp. *Proceedings of the National Academy of Sciences of the United States of America*, 101(13):4419–24.

Wee, P. and Wang, Z. (2017). Epidermal growth factor receptor cell proliferation signaling pathways. *Cancers*, 9(5).

Weerasinghe, P., Garcia, G. E., Zhu, Q., Yuan, P., Feng, L., Mao, L., and Jing, N. (2007). Inhibition of stat3 activation and tumor growth suppression of non-small cell lung cancer by g-quartet oligonucleotides. *International journal of oncology*, 31(1):129–36.

Wei, D. and Sun, Y. (2010). Small ring finger proteins rbx1 and rbx2 of scf e3 ubiquitin ligases: The role in cancer and as cancer targets. *Genes & cancer*, 1(7):700–7.

Wells, A. (1999). Egf receptor. *The international journal of biochemistry & cell biology*, 31(6):637–43.

Wroblewski, D., Jiang, C. C., Croft, A., Farrelly, M. L., Zhang, X. D., and Hersey, P. (2013). Obatoclax and abt-737 induce er stress responses in human melanoma cells that limit induction of apoptosis. *PloS one*, 8(12):e84073.

Xing, Y., Clements, W. K., Kimelman, D., and Xu, W. (2003). Crystal structure of a beta-catenin/axin complex suggests a mechanism for the beta-catenin destruction complex. *Genes & development*, 17(22):2753–64.

Xu, X., Li, D., Liu, J., Ma, Z., Huang, H., Min, L., Dai, L., and Dong, S. (2019). Downregulation of ptprk promotes cell proliferation and metastasis of nsclc by enhancing stat3 activation. *Analytical cellular pathology (Amsterdam)*, 2019:4265040.

Yang, T., Zeng, Y., Li, Y., Huang, Y., Meng, X., Lu, C., Zhu, C., Liu, P., Liu, J., Hao, S., Luo, J., Cui, W., and Yang, X. (2025). Par6/sox2 interact to modulate stemness maintenance in glioma by regulating the egfr/pi3k/akt signaling cascade. *Oncogene*, 44(46):4441–4461.

Yang, Y., Ludwig, R. L., Jensen, J. P., Pierre, S. A., Medaglia, M. V., Davydov, I. V., Safiran, Y. J., Oberoi, P., Kenten, J. H., Phillips, A. C., Weissman, A. M., and Vousden, K. H. (2005). Small molecule inhibitors of hdm2 ubiquitin ligase activity stabilize and activate p53 in cells. *Cancer cell*, 7(6):547–59.

You, H., Pellegrini, M., Tsuchihara, K., Yamamoto, K., Hacker, G., Erlacher, M., Villunger, A., and Mak, T. W. (2006). Foxo3a-dependent regulation of puma in response to cytokine/growth factor withdrawal. *The Journal of experimental medicine*, 203(7):1657–63.

Zhang, F., Fan, Q., Ren, K., and Andreassen, P. R. (2009a). Palb2 functionally connects the breast cancer susceptibility proteins brca1 and brca2. *Molecular cancer research : MCR*, 7(7):1110–8.

Zhang, F., Ma, J., Wu, J., Ye, L., Cai, H., Xia, B., and Yu, X. (2009b). Palb2 links brca1 and brca2 in the dna-damage response. *Current biology : CB*, 19(6):524–9.

Zhang, X., Guo, A., Yu, J., Possemato, A., Chen, Y., Zheng, W., Polakiewicz, R. D., Kinzler, K. W., Vogelstein, B., Velculescu, V. E., and Wang, Z. J. (2007). Identification of stat3 as a substrate of receptor protein tyrosine phosphatase t. *Proceedings of the National Academy of Sciences of the United States of America*, 104(10):4060–4.

Zhang, Y.-Q., Xiao, C.-X., Lin, B.-Y., Shi, Y., Liu, Y.-P., Liu, J.-J., Guleng, B., and Ren, J.-L. (2013). Silencing of pokemon enhances caspase-dependent apoptosis via fas- and mitochondria-mediated pathways in hepatocellular carcinoma cells. *PloS one*, 8(7):e68981.

Zhang, Z., Luo, D., Xie, J., Lin, G., Zhou, J., Liu, W., Li, H., Yi, T., Su, Z., and Chen, J. (2018). Octahydrocurcumin, a final hydrogenated metabolite of curcumin, possesses superior anti-tumor activity through induction of cellular apoptosis. *Food & function*, 9(4):2005–2014.

Zhou, H., Cai, Y., Liu, D., Li, M., Sha, Y., Zhang, W., Wang, K., Gong, J., Tang, N., Huang, A., and Xia, J. (2018). Pharmacological or transcriptional inhibition of both hdac1 and 2 leads to cell cycle blockage and apoptosis via p21waf1/cip1 and p19ink4d upregulation in hepatocellular carcinoma. *Cell proliferation*, 51(3):e12447.

Zhou, Y., Tang, G., Li, X., Sun, W., Liang, Y., Gan, D., Liu, G., Song, W., and Wang, Z. (2020). Study on the chemical constituents of nut oil from prunus mira koehne and the mechanism of promoting hair growth. *Journal of ethnopharmacology*, 258:112831.

Zhu, G., Cheng, Z., Huang, Y., Zheng, W., Yang, S., Lin, C., and Ye, J. (2019). Traf6 promotes the progression and growth of colorectal cancer through nuclear shuttle regulation nf-kb/c-jun signaling pathway. *Life sciences*, 235:116831.

Zimmermann, M., Lottersberger, F., Buonomo, S. B., Sfeir, A., and de Lange, T. (2013). 53bp1 regulates dsb repair using rif1 to control 5' end resection. *Science (New York, N.Y.)*, 339(6120):700–4.

Zindy, F., Eischen, C. M., Randle, D. H., Kamijo, T., Cleveland, J. L., Sherr, C. J., and Roussel, M. F. (1998). Myc signaling via the arf tumor suppressor regulates p53-dependent apoptosis and immortalization. *Genes & development*, 12(15):2424–33.

Zörnig, M. and Evan, G. I. (1996). Cell cycle: on target with myc. *Current biology : CB*, 6(12):1553–6.
