# Supplementary material for: Virtual Tumors Enable Prediction of Personalized Therapeutic Combinations for Non-Small Cell Lung Cancer
Source: Cancer Res. Author manuscript; Available in PMC 2026 Aug 11. (PMC7619348; doi:10.1158/0008-5472.CAN-25-2476)
Supplement: Supplementary file 12 [file EMS216192-supplement-21.pdf]

Supplementary Table S3. Summary of the nodes included in the model

Model node name	Gene symbol	Full protein name
14-3-3	YWHAZ	14-3-3 protein
4EBP1	EIF4EBP1	4E-Binding Protein 1
53BP1	TP53BP1	p53 Binding Protein 1
Akt	AKT1	Protein Kinase B
AMPK	PRKAA1/PRKAA2	AMP-activated protein kinase
APC	APC	Adenomatous Polyposis Coli
ARF	CDKN2A	ADP Ribosylation Factor
ATM	ATM	Ataxia Telangiectasia Mutated
ATM-Control	ATM	Ataxia Telangiectasia Mutated (Control)
ATM-deficient	ATM	Ataxia Telangiectasia Mutated (Deficient)
ATR	ATR	Ataxia Telangiectasia and Rad3 related
BAD	BAD	Bcl-2-associated death promoter
BAKBAX	BAK1/BAX	Bcl-2 homologous antagonist killer/Bcl-2-associated X protein
baseline		Baseline
BCL-2	BCL2	B-cell lymphoma 2
BCL-W	BCL2L2	Bcl-2-like protein 2
BCL-XL	BCL2L1	B-cell lymphoma-extra large
Beta-Catenin	CTNNB1	Beta-Catenin
Beta-TRCP	BTRC	Beta-transducin repeat-containing protein
Bim	BCL2L11	Bcl-2-like protein 11
BRCA1	BRCA1	Breast cancer type 1 susceptibility protein
BRCA2	BRCA2	Breast cancer type 2 susceptibility protein
c-Jun	JUN	Jun proto-oncogene
CBF	RUNX1	Core-binding factor subunit alpha-1
CDC25A	CDC25A	Cell division cycle 25A
CDC25C	CDC25C	Cell division cycle 25C
CDK1	CDK1	Cyclin-dependent kinase 1
CDK2	CDK2	Cyclin-dependent kinase 2
CDK4	CDK4	Cyclin-dependent kinase 4
CDKN2A	CDKN2A	Cyclin-dependent kinase inhibitor 2A
CHK1	CHEK1	Checkpoint kinase 1
CHK2	CHEK2	Checkpoint kinase 2
CtIP	RBBP8	RBBP8 (CtIP)
CUL3	CUL3	Cullin 3
CycB	CCNB1	Cyclin B1
CycD	CCND1	Cyclin D1
CycE	CCNE1	Cyclin E1
Dishevelled	DVL1/DVL2/DVL3	Dishevelled segment polarity protein
DNA-PKcs	PRKDC	DNA-dependent protein kinase catalytic subunit
E2F	E2F1	E2F transcription factor 1
EGF	EGF	Epidermal growth factor
EGFR	EGFR	Epidermal growth factor receptor
ELF3	ELF3	E74-like factor 3
Erbb1	ERBB1	Erb-b2 receptor tyrosine kinase 1
Erbb3	ERBB3	Erb-b2 receptor tyrosine kinase 3
ERK	MAPK1/MAPK3	Extracellular signal-regulated kinase
FOXM1	FOXM1	Forkhead box protein M1
FOXO3	FOXO3	Forkhead box protein O3
Frizzled	FZD1-FZD10	Frizzled class receptor
Fyn	FYN	Fyn proto-oncogene
GRB2	GRB2	Growth factor receptor-bound protein 2

Model node name	Gene symbol	Full protein name
GSK3B-AXIN-CK1-APC	GSK3B/AXIN1/CSNK1A1/APC	Glycogen synthase kinase 3 beta/Axin 1/Casein kinase 1 alpha 1/Adenomatous polyposis coli
GSK3B	GSK3B	Glycogen synthase kinase 3 beta
HGF	HGF	Hepatocyte growth factor
hTERT	TERT	Telomerase reverse transcriptase
IL-6	IL6	Interleukin 6
IL-R6	IL6R	Interleukin 6 receptor
IR	INSR	Insulin receptor
IR-FLIP		Irradiation flipping node
JAK2	JAK2	Janus kinase 2
KEAP1-CUL3-RBX1	KEAP1/CUL3/RBX1	Kelch-like ECH-associated protein 1/Cullin 3/Ring-box 1
KEAP1	KEAP1	Kelch-like ECH-associated protein 1
KU70	XRCC6	X-ray repair cross-complementing protein 6
KU80	XRCC5	X-ray repair cross-complementing protein 5
LIG4	LIG4	DNA ligase 4
LRP	LRP1	LDL receptor-related protein 1
LZTR1	LZTR1	Leucine-zipper-like transcription regulator 1
MAX	MAX	Myc-associated factor X
MCL-1	MCL1	Induced myeloid leukemia cell differentiation protein
MDC1	MDC1	Mediator of DNA damage checkpoint 1
MDM2	MDM2	Mouse double minute 2 homolog
MEK	MAP2K1/MAP2K2	Mitogen-activated protein kinase kinase 1/2
MET	MET	MET proto-oncogene
MGA	MGA	MAX gene-associated
MOMP	BAX/BAK1	Mitochondrial outer membrane permeabilization (Bcl-2 homologous antagonist killer/Bcl-2-associated X protein)
MRE11	MRE11	MRE11 homolog
MRN	MRE11/RAD50/NBN	MRE11-RAD50-NBS1 complex
mTORC1	MTOR	Mechanistic target of rapamycin complex 1
mTORC2	MTOR	Mechanistic target of rapamycin complex 2
MUC1	MUC1	Mucin 1
MYC	MYC	Myc proto-oncogene
MycAmp	MYC	Myc proto-oncogene amplification
NBS1	NBN	Nijmegen breakage syndrome 1
NCID	NOTCH1	Notch intracellular domain
NF1	NF1	Neurofibromin 1
NFkB	NFKB1/NFKB2/RELA	Nuclear factor kappa-light-chain-enhancer
NOTCH1	NOTCH1	Neurogenic locus notch homolog protein 1
NOXA	PMAIP1	Phorbol-12-myristate-13-acetate-induced protein 1
NRF2	NFE2L2	Nuclear factor erythroid 2-related factor 2
p15	CDKN2B	Cyclin-dependent kinase inhibitor 2B
p16	CDKN2A	Cyclin-dependent kinase inhibitor 2A
p21	CDKN1A	Cyclin-dependent kinase inhibitor 1A
p27	CDKN1B	Cyclin-dependent kinase inhibitor 1B
p27-amp	CDKN1B	Cyclin-dependent kinase inhibitor 1B amplification
p53	TP53	Tumor protein p53
p70S6K	RPS6KB1/RPS6KB2	Ribosomal protein S6 kinase beta-1/2
p90RSK	RPS6KA1/RPS6KA3	Ribosomal protein S6 kinase alpha-1/3
PALB2	PALB2	Partner and localizer of BRCA2
PI3K	PIK3CA/PIK3CB/PIK3CD/PIK3C	Phosphoinositide 3-kinase catalytic subunits
PIK3CA	PIK3CA	Phosphoinositide 3-kinase catalytic subunit alpha
PKA	PRKACA/PRKACB/PRKACG	Protein kinase A catalytic subunits
Plk1	PLK1	Polo-like kinase 1
pRB	RB1	Retinoblastoma protein
PTEN	PTEN	Phosphatase and tensin homolog

Model node name	Gene symbol	Full protein name
PTPRK	PTPRK	Protein tyrosine phosphatase receptor type K
PTPRT	PTPRT	Protein tyrosine phosphatase receptor type T
PUMA	BBC3	Bcl-2-binding component 3
RAD50	RAD50	RAD50 homolog
RAD51	RAD51	RAD51 recombinase
RAF	RAF1	Raf-1 proto-oncogene
RAP80	UIMC1	Ubiquitin interaction motif containing 1
RAS	HRAS/KRAS/NRAS	Harvey/Kirsten/Neuroblastoma RAS viral oncogene homolog
RASSF1	RASSF1	Ras association domain family member 1
RBX1	RBX1	Ring-box 1
RNF168	RNF168	Ring finger protein 168
RNF8	RNF8	Ring finger protein 8
seDSB		Single ended double stanrd break
SFRP4	SFRP4	Secreted frizzled-related protein 4
SKP2	SKP2	S-phase kinase-associated protein 2
SOS	SOS1/SOS2	Son of sevenless homolog 1/2
SSB	SSBP1	Single-stranded DNA-binding protein 1
STAT3	STAT3	Signal transducer and activator of transcription 3
STIL	STIL	SCL/TAL1 interrupting locus
STK11	STK11	Serine/threonine kinase 11
TCF	TCF7/TCF7L1/TCF7L2	T-cell factor 7/7-like 1/7-like 2
toxic-NHEJ		Toxic non-homologous end joining
TSC	TSC1/TSC2	Tuberous sclerosis complex 1/2
Twist	TWIST1	Twist family bHLH transcription factor 1
UrseDSB		Unresolved double-stranded break
Wee1	WEE1	WEE1 G2 checkpoint kinase
WNT	WNT1-WNT10B	Wingless-type MMTV integration site family
XLF	NHEJ1	Non-homologous end joining factor 1
XRCC4	XRCC4	X-ray repair cross-complementing protein 4
YB1	YBX1	Y-box binding protein 1
ZNRF3	ZNRF3	Zinc and ring finger 3

Supplementary Table S3. Summary of the nodes included in the model. Summary of all the nodes, with corresponding gene symbols of the proteins involved where applicable.
