# Supplementary material for: Virtual Tumors Enable Prediction of Personalized Therapeutic Combinations for Non-Small Cell Lung Cancer
Source: Cancer Res. Author manuscript; Available in PMC 2026 Aug 11. (PMC7619348; doi:10.1158/0008-5472.CAN-25-2476)
Supplement: Supplementary file 13 [file EMS216192-supplement-22.pdf]

Supplementary Table S4. List of mutations used to model cell lines.

Cell line	Mutated Gene	Model Value	Mutation	Mutation Function
HCC44	STIL	3	p.G487fs*7	Ambiguous function
HCC44	LZTR1	0	CNA loss	Loss of Function
HCC44	p53	0	p.R175L	Gain of Function
HCC44	RAS	3	p.G12C	Gain of Function
NCIH1373	ATM_deficient	1	p.E2943*	Loss of Function
NCIH1373	CDKN2A	0	p.C72*	Loss of Function
NCIH1373	CUL3	0	p.E375*	Loss of Function
NCIH1373	RAS	3	p.G12C	Gain of Function
NCIH1373	SFRP4	0	CNA loss	Loss of Function
NCIH23	PTPRT	0	p.G495V	Loss of Function
NCIH23	LRP	0	p.R4084L	Loss of Function
NCIH23	STK11	0	p.W332*	Loss of Function
NCIH23	p53	0	p.M246I	Ambiguous function
NCIH23	RAS	3	p.G12C	Gain of Function
SW1573	PI3K	3	p.K111E	Gain of Function
SW1573	RAS	3	p.G12C	Gain of Function
SW1573	CDKN2A	0	CNA loss	Loss of Function
SW1573	Beta-Catenin	3	p.S33F	Gain of Function
NCIH358	ELF3	0	p.D239fs*62	Loss of Function
NCIH358	PTPRK	0	CNA loss	Loss of Function
NCIH358	RAS	3	p.G12C	Gain of Function
NCIH358	p53	0	Deletion	Loss of Function
NCIH358	LRP	0	p.S878*	Loss of Function
NCIH1792	CDKN2A	0	CNA loss	Loss of Function
NCIH1792	RAS	3	p.G12C	Gain of Function
NCIH1792	p53	0	CAN loss	Loss of Function
NCIH1792	Erbb3	0	p.V104 R106delVVR	Loss of Function
A549	RAS	3	p.G12S	Gain of Function
A549	STK11	0	CNA loss	Loss of Function
A549	KEAP1	0	CNA loss	Loss of Function
A549	CDKN2A	0	Deletion	Loss of Function
A549	ATR	0	Splice Variant	Loss of Function
A549	ARF	0	Deletion	Loss of Function
PC9	Erbb1	3	CNA gain	Gain of Function
PC9	p53	0	p.R248Q	Loss of Function
PC9	CDKN2A	0	G67V	Loss of Function
HCC827	Erbb1	3	p.E746K	Gain of Function
HCC827	p53	0	p.V218L	Loss of Function
H1993	p53	0	p.C242W	Loss of Function
H1993	STK11	0	p.E199*	Loss of Function
H1993	MET	3	Amplification	Gain of Function

Supplementary Table S4. List of mutations used to model cell lines. For each simulated cell line, the constrained nodes and their value are given, representing oncogenic mutations known to present in the cell line. A value of 0 indicates a loss-of-function mutation conferring no activity and for 1 basal activity, 2 elevated activity and 3 strong activation for gain-of-function mutations. Exact mutations and their functions were extracted from Cell Model Passport (van der Meer et al., 2019).

Supplementary References

van der Meer, D., Barthorpe, S., Yang, W., Lightfoot, H., Hall, C., Gilbert, J., Francies, H. E., and Garnett, M. J. (2019). Cell Model Passports-a hub for clinical, genetic and functional datasets of preclinical cancer models. *Nucleic Acids Research*, 47(D1):D923–D929.
