# Supplementary material for: Virtual Tumors Enable Prediction of Personalized Therapeutic Combinations for Non-Small Cell Lung Cancer
Source: Cancer Res. Author manuscript; Available in PMC 2026 Aug 11. (PMC7619348; doi:10.1158/0008-5472.CAN-25-2476)
Supplement: Supplementary file 14 [file EMS216192-supplement-23.pdf]

Supplementary Table S5. Experiments from the literature used to verify the *in silico* model.

Source	Experiment	Cell line	Mutations	Constraints	Expected results	Model results
IR cell line sensitivity from Hannon lab	IR	NCIH1373	CDKN2A OFF, PTPRK OFF, SFRP4 OFF, CUL3 OFF, p53 OFF, RAS Very high, ATM_deficient Low	IR_FLIP OFF	Death Low	Death Low
	No IR	NCIH1373	CDKN2A OFF, PTPRK OFF, SFRP4 OFF, CUL3 OFF, p53 OFF, RAS Very high, ATM_deficient Low		IR_FLIP Very high, Death Low	IR_FLIP Very high, Death Low
	IR	NCIH1792	CDKN2A OFF, RAS Very high, p53 OFF, Erbb3 OFF	IR_FLIP OFF	Death Low	Death Low
	No IR	NCIH1792	CDKN2A OFF, RAS Very high, p53 OFF, Erbb3 OFF		IR_FLIP Very high, Death Low	IR_FLIP Very high, Death Low
	IR	NCIH358	ELF3 OFF, LRP OFF, p53 OFF, PTPRK OFF, RAS Very high	IR_FLIP OFF	Death Low	Death Low
	No IR	NCIH358	ELF3 OFF, LRP OFF, p53 OFF, PTPRK OFF, RAS Very high		IR_FLIP Very high, Death Low	IR_FLIP Very high, Death Low
	IR	HCC44	LZTR1 OFF, p53 OFF, RAS Very high, STIL Very high	IR_FLIP OFF	Death Low	Death Low
	No IR	HCC44	LZTR1 OFF, p53 OFF, RAS Very high, STIL Very high		IR_FLIP Very high, Death Low	IR_FLIP Very high, Death Low
	IR	NCIH2291	MGA OFF, PTPRK OFF, p53 OFF, RAS Very high	IR_FLIP OFF	Death Low	Death Low
	No IR	NCIH2291	MGA OFF, PTPRK OFF, p53 OFF, RAS Very high		IR_FLIP Very high, Death Low	IR_FLIP Very high, Death Low
	IR	SW1573	PI3K Very high, RAS Very high, Beta-Catenin Very high, CDKN2A OFF	IR_FLIP OFF	Death Low	Death Low
	No IR	SW1573	PI3K Very high, RAS Very high, Beta-Catenin Very high, CDKN2A OFF		IR_FLIP Very high, Death Low	IR_FLIP Very high, Death Low
	No IR	NCIH23	PTPRT OFF, ATM_deficient Low, LRP OFF, STK11 OFF, p53 OFF, RAS Very high		IR_FLIP Very high, Death Low	IR_FLIP Very high, Death Low
	IR	NCIH23	PTPRT OFF, LRP OFF, STK11 OFF, p53 OFF, RAS Very high, ATM_deficient Low	IR_FLIP OFF	Death Very high	Death Low
	IR	NCIH2122	STK11 OFF, RAS Very high, p53 OFF, KEAP1 OFF	IR_FLIP OFF	Death Very high	Death Low
	No IR	NCIH2122	STK11 OFF, RAS Very high, p53 OFF, KEAP1 OFF		IR_FLIP Very high, Death Low	IR_FLIP Very high, Death Low
Minjgee et al. 2011	Control, no KRAS(V12)	FaDu	CDKN2A OFF, p53 OFF		RAS High, Akt Low	RAS High, Akt Low
	Test, KRAS(V12)	FaDu	CDKN2A OFF, p53 OFF	RAS Very high	Akt High	Akt High
Zhang et al. 2007	Control, scrambled siRNA	MCF-7	NBS1 OFF, PIK3CA Very high		PTPRT Low, STAT3 Low	PTPRT Low, STAT3 High
	Test, PTPRT siRNA	MCF-7	NBS1 OFF, PIK3CA Very high	PTPRT OFF	STAT3 Very high	STAT3 Very high
Wael et al. 2014	RNAi negative control	NCI-H2170	RAS High, KEAP1 OFF, CDKN2A OFF, p53 OFF		Proliferation High, Apoptosis Low, Akt Low	Proliferation High, Apoptosis OFF, Akt Low
	Test, NOTCH1 shRNA	NCI-H2170	RAS High, KEAP1 OFF, CDKN2A OFF, p53 OFF	NOTCH1 OFF	Proliferation High, Apoptosis Low, Akt Low	Proliferation High, Apoptosis OFF, Akt Low
	RNAi negative control	A549	RAS Very high, STK11 OFF, KEAP1 OFF, CDKN2A OFF, ATR OFF, ARF OFF		Proliferation High, Apoptosis Low, Akt Low	Proliferation High, Apoptosis Low, Akt Low
	Test, NOTCH1 shRNA	A549	RAS Very high, STK11 OFF, KEAP1 OFF, CDKN2A OFF, ATR OFF, ARF OFF	NOTCH1 OFF	Proliferation Very high, Apoptosis OFF, Akt High	Proliferation Very high, Apoptosis OFF, Akt High

Cortez et al. 2015	Control: no miR-34a, no ionising radiation	A549	RAS Very high, STK11 OFF, KEAP1 OFF, CDKN2A OFF, ATR OFF, ARF OFF		RAD51 Low, IR_FLIP Very high*, Death OFF	RAD51 Low, IR_FLIP Very high, Death Low
	Ionising radiation, no miR-34a	A549	RAS Very high, STK11 OFF, KEAP1 OFF, CDKN2A OFF, ATR OFF, ARF OFF	IR_FLIP OFF	RAD51 Very high*, Death Low	RAD51 Very high, Death High
	miR-34a, 4Gy ionising radiation	A549	RAS Very high, STK11 OFF, KEAP1 OFF, CDKN2A OFF, ATR OFF, ARF OFF	RAD51 OFF, IR_FLIP OFF	Death High	Death Very high
	miR-34a, no ionising radiation	A549	RAS Very high, STK11 OFF, KEAP1 OFF, CDKN2A OFF, ATR OFF, ARF OFF	RAD51 OFF	IR_FLIP Very high*, Death Low	IR_FLIP Very high, Death High
Llabata et al. 2020	Control, empty ZsGreen	A549	RAS Very high, STK11 OFF, KEAP1 OFF, CDKN2A OFF, ATR OFF, ARF OFF		MGA Low, Proliferation High, MYCMAX High*	MGA Low, Proliferation High, MYCMAX Very high
	Treat, MGA-ZsGreen overexpression vector	A549	RAS Very high, STK11 OFF, KEAP1 OFF, CDKN2A OFF, ATR OFF, ARF OFF	MGA Very high	MYCMAX OFF*, Proliferation OFF	MYCMAX OFF, Proliferation Low
Xu et al. 2017	Control, control-siRNA	NCI-H1650	APC OFF		Akt High, ERK High, Apoptosis Low, Proliferation Low	Akt Low, ERK High, Apoptosis Low, Proliferation Low
	Treat, MUC1-siRNA	NCI-H1650	APC OFF	MUC1 OFF	Akt Low, ERK Low, Apoptosis High, Proliferation OFF	Akt OFF, ERK OFF, Apoptosis High, Proliferation OFF
	Control, no JAK2 RNAi	A549	RAS Very high, STK11 OFF, KEAP1 OFF, CDKN2A OFF, ATR OFF, ARF OFF		Proliferation High, JAK2 High*	Proliferation High, JAK2 High
	Treat, JAK2 RNAi	A549	RAS Very high, STK11 OFF, KEAP1 OFF, CDKN2A OFF, ATR OFF, ARF OFF	JAK2 OFF	Proliferation Low	Proliferation Low
	Treat, flag-JAK2	A549	RAS Very high, STK11 OFF, KEAP1 OFF, CDKN2A OFF, ATR OFF, ARF OFF	JAK2 Very high	Proliferation Very high	Proliferation Very high
Yang et al. 2018	Control, no DNA-PKcs inhibitor, no IR	A549	RAS Very high, STK11 OFF, KEAP1 OFF, CDKN2A OFF, ATR OFF, ARF OFF		IR_FLIP Very high*, DSB Low, Death OFF	IR_FLIP Very high, DSB Low, Death Low
	DNA-PKcs inhibition, IR	A549	RAS Very high, STK11 OFF, KEAP1 OFF, CDKN2A OFF, ATR OFF, ARF OFF	IR_FLIP OFF	DSB Very high, Death Very high	DSB Very high, Death Very high
	DNA-PKcs inhibition, no IR	A549	RAS Very high, STK11 OFF, KEAP1 OFF, CDKN2A OFF, ATR OFF, ARF OFF		IR_FLIP Very high*, DSB Low, Death OFF	IR_FLIP Very high, DSB Low, Death Low
	IR, no DNA-PKcs inhibition	A549	RAS Very high, STK11 OFF, KEAP1 OFF, CDKN2A OFF, ATR OFF, ARF OFF	IR_FLIP OFF	DSB Very high, Death High	DSB Very high, Death High
Wang et al. 2018	Control, no shELF3	A549	RAS Very high, STK11 OFF, KEAP1 OFF, CDKN2A OFF, ATR OFF, ARF OFF		ELF3 Low, PI3K Low, MEK Very high	ELF3 Low, PI3K Low, MEK Very high
	Test, shELF3	A549	RAS Very high, STK11 OFF, KEAP1 OFF, CDKN2A OFF, ATR OFF, ARF OFF	ELF3 OFF	PI3K Low, MEK Low	PI3K Low, MEK High
Edelmann et al. 2005	Cells irradiated with 10 Gy after 4 days	BEAS-2B	EGF High	IR_FLIP OFF	Death High	Death High
	Control	BEAS-2B	EGF High	IR_FLIP Very high	Death Low	Death Low
Sato et al. 2020	Control	HBEC3	EGF High		Proliferation Low, p21 High	Proliferation Low, p21 Low
	KRASV12 transfected	HBEC3	EGF High	RAS Very high	Proliferation High, p21 High	Proliferation High, p21 High
	p53RNAi	HBEC3	EGF High	p53 OFF	Proliferation High, p21 OFF	Proliferation Low, p21 OFF
	p53RNAi and KRASV12 transfected	HBEC3	EGF High	RAS Very high, p53 OFF	Proliferation Very high, p21 OFF	Proliferation Very high, p21 OFF

Fok et al. 2019	Control	A549	RAS Very high, STK11 OFF, KEAP1 OFF, CDKN2A OFF, ATR OFF, ARF OFF		IR_FLIP Very high,* Death OFF	IR_FLIP Very high, Death Low
	Test, AZD7648	A549	RAS Very high, STK11 OFF, KEAP1 OFF, CDKN2A OFF, ATR OFF, ARF OFF		IR_FLIP Very high,* Death Low	IR_FLIP Very high, Death Low
	Test, AZD7648 plus IR 4Gy	A549	RAS Very high, STK11 OFF, KEAP1 OFF, CDKN2A OFF, ATR OFF, ARF OFF	IR_FLIP OFF	Death Very high	Death Very high
	Test, IR 4Gy	A549	RAS Very high, STK11 OFF, KEAP1 OFF, CDKN2A OFF, ATR OFF, ARF OFF	IR_FLIP OFF	Death Low	Death High
Zhao et al. 2016	Control, no Gefitinib	A549	RAS Very high, STK11 OFF, KEAP1 OFF, CDKN2A OFF, ATR OFF, ARF OFF		Erbb1 High,* Apoptosis OFF, Proliferation High, PI3K Low, Akt Low, mTORC1 High, mTORC2 Low	Erbb1 High, Apoptosis Low, Proliferation High, PI3K Low, Akt Low, mTORC1 High, mTORC2 Low
	Treat, Gefitinib	A549	RAS Very high, STK11 OFF, KEAP1 OFF, CDKN2A OFF, ATR OFF, ARF OFF	Erbb1 OFF	Apoptosis High, Proliferation OFF, PI3K OFF, Akt OFF, mTORC1 Low, mTORC2 Low	Apoptosis Low, Proliferation Low, PI3K OFF, Akt OFF, mTORC1 High, mTORC2 OFF
	Treat, Gefitinib and LY294002	A549	RAS Very high, STK11 OFF, KEAP1 OFF, CDKN2A OFF, ATR OFF, ARF OFF	Erbb1 OFF, PI3K OFF	Apoptosis High	Apoptosis High
	Treat, LY294002	A549	RAS Very high, STK11 OFF, KEAP1 OFF, CDKN2A OFF, ATR OFF, ARF OFF	Erbb1 OFF, PI3K OFF	Apoptosis High	Apoptosis High
Gao et al. 2017	Control, no COMPOUND7809	A549	RAS Very high, STK11 OFF, KEAP1 OFF, CDKN2A OFF, ATR OFF, ARF OFF		EGFR High,* Proliferation High, Apoptosis OFF*	EGFR High, Proliferation High, Apoptosis Low
	Treat, COMPOUND7809	A549	RAS Very high, STK11 OFF, KEAP1 OFF, CDKN2A OFF, ATR OFF, ARF OFF	EGFR OFF	Proliferation OFF, Apoptosis Low*	Proliferation Low, Apoptosis Low
	Control, no COMPOUND7809	SK-LU-1	p53 OFF, RAS Very high, APC OFF		EGFR High, Proliferation High, Apoptosis OFF*	EGFR High, Proliferation Very high, Apoptosis OFF
	Treat, COMPOUND7809	SK-LU-1	p53 OFF, RAS Very high, APC OFF	EGFR OFF	Proliferation Low, Apoptosis Low*	Proliferation High, Apoptosis OFF
	Control, no COMPOUND7809	NCIH23	p53 OFF, RAS Very high, STK11 OFF, LRP OFF, ATM_deficient Low		EGFR High, Proliferation High, Apoptosis OFF*	EGFR Low, Proliferation High, Apoptosis Low
	Treat, COMPOUND7809	NCIH23	p53 OFF, RAS Very high, STK11 OFF, LRP OFF, ATM_deficient Low	EGFR OFF	Proliferation Low, Apoptosis Low*	Proliferation Low, Apoptosis Low
Bigenzahn et al. 2018	Control, no LZTR1 sgRNA	K-562	p53 OFF, Akt Very high		LZTR1 Low, MEK High	LZTR1 Low, MEK High
	Test, LZTR1 sgRNA	K-562	p53 OFF, Akt Very high	LZTR1 OFF	MEK Very high	MEK High
Wang et al. 2017	4Gy IR, no KRASmut transfection	NCI-H1703	CDKN2A OFF, p53 OFF	IR_FLIP OFF	RAS High,* Death High	RAS High, Death High
	Control, no IR or KRASmut transfection	NCI-H1703	CDKN2A OFF, p53 OFF		RAS High,* IR_FLIP Very high,* Death Low	RAS High, IR_FLIP Very high, Death Low
	KRASmut transfection, no IR	NCI-H1703	CDKN2A OFF, p53 OFF	RAS Very high	IR_FLIP Very high,* Death Low	IR_FLIP Very high, Death Low
	KRASmut transfection, 4Gy IR	NCI-H1703	CDKN2A OFF, p53 OFF	RAS Very high, IR_FLIP OFF	Death Low	Death Low
Zhou et al. 2013	Control	A549	RAS Very high, STK11 OFF, KEAP1 OFF, CDKN2A OFF, ATR OFF, ARF OFF		IR_FLIP Very high,* RNF8 Low, Death OFF, RAD51 Low, G2_M_arrest Low	IR_FLIP Very high, RNF8 Low, Death Low, RAD51 Low, G2_M_arrest Low
	IR (2Gy)	A549	RAS Very high, STK11 OFF, KEAP1 OFF, CDKN2A OFF, ATR OFF, ARF OFF	IR_FLIP OFF	RNF8 Very high, Death High, RAD51 Very high, G2_M_arrest High	RNF8 Very high, Death High, RAD51 Very high, G2_M_arrest Low
	IR (2Gy), Lenti-siRNF8	A549	RAS Very high, STK11 OFF, KEAP1 OFF, CDKN2A OFF, ATR OFF, ARF OFF	IR_FLIP OFF, RNF8 OFF	Death Very high, RAD51 Low, G2_M_arrest High	Death Very high, RAD51 OFF, G2_M_arrest Low

	Lenti-siRNF8	A549	RAS Very high, STK11 OFF, KEAP1 OFF, CDKN2A OFF, ATR OFF, ARF OFF	RNF8 OFF	IR_FLIP Very high,* Death OFF, RAD51 Low, G2_M_arrest High	IR_FLIP Very high, Death Low, RAD51 OFF, G2_M_arrest Low
Francesco et al. 2013	2Gy ionising radiation, no miR-27a	A549	RAS Very high, STK11 OFF, KEAP1 OFF, CDKN2A OFF, ATR OFF, ARF OFF	IR_FLIP OFF	ATM Very high, G2_M_arrest Low, Proliferation High, Death Very high	ATM Very high, G2_M_arrest Low, Proliferation High, Death High
	Control, no miR-27a, no ionising radiation	A549	RAS Very high, STK11 OFF, KEAP1 OFF, CDKN2A OFF, ATR OFF, ARF OFF		ATM Low, IR_FLIP Very high,* Proliferation High, Death OFF	ATM Low, IR_FLIP Very high, Proliferation High, Death Low
	miR27a, 2Gy ionising radiation	A549	RAS Very high, STK11 OFF, KEAP1 OFF, CDKN2A OFF, ATR OFF, ARF OFF	ATM OFF, IR_FLIP OFF	G2_M_arrest OFF, Proliferation Very high, Death High	G2_M_arrest OFF, Proliferation Very high, Death Very high
	miR27a, no 2Gy ionising radiation	A549	RAS Very high, STK11 OFF, KEAP1 OFF, CDKN2A OFF, ATR OFF, ARF OFF	ATM OFF	IR_FLIP Very high,* Proliferation Very high, Death OFF	IR_FLIP Very high, Proliferation Very high, Death High
You et al. 2004	Control, no anti-Wnt2 monoclonal antibody	A549	RAS Very high, STK11 OFF, KEAP1 OFF, CDKN2A OFF, ATR OFF, ARF OFF		WNT High, Caspase3 OFF, Apoptosis Low	WNT High, Caspase3 OFF, Apoptosis Low
	Test, treatment with anti-Wnt2 monoclonal antibody	A549	RAS Very high, STK11 OFF, KEAP1 OFF, CDKN2A OFF, ATR OFF, ARF OFF	WNT OFF	Caspase3 High, Apoptosis High	Caspase3 OFF, Apoptosis Low
Xu et al. 2019	Control, no PTPRK siRNA	A549	RAS Very high, STK11 OFF, KEAP1 OFF, CDKN2A OFF, ATR OFF, ARF OFF		PTPRK High, Proliferation High	PTPRK High, Proliferation High
	Test, PTPRK siRNA	A549	RAS Very high, STK11 OFF, KEAP1 OFF, CDKN2A OFF, ATR OFF, ARF OFF	PTPRK OFF	Proliferation Very high	Proliferation Very high
Dorr et al. 2015	Control, no CUL3 siRNA	A549	RAS Very high, STK11 OFF, CDKN2A OFF, ATR OFF, ARF OFF		CUL3 High, NRF2 High,* Proliferation High	CUL3 High, NRF2 Very high, Proliferation High
	Test, CUL3 siRNA	A549	RAS Very high, STK11 OFF, CDKN2A OFF, ATR OFF, ARF OFF	CUL3 OFF	NRF2 Very high,* Proliferation Very high	NRF2 Very high, Proliferation High
Homma et al. 2009	Control, no NRF2 siRNA	NCI-H292	ARF OFF, Erbb1 Very high		NRF2 High, Proliferation High, G2_M_arrest OFF, G1arrest OFF, pRB OFF, p21 OFF	NRF2 Very high, Proliferation High, G2_M_arrest OFF, G1arrest OFF, pRB OFF, p21 High
	Treat, NRF2 siRNA	NCI-H292	ARF OFF, Erbb1 Very high	NRF2 OFF	Proliferation Low, G2_M_arrest Low, G1arrest Low, pRB Low, p21 Low	Proliferation Low, G2_M_arrest Low, G1arrest Low, pRB Low, p21 Low
	Control, no NRF2 siRNA	A549	RAS Very high, STK11 OFF, KEAP1 OFF, CDKN2A OFF, ATR OFF, ARF OFF		NRF2 High, Proliferation High, G2_M_arrest OFF, G1arrest OFF, pRB OFF, p21 Low	NRF2 Very high, Proliferation High, G2_M_arrest Low, G1arrest OFF, pRB OFF, p21 High
	Treat, NRF2 siRNA	A549	RAS Very high, STK11 OFF, KEAP1 OFF, CDKN2A OFF, ATR OFF, ARF OFF	NRF2 OFF	Proliferation Low, G2_M_arrest High, G1arrest Low, pRB Low, p21 High	Proliferation Low, G2_M_arrest High, G1arrest Low, pRB Low, p21 Low
	Control, no trametinib	PC-9	Erbb1 Very high, CDKN2A OFF, p53 OFF		MEK High,* Akt High, MET High, ERK High, Caspase3 OFF, Apoptosis OFF, Proliferation High	MEK Very high, Akt High, MET High, ERK Very high, Caspase3 OFF, Apoptosis OFF, Proliferation Very high
	Treat, LY294002	PC-9	Erbb1 Very high, CDKN2A OFF, p53 OFF	PI3K OFF	Caspase3 OFF, Proliferation High	Caspase3 OFF, Proliferation High
	Treat, trametinib	PC-9	Erbb1 Very high, CDKN2A OFF, p53 OFF	MEK OFF	Akt Very high, MET Low, ERK OFF, Caspase3 OFF, Apoptosis OFF, Proliferation High	Akt Very high, MET High, ERK OFF, Caspase3 OFF, Apoptosis OFF, Proliferation Low

Chiba et al. 2016	Treat, trametinib, LY294002	PC-9	Erbb1 Very high, CDKN2A OFF, p53 OFF	MEK OFF, PI3K OFF	MET High*, ERK OFF, Proliferation OFF, Caspase3 High	MET High, ERK OFF, Proliferation OFF, Caspase3 High
	Control, no trametinib	HCC827	Erbb1 Very high, p53 OFF		MEK High*, Akt High, MET High, ERK High, Caspase3 OFF, Apoptosis OFF, Proliferation High	MEK Very high, Akt High, MET High, ERK Very high, Caspase3 OFF, Apoptosis OFF, Proliferation Very high
	Treat, LY294002	HCC827	Erbb1 Very high, p53 OFF	PI3K OFF	Caspase3 OFF, Proliferation High	Caspase3 OFF, Proliferation High
	Treat, trametinib	HCC827	Erbb1 Very high, p53 OFF	MEK OFF	Akt Very high, MET High*, ERK OFF, Caspase3 OFF, Apoptosis OFF, Proliferation High	Akt Very high, MET High, ERK OFF, Caspase3 OFF, Apoptosis OFF, Proliferation Low
	Treat, trametinib, LY294002	HCC827	Erbb1 Very high, p53 OFF	MEK OFF, PI3K OFF	MET High, ERK OFF, Caspase3 High, Proliferation OFF	MET High, ERK OFF, Caspase3 High, Proliferation OFF
	Control, no trametinib	HCC827CNXR	p53 OFF	MET Very high	MEK High*, Akt High, ERK High, Caspase3 OFF, Apoptosis OFF, Proliferation High	MEK Very high, Akt High, ERK Very high, Caspase3 OFF, Apoptosis OFF, Proliferation Very high
	Treat, trametinib	HCC827CNXR	p53 OFF	MET Very high, MEK OFF	Akt High, ERK OFF, Caspase3 Low, Apoptosis Low, Proliferation OFF	Akt Low, ERK OFF, Caspase3 Low, Apoptosis Low, Proliferation OFF
	Control, no trametinib	EBC-1	p53 OFF, NRF2 Very high	MET Very high	MEK High*, Erbb1 High, Akt High, ERK High, Caspase3 OFF, Apoptosis OFF, Proliferation Very high	MEK Very high, Erbb1 High, Akt High, ERK Very high, Caspase3 OFF, Apoptosis OFF, Proliferation Very high
	Treat, crizotinib	EBC-1	p53 OFF, NRF2 Very high	MET OFF	Caspase3 Low, Apoptosis Low, Proliferation Low	Caspase3 Low, Apoptosis Low, Proliferation Low
	Treat, trametinib	EBC-1	p53 OFF, NRF2 Very high	MET Very high, MEK OFF	Erbb1 Low, Akt High, ERK OFF, Caspase3 High, Apoptosis Low, Proliferation Low	Erbb1 High, Akt Low, ERK OFF, Caspase3 Low, Apoptosis Low, Proliferation OFF
	Treat, trametinib, crizotinib	EBC-1	p53 OFF, NRF2 Very high	MET OFF, MEK OFF	ERK OFF, Caspase3 High, Apoptosis High, Proliferation Low	ERK OFF, Caspase3 High, Apoptosis High, Proliferation OFF
	Control, no trametinib	H1993	p53 OFF, STK11 OFF	MET Very high	MEK High*, Erbb1 High, Akt High, ERK High, Caspase3 OFF, Apoptosis OFF, Proliferation High	MEK Very high, Erbb1 High, Akt High, ERK Very high, Caspase3 OFF, Apoptosis OFF, Proliferation Very high
	Treat, crizotinib	H1993	p53 OFF, STK11 OFF	MET OFF	ERK Low*, Caspase3 Low*, Apoptosis Low, Proliferation Low	ERK Low, Caspase3 Low, Apoptosis Low, Proliferation Low
	Treat, trametinib	H1993	p53 OFF, STK11 OFF	MET Very high, MEK OFF	Erbb1 Low, Akt High, ERK OFF, Caspase3 High, Apoptosis High, Proliferation OFF	Erbb1 High, Akt High, ERK OFF, Caspase3 Low, Apoptosis Low, Proliferation Low
	Treat, trametinib, crizotinib	H1993	p53 OFF, STK11 OFF	MEK OFF, MET OFF	ERK OFF*, Caspase3 High*, Apoptosis High, Proliferation OFF	ERK OFF, Caspase3 High, Apoptosis High, Proliferation OFF

Li et al. 2015	Treat, WB-308	NCI-H1975	Erbb1 OFF, APC OFF, ATR OFF, p53 OFF, TSC OFF		Akt OFF, ERK OFF, p21 Low, BCL-2 OFF, BAKBAX High, CycD OFF, Apoptosis High, Proliferation OFF	Akt OFF, ERK OFF, p21 High, BCL-2 OFF, BAKBAX High, CycD OFF, Apoptosis High, Proliferation OFF
	Control, no WB-308	NCI-H1975	Erbb1 Very high, APC OFF, ATR OFF, p53 OFF, TSC OFF		Akt High, ERK Very high, p21 High, BCL-2 High, BAKBAX Low, CycD High, Apoptosis OFF, Proliferation Very high	Akt High, ERK Very high, p21 OFF, BCL-2 Very high, BAKBAX OFF, CycD Very high, Apoptosis OFF, Proliferation Very high
	Control, no WB-308	PC-9	Erbb1 Very high, p53 OFF, CDKN2A OFF		Akt High, ERK Very high, p21 High, BCL-2 Very high, BAKBAX Low, CycD High, Caspase3 OFF, G2_M_arrest Low, Apoptosis OFF, Proliferation Very high	Akt High, ERK Very high, p21 OFF, BCL-2 Very high, BAKBAX OFF, CycD Very high, Caspase3 OFF, G2_M_arrest OFF, Apoptosis OFF, Proliferation Very high
	Control, no WB-308	A549	RAS Very high, STK11 OFF, KEAP1 OFF, CDKN2A OFF, ATR OFF, ARF OFF		EGFR High, Akt High, ERK Very high, p21 High, BCL-2 High, BAKBAX OFF, CycD High, Apoptosis OFF, Proliferation High	EGFR High, Akt Low, ERK Very high, p21 High, BCL-2 High, BAKBAX Low, CycD Very high, Apoptosis Low, Proliferation High
	Treat, WB-308	A549	RAS Very high, STK11 OFF, KEAP1 OFF, CDKN2A OFF, ATR OFF, ARF OFF	EGFR OFF	Akt Low, ERK High, p21 Low, BCL-2 Low, BAKBAX Low, CycD Low, Apoptosis Low, Proliferation OFF	Akt OFF, ERK Very high, p21 High, BCL-2 Low, BAKBAX Low, CycD Very high, Apoptosis Low, Proliferation Low
	Treat, WB-308	PC-9	p53 OFF, CDKN2A OFF	Erbb1 OFF	Akt OFF, ERK OFF, p21 Low, BCL-2 OFF, BAKBAX High, CycD OFF, G2_M_arrest High, Caspase3 High, Apoptosis High, Proliferation OFF	Akt OFF, ERK OFF, p21 Low, BCL-2 OFF, BAKBAX High, CycD OFF, G2_M_arrest Very high, Caspase3 High, Apoptosis High, Proliferation OFF
Wang et al. 2008	Control	A549	RAS Very high, STK11 OFF, KEAP1 OFF, CDKN2A OFF, ATR OFF, ARF OFF		Death Low	Death Low
	Nrf2 siRNA + doxorubicin	A549	RAS Very high, STK11 OFF, KEAP1 OFF, CDKN2A OFF, ATR OFF, ARF OFF	IR_FLIP OFF, NRF2 OFF	Death Very high	Death Very high
	Transient knockdown of Nrf2	A549	RAS Very high, STK11 OFF, KEAP1 OFF, CDKN2A OFF, ATR OFF, ARF OFF	NRF2 OFF	Death High	Death Low

Supplementary Table S5. Experiments from the literature used to validate the *in silico* model. For each experiment, the table shows the cell line used, the constraints applied to the model and the expected outcome, based on the experimental result. The final column shows the result of the model simulations. Cases marked (*) indicate where we have explicitly stated our assumptions about ambiguous aspects of the experimental setup, or added checks that the default state of the model is correct. Removing these cases reduces the QWK to 0.79. References for the experiments described in this table are provided collectively here: (Wang et al., 2008; Edelmann et al., 2005; Sato et al., 2020; Li et al., 2015; Fok et al., 2019; Wael et al., 2014; Minjgee et al., 2011; Wang et al., 2017; Zhang et al., 2007; Bigenzahn et al., 2018; Wang et al., 2018; Mateos-Gomez et al., 2015; Higgins et al., 2010; Zhou et al., 2013; Di Francesco et al., 2013; Cortez et al., 2015; Yang et al., 2018; You et al., 2004; Xu et al., 2019; Dorr et al., 2015; Llabata et al., 2020; Zhao et al., 2016; Gao et al., 2017; Xu et al., 2017b; Homma et al., 2009; Chiba et al., 2016; Xu et al., 2017a).

Table References

Bigenzahn, J. W., Collu, G. M., Kartnig, F., Pieraks, M., Vladimer, G. I., Heinz, L. X., Sedlyarov, V., Schischlik, F., Fauster, A., Rebsamen, M., Parapatics, K., Blomen, V. A., Müller, A. C., Winter, G. E., Kralovics, R., Brummelkamp, T. R., Mlodzik, M., and Superti-Furga, G. (2018). LZTR1 is a regulator of RAS ubiquitination and signaling. *Science (New York, N.Y.)*, 362(6419):1171–1177.

Chiba, M., Togashi, Y., Tomida, S., Mizuuchi, H., Nakamura, Y., Banno, E., Hayashi, H., Terashima, M., De Velasco, M. A., Sakai, K., Fujita, Y., Mitsudomi, T., and Nishio, K. (2016). MEK inhibitors against MET-amplified non-small cell lung cancer. *International Journal of Oncology*, 49(6):2236–2244.

Cortez, M. A., Valdecanas, D., Niknam, S., Peltier, H. J., Diao, L., Giri, U., Komaki, R., Calin, G. A., Gomez, D. R., Chang, J. Y., Heymach, J. V., Bader, A. G., and Welsh, J. W. (2015). In Vivo Delivery of miR-34a Sensitizes Lung Tumors to Radiation Through RAD51 Regulation. *Molecular Therapy. Nucleic Acids*, 4(12):e270.

Di Francesco, A., De Pittà, C., Moret, F., Barbieri, V., Celotti, L., and Mognato, M. (2013). The DNA-damage response to γ-radiation is affected by miR-27a in A549 cells. *International Journal of Molecular Sciences*, 14(9):17881–17896.

Dorr, C., Janik, C., Weg, M., Been, R. A., Bader, J., Kang, R., Ng, B., Foran, L., Landman, S. R., O'Sullivan, M. G., Steinbach, M., Sarver, A. L., Silverstein, K. A. T., Largaespada, D. A., and Starr, T. K. (2015). Transposon Mutagenesis Screen Identifies Potential Lung Cancer Drivers and CUL3 as a Tumor Suppressor. *Molecular cancer research: MCR*, 13(8):1238–1247.

Edelmann, M., Gamarra, F., da Silva, A. K., Hornung, V., Castro, M., Passlick, B., Huber, R. M., and Bergner, A. (2005). Cell cycle effects of radiation on human bronchial epithelium and lung carcinoma cells in monolayer cultures and a three-dimensional co-culture system. *Radiation Research*, 164(4 Pt 1):391–399.

Fok, J. H. L., Ramos-Montoya, A., Vazquez-Chantada, M., Wijnhoven, P. W. G., Follia, V., James, N., Farrington, P. M., Karmokar, A., Willis, S. E., Cairns, J., Nikkilä, J., Beattie, D., Lamont, G. M., Finlay, M. R. V., Wilson, J., Smith, A., O'Connor, L. O., Ling, S., Fawell, S. E., O'Connor, M. J., Hollingsworth, S. J., Dean, E., Goldberg, F. W., Davies, B. R., and Cadogan, E. B. (2019). AZD7648 is a potent and selective DNA-PK inhibitor that enhances radiation, chemotherapy and olaparib activity. *Nature Communications*, 10(1):5065.

Gao, J., Liang, Y., Zhang, D., Wang, Y., Yang, J., and Liu, H. (2017). A novel epidermal growth factor receptor inhibitor for treating lung cancer. *Tumour Biology: The Journal of the International Society for Oncodevelopmental Biology and Medicine*, 39(4):1010428317698358.

Higgins, G. S., Prevo, R., Lee, Y.-F., Helleday, T., Muschel, R. J., Taylor, S., Yoshimura, M., Hickson, I. D., Bernhard, E. J., and McKenna, W. G. (2010). A small interfering RNA screen of genes involved in DNA repair identifies tumor-specific radiosensitization by POLQ knockdown. *Cancer Research*, 70(7):2984–2993.

Homma, S., Ishii, Y., Morishima, Y., Yamadori, T., Matsuno, Y., Haraguchi, N., Kikuchi, N., Satoh, H., Sakamoto, T., Hizawa, N., Itoh, K., and Yamamoto, M. (2009). Nrf2 enhances cell proliferation and resistance to anticancer drugs in human lung cancer. *Clinical Cancer Research: An Official Journal of the American Association for Cancer Research*, 15(10):3423–3432.

Li, J., Deng, H., Hu, M., Fang, Y., Vaughn, A., Cai, X., Xu, L., Wan, W., Li, Z., Chen, S., Yang, X., Wu, S., and Xiao, J. (2015). Inhibition of non-small cell lung cancer (NSCLC) growth by a novel small molecular inhibitor of EGFR. *Oncotarget*, 6(9):6749–6761.

Llabata, P., Mitsuishi, Y., Choi, P. S., Cai, D., Francis, J. M., Torres-Diz, M., Udeshi, N. D., Golomb, L., Wu, Z., Zhou, J., Svinkina, T., Aguilera-Jimenez, E., Liu, Y., Carr, S. A., Sanchez-Cespedes, M., Meyerson, M., and Zhang, X. (2020). Multi-Omics Analysis Identifies MGA as a Negative Regulator of the MYC Pathway in Lung Adenocarcinoma. *Molecular cancer research: MCR*, 18(4):574–584.

Mateos-Gomez, P. A., Gong, F., Nair, N., Miller, K. M., Lazzerini-Denchi, E., and Sfeir, A. (2015). Mammalian polymerase θ promotes alternative NHEJ and suppresses recombination. *Nature*, 518(7538):254–257.

Minjgee, M., Toulany, M., Kehlbach, R., Giehl, K., and Rodemann, H. P. (2011). K-RAS(V12) induces autocrine production of EGFR ligands and mediates radioresistance through EGFR-dependent Akt signaling and activation of DNA-PKcs. *International Journal of Radiation Oncology, Biology, Physics*, 81(5):1506–1514.

Sato, M., Shay, J. W., and Minna, J. D. (2020). Immortalized normal human lung epithelial cell models for studying lung cancer biology. *Respiratory Investigation*, 58(5):344–354.

Wael, H., Yoshida, R., Kudoh, S., Hasegawa, K., Niimori-Kita, K., and Ito, T. (2014). Notch1 signaling controls cell proliferation, apoptosis and differentiation in lung carcinoma. *Lung Cancer (Amsterdam, Netherlands)*, 85(2):131–140.

Wang, H., Yu, Z., Huo, S., Chen, Z., Ou, Z., Mai, J., Ding, S., and Zhang, J. (2018). Overexpression of ELF3 facilitates cell growth and metastasis through PI3K/Akt and ERK signaling pathways in non-small cell lung cancer. *The International Journal of Biochemistry & Cell Biology*, 94:98–106.

Wang, M., Han, J., Marcar, L., Black, J., Liu, Q., Li, X., Nagulapalli, K., Sequist, L. V., Mak, R. H., Benes, C. H., Hong, T. S., Gurtner, K., Krause, M., Baumann, M., Kang, J. X., Whetstine, J. R., and Willers, H. (2017). Radiation Resistance in KRAS-Mutated Lung Cancer Is Enabled by Stem-like Properties Mediated by an Osteopontin-EGFR Pathway. *Cancer Research*, 77(8):2018–2028.

Wang, X.-J., Sun, Z., Villeneuve, N. F., Zhang, S., Zhao, F., Li, Y., Chen, W., Yi, X., Zheng, W., Wondrak, G. T., Wong, P. K., and Zhang, D. D. (2008). Nrf2 enhances resistance of cancer cells to chemotherapeutic drugs, the dark side of Nrf2. *Carcinogenesis*, 29(6):1235–1243.

Xu, T., Li, D., Wang, H., Zheng, T., Wang, G., and Xin, Y. (2017a). MUC1 downregulation inhibits non-small cell lung cancer progression in human cell lines. *Experimental and Therapeutic Medicine*, 14(5):4443–4447.

Xu, X., Li, D., Liu, J., Ma, Z., Huang, H., Min, L., Dai, L., and Dong, S. (2019). Downregulation of PTPRK Promotes Cell Proliferation and Metastasis of NSCLC by Enhancing STAT3 Activation. *Analytical Cellular Pathology (Amsterdam)*, 2019:4265040.

Xu, Y., Jin, J., Xu, J., Shao, Y. W., and Fan, Y. (2017b). JAK2 variations and functions in lung adenocarcinoma. *Tumour Biology: The Journal of the International Society for Oncodevelopmental Biology and Medicine*, 39(6):1010428317711140.

Yang, L., Yang, X., Tang, Y., Zhang, D., Zhu, L., Wang, S., Wang, B., and Ma, T. (2018). Inhibition of DNA-PK activity sensitizes A549 cells to X-ray irradiation by inducing the ATM-dependent DNA damage response. *Molecular Medicine Reports*, 17(6):7545–7552.

You, L., He, B., Xu, Z., Uematsu, K., Mazieres, J., Mikami, I., Reguart, N., Moody, T. W., Kitajewski, J., McCormick, F., and Jablons, D. M. (2004). Inhibition of Wnt-2-mediated signaling induces programmed cell death in non-small-cell lung cancer cells. *Oncogene*, 23(36):6170–6174. Number: 36 Publisher: Nature Publishing Group.

Zhang, X., Guo, A., Yu, J., Possemato, A., Chen, Y., Zheng, W., Polakiewicz, R. D., Kinzler, K. W., Vogelstein, B., Velculescu, V. E., and Wang, Z. J. (2007). Identification of STAT3 as a substrate of receptor protein tyrosine phosphatase T. *Proceedings of the National Academy of Sciences of the United States of America*, 104(10):4060–4064.

Zhao, Z.-Q., Yu, Z.-Y., Li, J., and Ouyang, X.-N. (2016). Gefitinib induces lung cancer cell autophagy and apoptosis via blockade of the PI3K/AKT/mTOR pathway. *Oncology Letters*, 12(1):63–68.

Zhou, H., Mu, X., Chen, J., Liu, H., Shi, W., Xing, E., Yang, K., and Wu, G. (2013). RNAi silencing targeting RNF8 enhances radiosensitivity of a non-small cell lung cancer cell line A549. *International Journal of Radiation Biology*, 89(9):708–715.
