# Supplementary material for: Virtual Tumors Enable Prediction of Personalized Therapeutic Combinations for Non-Small Cell Lung Cancer
Source: Cancer Res. Author manuscript; Available in PMC 2026 Aug 11. (PMC7619348; doi:10.1158/0008-5472.CAN-25-2476)
Supplement: Supplementary file 15 [file EMS216192-supplement-24.pdf]

Supplementary Table S6. List of druggable nodes in the model and the drugs that target them.

Node	GeneSymbol	UNIPROT ID	FDA approval	chEMBL ID
4EBP1	EIF4EBP1	Q13541	FALSE	CHEMBL3351214, CHEMBL4296088
Akt	AKT1	P31749	FALSE	CHEMBL4523748, CHEMBL3038463, CHEMBL3885629, CHEMBL4106175, CHEMBL5169081, CHEMBL5169068, CHEMBL2111353, CHEMBL4282
AMPK	PRKAA2	P54646	TRUE	CHEMBL3038455, CHEMBL3885504, CHEMBL3038456, CHEMBL4106159, CHEMBL4106158, CHEMBL3038457, CHEMBL3038454, CHEMBL2116, CHEMBL2096907
APC	APC	D6RFL6	FALSE	
ARF	CDKN2A	P42771	FALSE	CHEMBL4680027
ATR	ATR	H0Y8Y6	FALSE	
BAD	BAD	Q92934	FALSE	CHEMBL5169266, CHEMBL3817
BAKBAX	BAK1	Q16611	FALSE	CHEMBL3885523, CHEMBL3885516, CHEMBL3137283, CHEMBL5609, CHEMBL5169269
BAKBAX	BAX	Q07812	FALSE	
Beta-Catenin	CTNNB1	C9IZ65	FALSE	
BCL-2	BCL2	P10415	TRUE	CHEMBL4523685, CHEMBL4748224, CHEMBL5169264, CHEMBL5169266, CHEMBL3885516, CHEMBL3885513, CHEMBL4860, CHEMBL5169265
BCL-W	BCL2L2	G3V3B7	FALSE	
BCL-XL	BCL2L1	Q07817	FALSE	CHEMBL3883285, CHEMBL3883286, CHEMBL3137283, CHEMBL4523705, CHEMBL3883287, CHEMBL4625, CHEMBL3883288, CHEMBL4748229
Bim	BCL2L11	C9J417	FALSE	
CDC25A	CDC25A	P30304	FALSE	CHEMBL3775
CDK2	CDK2	P24941	FALSE	CHEMBL4523633, CHEMBL2094128, CHEMBL3038470, CHEMBL3885552, CHEMBL2111326, CHEMBL4888454, CHEMBL4106153, CHEMBL4888444, CHEMBL4106152, CHEMBL3038517, CHEMBL4523689, CHEMBL301, CHEMBL1907605, CHEMBL2094126, CHEMBL4523634, CHEMBL3038469, CHEMBL3559691
CDKN2A	CDKN2A	P42771	FALSE	CHEMBL4680027
CHK1	CHEK1	E9PRU7	FALSE	
CHK2	CHEK1	E9PRU7	FALSE	
c-Jun	JUN	P05412	TRUE	CHEMBL4977, CHEMBL2111421
CycE	CCNE1	P24864	FALSE	CHEMBL2111444, CHEMBL4523715, CHEMBL3885554, CHEMBL3038471, CHEMBL1907605, CHEMBL2094126, CHEMBL3617, CHEMBL3038468
Dishevelled	DVL1	O14640	FALSE	CHEMBL6027
E2F	E2F1	Q01094	FALSE	CHEMBL4382, CHEMBL4630726
EGFR	EGFR	Q504U8	FALSE	
FOXM1	FOXM1	Q08050	FALSE	
FOXO3	FOXO3	O43524	FALSE	CHEMBL5778
GRB2	GRB2	J3QLF6	FALSE	
GSK3B	GSK3B	P49841	FALSE	CHEMBL3883309, CHEMBL262, CHEMBL2095188

Node	GeneSymbol	UNIPROT ID	FDA approval	chEMBL ID
JAK2	JAK2	O60674	FALSE	
KEAP1	KEAP1	Q14145	TRUE	CHEMBL4296122, CHEMBL4106129, CHEMBL4296123, CHEMBL2069156, CHEMBL3038498
LRP	LRP1	Q7Z7K9	FALSE	
MCL-1	MCL1	Q07820	FALSE	CHEMBL4523712, CHEMBL3430887, CHEMBL3885523, CHEMBL3430886, CHEMBL3885515, CHEMBL4361
MDM2	MDM2	Q00987	FALSE	CHEMBL4523719, CHEMBL4879538, CHEMBL4523702, CHEMBL3883306, CHEMBL3038493, CHEMBL4523720, CHEMBL4523718, CHEMBL5023, CHEMBL4888446, CHEMBL4106123, CHEMBL1907611
MEK	MAP2K1	Q02750	TRUE	CHEMBL4523703, CHEMBL3885566, CHEMBL4523755, CHEMBL4523740, CHEMBL3587, CHEMBL2111351, CHEMBL2111289
MET	MET	C9JKM5	FALSE	
mTORC1	MTOR	B1AKP8	FALSE	
mTORC2	MTOR	B1AKP8	FALSE	
NFkB	NFKB1	P19838	TRUE	CHEMBL2094258, CHEMBL3251
NRF2	NFE2L2	H7C498	FALSE	
p21	CDKN1A	P38936	FALSE	
p27	CDKN1B	P46527	FALSE	
p53	TP53	E7EQX7	FALSE	
p70S6K	RPS6KB1	K7ER06	FALSE	
p90RSK	RPS6KA1	E9PPC1	FALSE	
Plk1	PLK1	P53350	FALSE	
pRB	RB1	P06400	FALSE	
PTEN	PTEN	P60484	FALSE	CHEMBL4523606, CHEMBL2052032
PUMA	BBC3	Q9BXH1	FALSE	
RAF	RAF1	P04049	TRUE	CHEMBL4106189, CHEMBL3885566, CHEMBL2111351, CHEMBL3883317, CHEMBL3559685, CHEMBL1906
RAF	BRAF	H7C560	FALSE	
RAS	KRAS	P01116	TRUE	CHEMBL5169273, CHEMBL4523623, CHEMBL2189121, CHEMBL4524006
STAT3	STAT3	P40763	FALSE	CHEMBL4296101, CHEMBL4026, CHEMBL4523691
SOS	SOS1	Q07889	FALSE	CHEMBL2079846, CHEMBL5169070
STK11	STK11	A0A087WT72	FALSE	
Wee1	WEE1	P30291	FALSE	CHEMBL5169067, CHEMBL4630732, CHEMBL5491
YB1	YBX1	P67809	FALSE	CHEMBL4296006
Caspase3	CASP3	A8MVM1	FALSE	
CDK1	CDK1	E5RIU6	FALSE	
CycB	CCNB1	P14635	FALSE	CHEMBL2412, CHEMBL4523630, CHEMBL4680053, CHEMBL1907602, CHEMBL2094127
CycD	CCND1	P24385	TRUE	CHEMBL2095942, CHEMBL3885552, CHEMBL1907601, CHEMBL3610, CHEMBL4523688, CHEMBL3885551, CHEMBL2111455

Node	GeneSymbol	UNIPROT ID	FDA approval	chEMBL ID
CDK4	CDK4	P11802	TRUE	CHEMBL3038472, CHEMBL2095942, CHEMBL3301385, CHEMBL2111326, CHEMBL4888444, CHEMBL4523963, CHEMBL4106184, CHEMBL4630748, CHEMBL3038517, CHEMBL4523715, CHEMBL3885554, CHEMBL4523686, CHEMBL4523732, CHEMBL1907601, CHEMBL3885553, CHEMBL3885548, CHEMBL3559691, CHEMBL331
hTERT	TERT	O14746	FALSE	
p16	CDKN2A	P42771	FALSE	CHEMBL4680027
Erbb1	EGFR	Q504U8	FALSE	
Erbb3	ERBB3	F8VRL0	FALSE	
CDC25C	CDC25C	P30307	FALSE	CHEMBL2378
ATM	ATM	E9PIN0	FALSE	
Fyn	FYN	P06241	FALSE	CHEMBL2363074, CHEMBL1841
IL-6	IL6	P05231	TRUE	CHEMBL1795129
PKA	PRKACA	P17612	FALSE	CHEMBL4101, CHEMBL2094138
CUL3	CUL3	Q13618	FALSE	CHEMBL4296124, CHEMBL4296123
RBX1	RBX1	P62877	FALSE	
MYC	MYC	A0A0B4J1R1	FALSE	
MAX	MAX	P61244	FALSE	CHEMBL1250363, CHEMBL4106127, CHEMBL3301395
Ku	XRCC6	P12956	FALSE	CHEMBL4106136
Ku	XRCC5	P13010	FALSE	CHEMBL4106136
DNA-PKcs	PRKDC	P78527	FALSE	CHEMBL4106136, CHEMBL3142
MRE11	MRE11	P49959	FALSE	
NBS1	NBN	O60934	FALSE	CHEMBL5169101
14_3_3	YWHAZ	P63104	FALSE	CHEMBL4105899
RAD51	RAD51	E9PNT5	FALSE	
53BP1	TP53BP1	Q12888	FALSE	CHEMBL2424509
BRCA1	BRCA1	P38398	FALSE	CHEMBL5990
XRCC4	ERCC4	Q92889	FALSE	CHEMBL3883316
LIG4	LIG4	P49917	FALSE	CHEMBL4296097, CHEMBL4523595
KU70	XRCC6	P12956	FALSE	CHEMBL4106136
KU80	XRCC5	P13010	FALSE	CHEMBL4106136
RNF168	RNF168	Q8IYW5	FALSE	CHEMBL5169175
ELF3	ELF3	P78545	FALSE	CHEMBL2111426
SKP2	SKP2	Q13309	FALSE	CHEMBL3885557, CHEMBL3885558, CHEMBL3885634
Caspase9	CASP9	F8VVS7	FALSE	
Frizzled	FZD1	Q9UP38	FALSE	CHEMBL2346493
CBF	RBPJ	D6RAT2	FALSE	
NOTCH1	NOTCH1	P46531	FALSE	CHEMBL4524007, CHEMBL2146346

Supplementary Table S6. List of druggable nodes in the *in silico* model and the drugs that target them. Each row is a node from the model, with the gene symbol, Uniprot ID and whether that protein as an FDA approved drug targeting it. Additionally, those drugs with chemical probes are shown using the chEMBL database.
