# Supplementary material for: Virtual Tumors Enable Prediction of Personalized Therapeutic Combinations for Non-Small Cell Lung Cancer
Source: Cancer Res. Author manuscript; Available in PMC 2026 Aug 11. (PMC7619348; doi:10.1158/0008-5472.CAN-25-2476)
Supplement: Supplementary file 16 [file EMS216192-supplement-25.pdf]

Supplementary Table S7. List of drugs used in Nair et al. (2023). and their corresponding model targets.

DrugID	Drug Name	Primary target
1	Erlotinib	EGFR
14	Neratinib	Erbb1
29	AZ628	RAF
37	Crizotinib	MET
60	BI-2536	Plk1
75	RAF265	RAF
94	TGX 221	PI3K
96	Pimozide	STAT3
97	Selumetinib	MEK
99	Vandetanib	EGFR
113	Alpelisib	PI3K
119	Lapatinib	Erbb1
125	Dactolisib	PI3K
154	CHIR-99021	GSK3B
156	AZD6482	PI3K
182	Obatoclax	BCL-2
210	Vemurafenib	RAF
224	AS605240	PI3K
238	Idelalisib	PI3K
246	Nutlin	MDM2
249	Cabozantinib	MET
283	Omipalisib	PI3K
306	Fedratinib	JAK2
326	GSK690693	Akt
357	MK-2206	Akt
368	Afatinib	Erbb1
373	Pictilisib	PI3K
385	Navitoclax	BCL-2
398	VE-821	ATR
412	Venetoclax	BCL-2
421	KU-55933	ATM
429	Dabrafenib	RAF
430	Trametinib	MEK
446	MIM1	MCL-1
461	NSC319726	p53
519	Mirin	MRE11
522	RO-3306	CDK1
527	AT9283	JAK2
530	Serdemetan	MDM2
537	SCH900776	CHK1
538	KU-60019	ATM
564	MK-8033	MET
589	Tivantinib	MET

Supplementary Table S7. List of drugs used in Nair et al. (2023) and their corresponding model targets. Drug name is shown along with its target and ID used in Nair et al. (2023).

Table References

Nair, N. U., Greninger, P., Zhang, X., Friedman, A. A., Amzallag, A., Cortez, E., Sahu, A. D., Lee, J. S., Dastur, A., Egan, R. K., Murchie, E., Ceribelli, M., Crowther, G. S., Beck, E., McClanaghan, J., Klump-Thomas, C., Boisvert, J. L., Damon, L. J., Wilson, K. M., Ho, J., Tam, A., McKnight, C., Michael, S., Itkin, Z., Garnett, M. J., Engelman, J. A., Haber, D. A., Thomas, C. J., Ruppin, E., and Benes, C. H. (2023). A landscape of response to drug combinations in non-small cell lung cancer. *Nature Communications*, 14(1):3830. Number: 1 Publisher: Nature Publishing Group.
