# Supplementary material for: Virtual Tumors Enable Prediction of Personalized Therapeutic Combinations for Non-Small Cell Lung Cancer
Source: Cancer Res. Author manuscript; Available in PMC 2026 Aug 11. (PMC7619348; doi:10.1158/0008-5472.CAN-25-2476)
Supplement: Supplementary file 17 [file EMS216192-supplement-5.pdf]

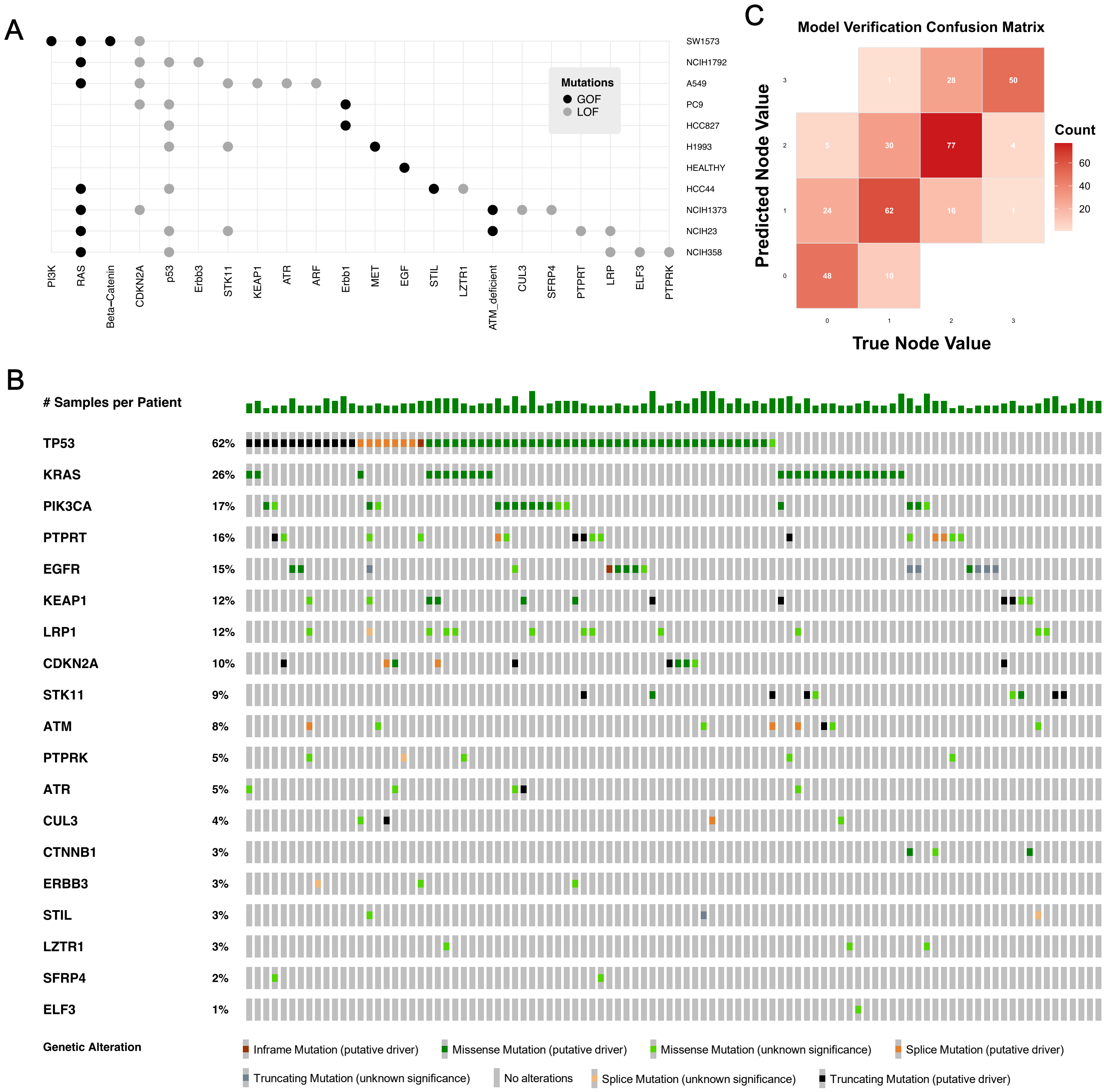

Supplementary Figure S1. Summary of mutations in the modeled cell lines. A) The schematic (right) shows mutations as columns and cell lines as rows, with gain-of-function mutations for a cell line and loss-of-function (grey). **B)** Schematic showing the presence of these mutations across clinical data (Plotted using cBio-Portal, using studies from TRACERx (Abbosh et al. (2017); Jamal-Hanjani et al. (2017))). **C)** Confusion matrix showing the simulated results of the model (y-axis) with the correct activity levels specified in the ground truth (x-axis), achieving a Quadratic Weighted Kappa (QWK) score of 0.81. The color indicates the number of experiments in each bin.

Figure References

Abbosh, C., Birkbak, N. J., Wilson, G. A., Jamal-Hanjani, M., Constantin, T., Salari, R., Le Quesne, J., Moore, D. A., Veeriah, S., Rosenthal, R., et al. (2017). Phylogenetic ctdna analysis depicts early-stage lung cancer evolution. *Nature*, 545(7655):446–451.

Jamal-Hanjani, M., Wilson, G. A., McGranahan, N., Birkbak, N. J., Watkins, T. B., Veeriah, S., Shafi, S., Johnson, D. H., Mitter, R., Rosenthal, R., et al. (2017). Tracking the evolution of non–small-cell lung cancer. *New England Journal of Medicine*, 376(22):2109–2121.
