# Supplementary material for: Virtual Tumors Enable Prediction of Personalized Therapeutic Combinations for Non-Small Cell Lung Cancer
Source: Cancer Res. Author manuscript; Available in PMC 2026 Aug 11. (PMC7619348; doi:10.1158/0008-5472.CAN-25-2476)
Supplement: Supplementary file 18 [file EMS216192-supplement-6.pdf]

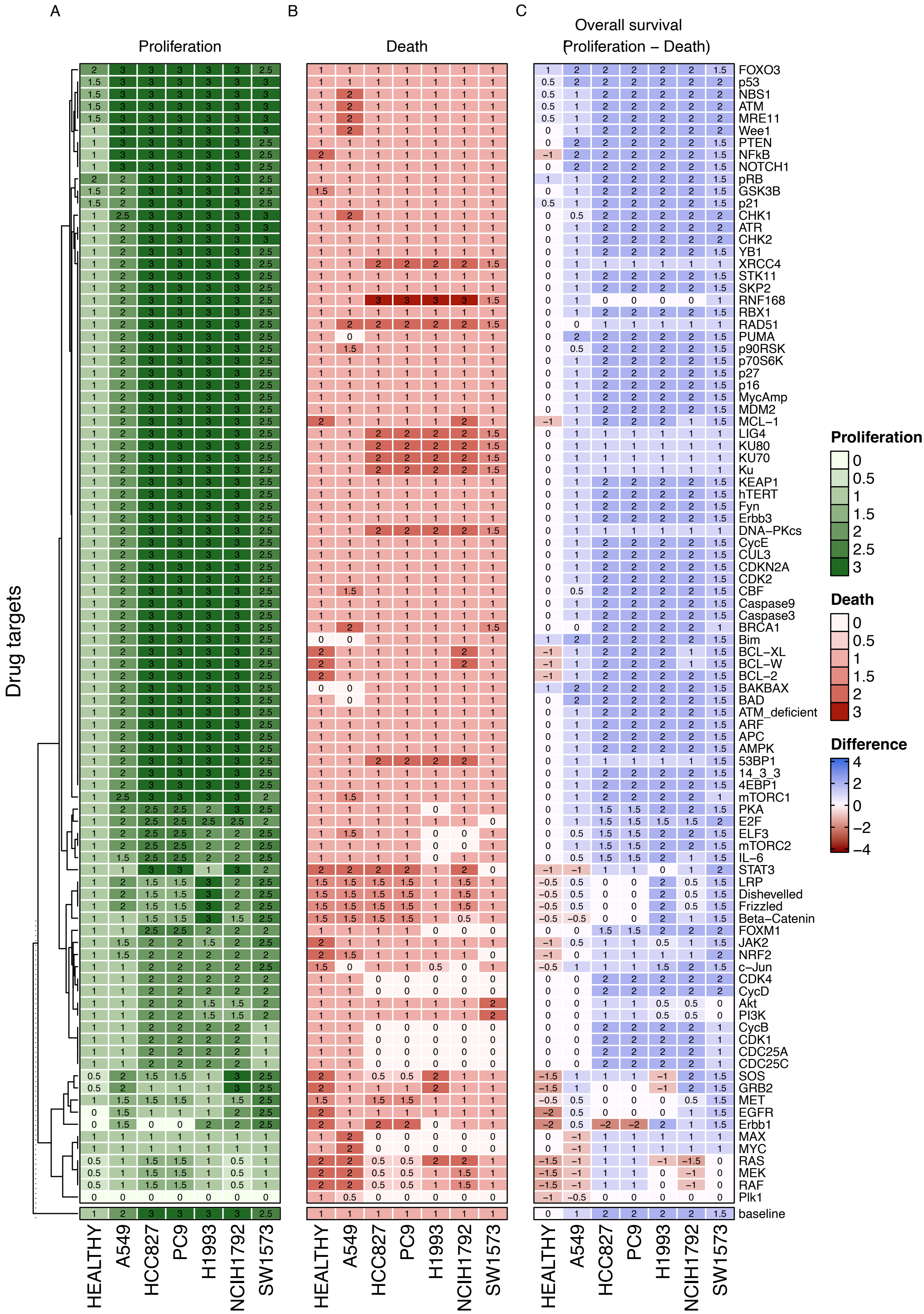

Supplementary Figure S2. Heatmap showing the simulation of the model after perturbation with single drug treatments. The columns are various cancer cell lines with healthy cells at the far left. Rows indicate druggable proteins that are included in the model. Values in the model correspond to proliferation (**A**, in green), death (**B**, in red) and the difference between proliferation and death (**C**, red to blue). For the difference between them, positive values (blue) indicates growth and negative values (red) indicate death. The baseline values for cell lines (readings without any perturbation) is shown on the bottom of the heatmaps.
