# Supplementary material for: Virtual Tumors Enable Prediction of Personalized Therapeutic Combinations for Non-Small Cell Lung Cancer
Source: Cancer Res. Author manuscript; Available in PMC 2026 Aug 11. (PMC7619348; doi:10.1158/0008-5472.CAN-25-2476)
Supplement: Supplementary file 19 [file EMS216192-supplement-7.pdf]

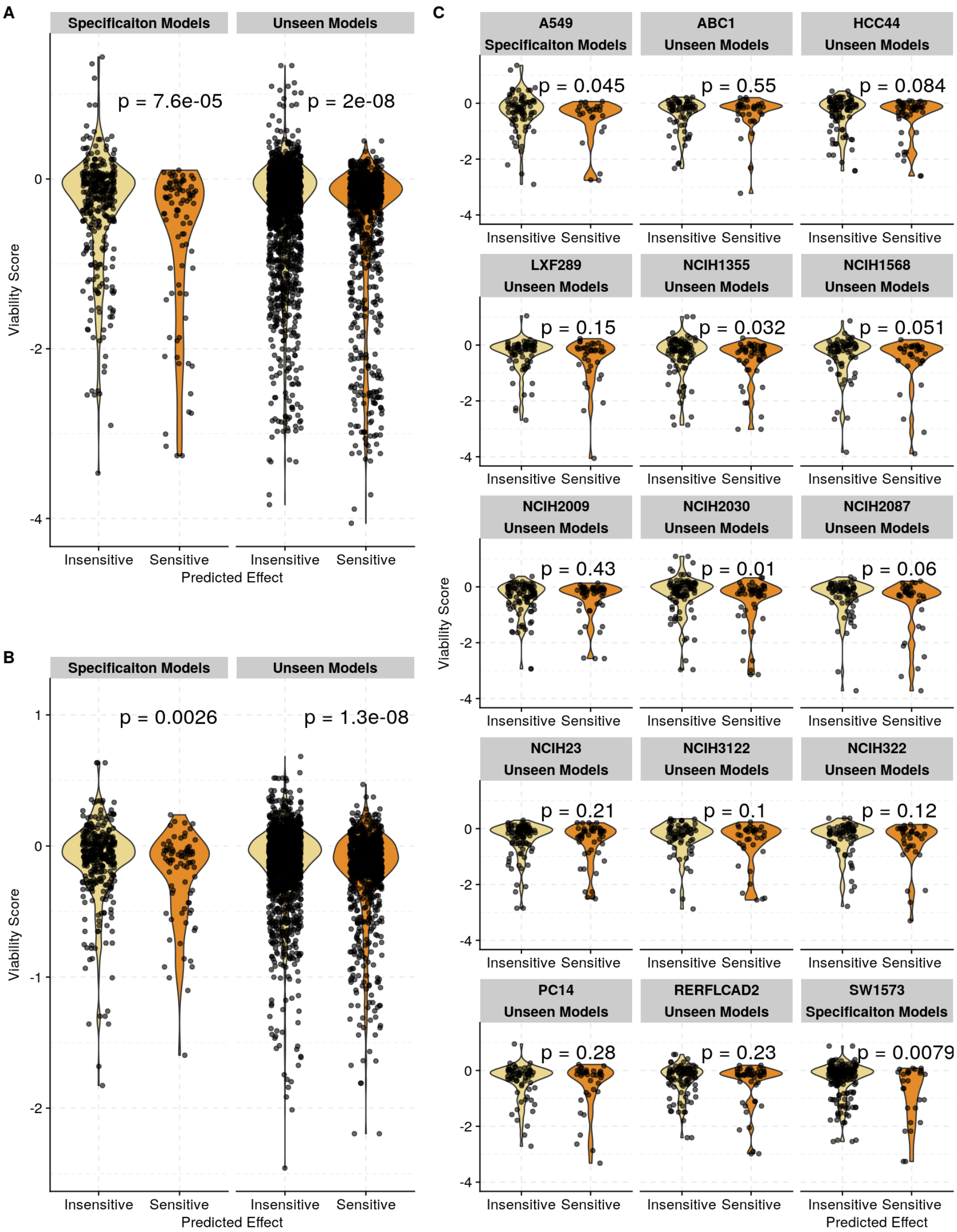

Supplementary Figure S3. Plots show comparison of single-perturbation predicted screens to DepMap experimental data. A-B) Predicted sensitivity and insensitivity calls from the model simulations compared with CRISPR (Chronos) gene-dependency scores (**A**) and RNAi (DEMETER2) dependency scores (**B**) from DepMap. Each violin shows the distribution of observed viability (dependency) scores for predicted "Sensitive" versus "Insensitive" cell lines, separated by whether or not the cell line was included in the specification. P-values from one-sided Wilcoxon rank-sum tests show whether predicted sensitive lines have lower viability. **C)** Per-cell-line view of CRISPR validation results showing whether the cell line was part of the training ("Specification Models") or test ("Unseen Models") cohort.
