# Supplementary material for: Virtual Tumors Enable Prediction of Personalized Therapeutic Combinations for Non-Small Cell Lung Cancer
Source: Cancer Res. Author manuscript; Available in PMC 2026 Aug 11. (PMC7619348; doi:10.1158/0008-5472.CAN-25-2476)
Supplement: Supplementary file 20 [file EMS216192-supplement-8.pdf]

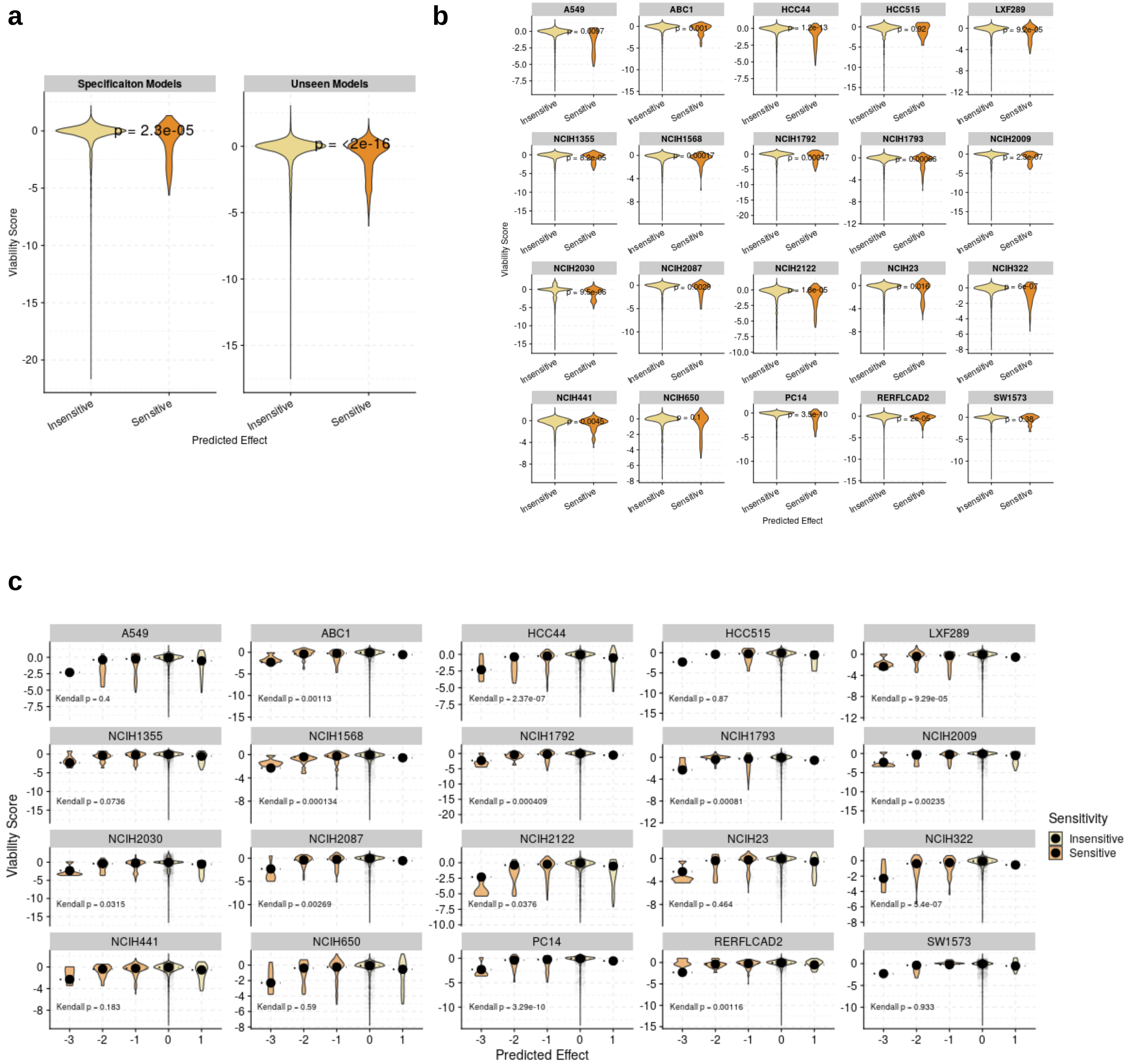

Supplementary Figure S4. Validation of predicted drug sensitivities against DepMap PRISM screening data. A) Distribution of PRISM viability scores for compounds targeting model nodes, stratified by predicted "Sensitive" versus "Insensitive" responses and shown per cell line. Each violin represents the distribution of observed drug dependencies within a cell line; p-values from one-sided Wilcoxon rank-sum tests indicate whether predicted sensitive lines exhibit lower viability. **(B)** Aggregated comparison of predicted sensitivity versus PRISM viability, separated by cell line **(C)** More conservative ordinal analysis of predicted effect strength, showing PRISM viability scores across discrete predicted effect bins per cell line. Black points and dotted lines indicate median viability per bin, and Kendall's tau p-values quantify monotonic association between predicted effect magnitude and experimental drug response within each cell line.
