# Supplementary material for: Virtual Tumors Enable Prediction of Personalized Therapeutic Combinations for Non-Small Cell Lung Cancer
Source: Cancer Res. Author manuscript; Available in PMC 2026 Aug 11. (PMC7619348; doi:10.1158/0008-5472.CAN-25-2476)
Supplement: Supplementary file 21 [file EMS216192-supplement-9.pdf]

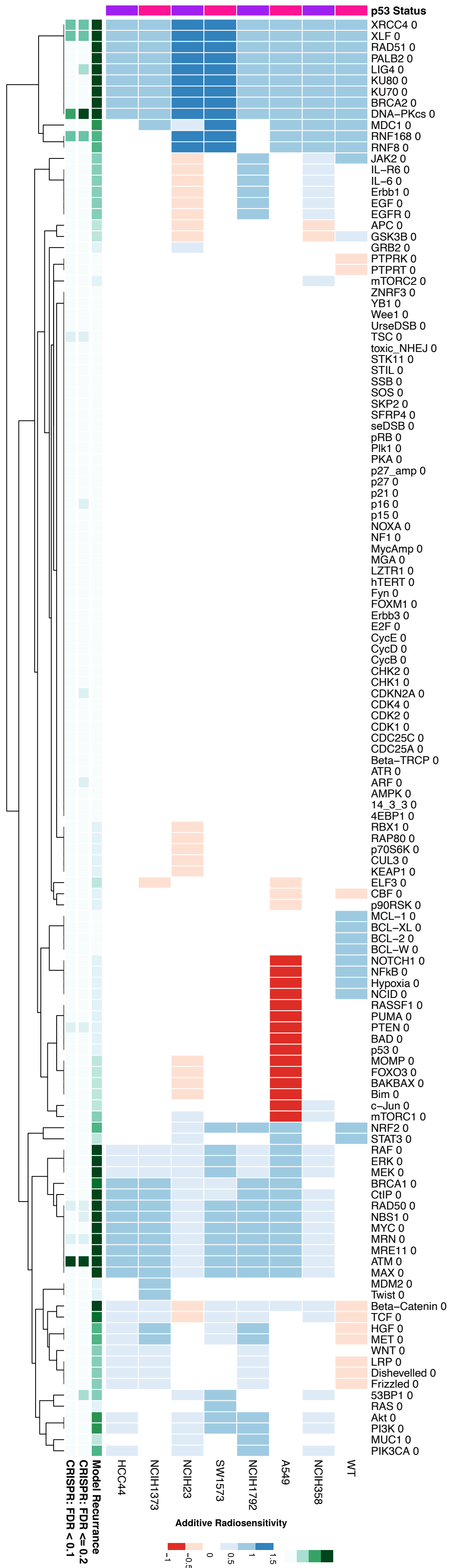

Supplementary Figure S5. Systematic screen of perturbations on the *in silico* model. Heatmap showing predicted radio-sensitizing effect in silico knockouts (rows, node value set to 0), on NSCLC adenocarcinoma cell lines (columns). Additive radio-sensitivity (see Methods) is increase (blue) or decrease (red) in cell death in the combination of radiotherapy and knock-out compared to the most effective of radiotherapy or knock-out applied alone. Side panels (green) show the number of cell lines where treatment was radio-sensitizing in the model or CRISPR screen (at FDR ≤ 0.2 and < 0.1). We count a recurrence if radio-sensitivity is observed at this threshold for any time-point in the screen.
